# Rare Glutamic Acid Methyl Ester Peptaibols from *Sepedonium ampullosporum* Damon KSH 534 Exhibit Promising Antifungal and Anticancer Activity

**DOI:** 10.3390/ijms222312718

**Published:** 2021-11-24

**Authors:** Yen T. H. Lam, Manuel G. Ricardo, Robert Rennert, Andrej Frolov, Andrea Porzel, Wolfgang Brandt, Pauline Stark, Bernhard Westermann, Norbert Arnold

**Affiliations:** 1 Department of Bioorganic Chemistry, Leibniz Institute of Plant Biochemistry, D-06120 Halle (Saale), Germany; ThiHaiYen.Lam@ipb-halle.de (Y.T.H.L.); Manuel.GarciaRicardo@mpikg.mpg.de (M.G.R.); Robert.Rennert@ipb-halle.de (R.R.); Andrej.Frolov@ipb-halle.de (A.F.); Andrea.Porzel@ipb-halle.de (A.P.); Wolfgang.Brandt@ipb-halle.de (W.B.); Pauline.Stark@ipb-halle.de (P.S.); Bernhard.Westermann@ipb-halle.de (B.W.); 2 Department of Organic Chemistry, Faculty of Chemistry, Hanoi National University of Education, Hanoi 100000, Vietnam; 3 Department of Biomolecular Systems, Max Planck Institute of Colloids and Interfaces, D-14476 Potsdam, Germany; 4 Department of Biochemistry, Faculty of Biology, St. Petersburg State University, 199004 St. Petersburg, Russia

**Keywords:** *Sepedonium ampullosporum*, peptaibols, ampullosporin, glutamic acid methyl ester, solid-phase peptide synthesis, antifungal, anticancer, molecular docking

## Abstract

Fungal species of genus *Sepedonium* are rich sources of diverse secondary metabolites (e.g., alkaloids, peptaibols), which exhibit variable biological activities. Herein, two new peptaibols, named ampullosporin F (**1**) and ampullosporin G (**2**), together with five known compounds, ampullosporin A (**3**), peptaibolin (**4**), chrysosporide (**5**), c(Trp-Ser) (**6**) and c(Trp-Ala) (**7**), have been isolated from the culture of *Sepedonium ampullosporum* Damon strain KSH534. The structures of **1** and **2** were elucidated based on ESI-HRMS^n^ experiments and intense 1D and 2D NMR analyses. The sequence of ampullosporin F (**1**) was determined to be Ac-Trp^1^-Ala^2^-Aib^3^-Aib^4^-Leu^5^-Aib^6^-Gln^7^-Aib^8^-Aib^9^-Aib^10^-GluOMe^11^-Leu^12^-Aib^13^-Gln^14^-Leuol^15^, while ampullosporin G (**2**) differs from **1** by exchanging the position of Gln^7^ with GluOMe^11^. Furthermore, the total synthesis of **1** and **2** was carried out on solid-phase to confirm the absolute configuration of all chiral amino acids as L. In addition, ampullosporin F (**1**) and G (**2**) showed significant antifungal activity against *B. cinerea* and *P. infestans*, but were inactive against *S. tritici*. Cell viability assays using human prostate (PC-3) and colorectal (HT-29) cancer cells confirmed potent anticancer activities of **1** and **2**. Furthermore, a molecular docking study was performed in silico as an attempt to explain the structure-activity correlation of the characteristic ampullosporins (**1**–**3**).

## 1. Introduction

Filamentous fungi from saprophytic genera, e.g., *Acremonium*, *Trichoderma*/*Hypocrea* and *Sepedonium*, are known for a high abundance of nonribosomal-peptide-synthetase (NRPS)-derived metabolites, so-called pept”Aib”ols [1]. This class of compounds is defined as linear peptides with 5−20 amino acids (AA) residues including (*i*) a high proportion of the nonproteinogenic α,α-dialkylated amino acid α-aminoisobutyric acid (Aib); (*ii*) an *N*-acetyl terminus; (*iii*) and a *C*-terminal AA reduced into amino alcohol such as leucinol (Leuol) or phenylalaninol (Pheol) [1]. Peptaibols are intriguing not only because of the structural variability generated by varying amino acid building blocks, but also due to their broad range of bioactivities, i.e., cytotoxic [2,3,4,5,6,7,8,9,10,11], antibacterial [2,5,6,10,11,12,13], antiviral [14,15], antileishmanial [16], antifungal [5,6,17,18,19], plant root growth inhibiting [20], insecticidal [7], and anthelmintic activity [2]. Furthermore, ampullosporin A (**3**), a peptaibol isolated from *Sepedonium ampullosporum* Damon, has been shown to permit neuroleptic-like activity in mice [21,22], whereby it exhibited a more targeted interaction with the glutamatergic system, namely the *N*-methyl-D-aspartate (NMDA) receptor [23].

These biological activities appear to be related to the strong foldameric capacity of Aib so that peptaibols would adopt helical structures in artificial bilayers and natural membranes [24]. Consequently, the amphipathic voltage-dependent helices can act as ion channels in cell membranes, causing cytoplasmic leakage and cellular breakdown [24,25].

The genus *Sepedonium* (teleomorph *Hypocrea*, Ascomycota), which was established by H.F. Link and restricted to mold-like fungal parasites, is characterized by the occurrence of two synanamorphs, i.e., aleurioconidia (for persistence) and phialoconidia (for fast propagation). *Sepedonium* species were found settling on basidiocarps of Boletales s.l. [26]. Unlike the intensively studied genus *Trichoderma*/*Hypocrea*, there are only few reports on secondary metabolites isolated from *Sepedonium* spp. So far, compounds belonging to tropolones [27,28], anthraquinones (mono-, dimeric) [29], isoquinoline alkaloids [30], azaphilones [31], cyclopeptides [32,33], and peptaibols [15,17,18,19,21,22,34,35,36,37] are identified from species in this genus. Therefore, further studies on chemical components of *Sepedonium* spp. are promising for novel bioactive compounds, especially for new peptaibols.

During our ongoing work to study secondary metabolites from *Sepedonium* species, the culture broth and mycelial extract of *S. ampullosporum* Damon strain KSH 534 was investigated. So far, the chemical studies on *S. ampullosporum* strains have resulted in the isolation of characteristic 15-residue peptaibols, named ampullosprins A–D and E1–E4 [21,22], together with peptaibolin [35] and ampullosine [30].

The present paper describes the MS-guided isolation, structural elucidation, and total synthesis of two new linear 15-residue peptaibols, named ampullosporin F (**1**) and G (**2**), together with five known compounds including two linear peptaibols, ampullosporin A (**3**) and peptaibolin (**4**), as well as three cyclic peptides, chrysosporide (**5**), c(Trp-Ser) (**6**), and c(Trp-Ala) (**7**), from the semi-solid culture of *S. ampullosporum* Damon strain KSH 534. The absolute configuration of compounds **1** and **2** was determined by comparison to their synthetic counterparts. Moreover, the linear 15-residue peptaibols **1**–**3** were evaluated for their biological activity against the plant-pathogenic organisms *Botrytis cinerea* Pers., *Septoria tritici* Desm., and *Phytophthora infestans* (Mont.) de Bary, as well as their anticancer effects against human prostate (PC-3) and colorectal (HT-29) cancer cells. Additionally, the relationship between changes in amino acid sequence and activity of compounds **1**–**3** are discussed using a chemoinformatic molecular docking approach.

## 2. Results and Discussion

### 2.1. Isolation and Structural Elucidation of Compounds ***1**–**7***

**Figure 1:**
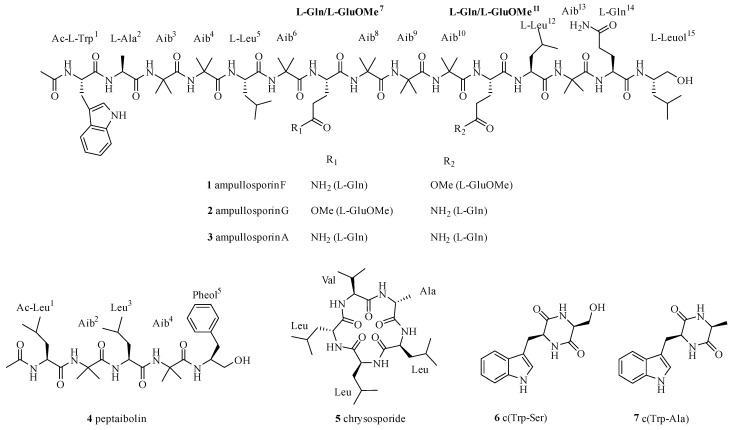
Structures of compounds **1**–**7**.

The chromatographic separation of the culture broth and mycelial crude extract using Diaion HP 20 and Sephadex LH 20 in combination with (semi)preparative HPLC yielded seven compounds (**1**−**7**) (Figure 1).

**Figure 2:**
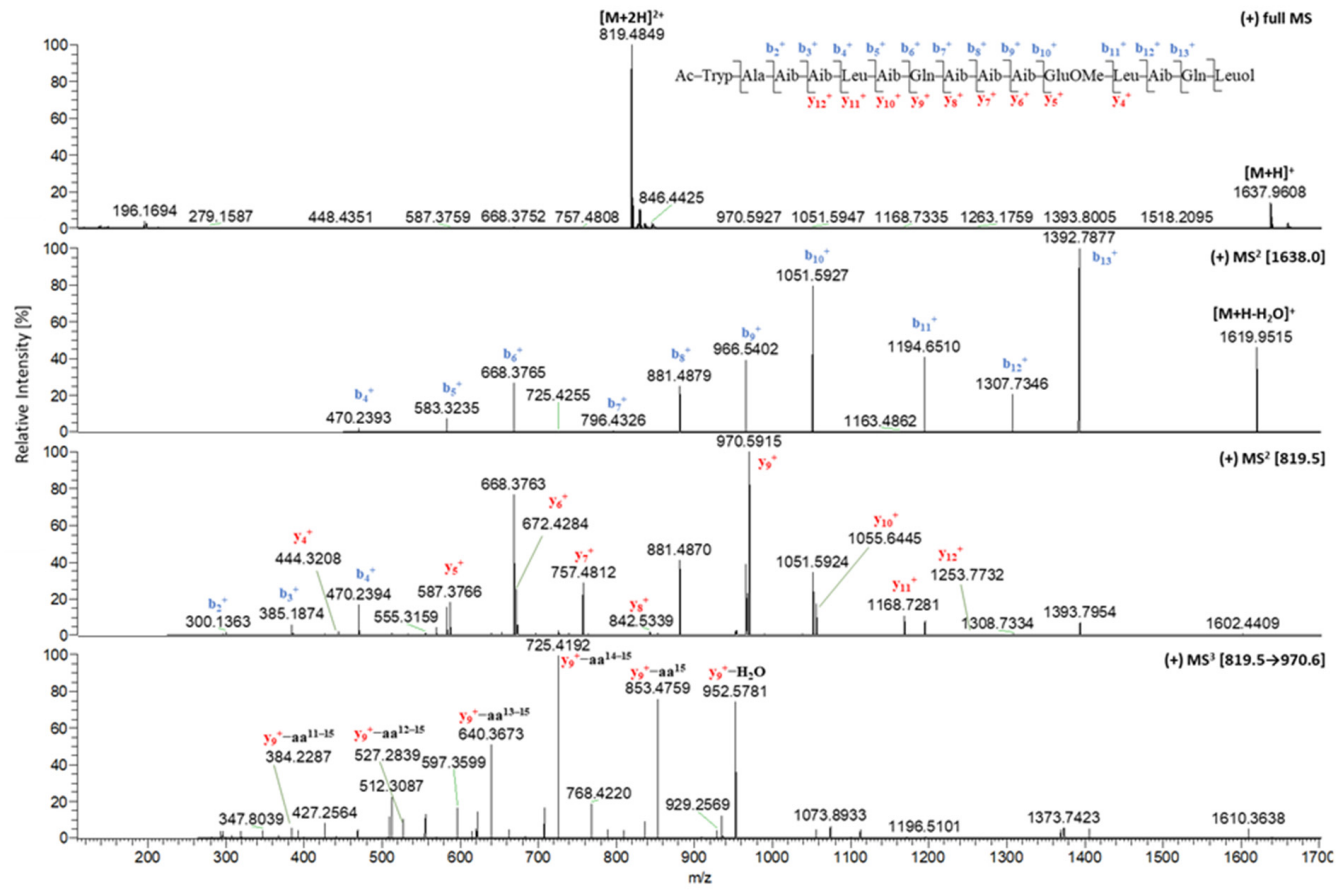
Positive ion ESI-HRMS^n^ spectra of ampullosporin F (**1**).

**Figure 3:**
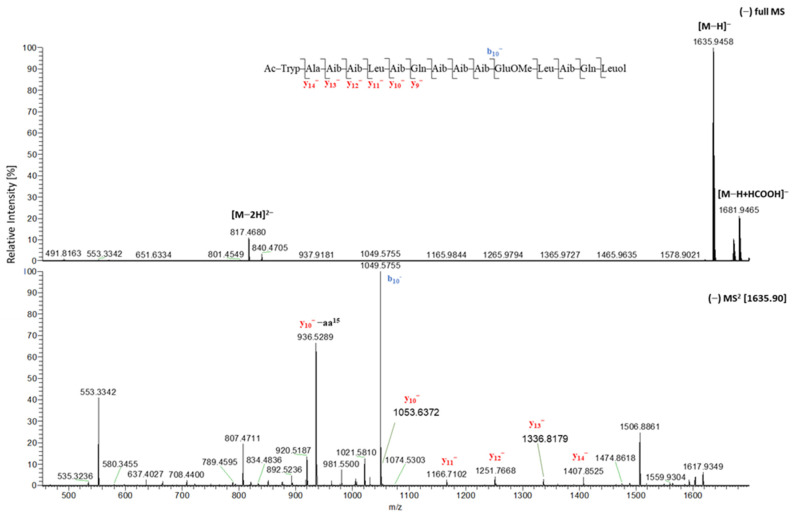
Negative ion ESI-HRMS^n^ spectra of ampullosporin F (**1**).

Compound **1** was isolated as a white, amorphous solid. The amino acid sequence of **1** was determined based on positive and negative ion ESI-HRMS^n^ studies, which showed diagnostic fragments of the b and y series (Figure 2 and Figure 3; Table 1; Table S1, Supplementary Materials).

The signal of the [M + 2H]^2+^ ion at *m*/*z* 819.4849 (calcd for C_78_H_130_N_18_O_20_^2+^ 819.4849) was the most intense one in the positive mode full scan spectrum of **1**, followed by the signal for the [M + H]^+^ ion at *m*/*z* 1637.9608 (calcd for C_78_H_129_N_18_O_20_^+^ 1637.9625), both corresponding to the molecular formula C_78_H_128_N_18_O_20_.

Fragmentation of the [M + H]^+^ ion generated a series of product ions b_4_^+^ to b_13_^+^, providing successive losses of Lxx^5^, Aib^6^, Gln^7^, Aib^8^, Aib^9^, Aib^10^, GluOMe^11^, Lxx^12^ and Aib^13^. The MS^2^ spectrum of the [M + 2H]^2+^ ion displayed further *N*-terminal b ions b_2_^+^ and b_3_^+^ corresponding to Aib^3^ and Aib^4^. The negative ion MS^2^ spectrum of the [M − H]^−^ yielded diagnostic fragment ions y_13_^−^ and y_14_^−^ representing Ala^2^ and Ac-Trp^1^ as the acetylated *N*-terminal amino acid. In addition, the observed series of *C*-terminal ions y_4_^+^ to y_12_^+^ and y_9_^−^ to y_12_^−^ fully supported the sequence deduced from b series. Thus, the *N*-terminal peptide part was shown to be Ac-Trp^1^-Ala^2^-Aib^3^-Aib^4^-Lxx^5^-Aib^6^-Gln^7^-Aib^8^-Aib^9^-Aib^10^-GluOMe^11^-Lxx^12^-Aib^13^.

Furthermore, the MS^3^ spectrum of the *C*-terminal ion y_9_^+^ displayed the characteristic mass differences of 117 amu (y_9_^+^-aa^15^, *m*/*z* 853.4770) and 245 amu (y_9_^+^-aa^14–15^, *m*/*z* 725.4192) corresponding to Isoleucinol/Leucinol (Lxxol^15^) as a *C*-terminal amino acid, and hence revealed the presence of Gln^14^. Based on the mass spectrometric analyses, the tentative sequence of **1** was proposed as Ac-Trp^1^-Ala^2^-Aib^3^-Aib^4^-Lxx^5^-Aib^6^-Gln^7^-Aib^8^-Aib^9^-Aib^10^-GluOMe^11^-Lxx^12^-Aib^13^-Gln^14^-Lxxol^15^.

The sequence of **1** resulting from mass spectrometry fragmentations was confirmed by 1D and 2D NMR data, which simultaneously specified the isomeric residues Leu and Ile.

The ^1^H spectrum of **1** (Table 2) displayed resonances of twenty exchangeable amide protons in the range of 6.7–11 ppm, including one broad low-field singlet (*δ*_H_ 10.86, Trp^1^), eight doublets, seven singlets representing seven Aib, as well as four broad singlets displaying two side-chain N-H_2_ (*δ*_H_ 7.12 and 6.75, Gln^7^; *δ*_H_ 7.13 and 6.73, Gln^14^), which were assigned in combination with ^1^H-^15^N HSQC data. In addition, five aromatic protons (*δ*_H_ 7.22, 7.56, 7.33, 7.05, and 6.96, Trp^1^) characterized the indole ring of tryptophan. In the high-field, multiple broad singlets between 1.30–1.50 ppm for Aib residues and a doublet for Ala^2^ (*δ*_H_ 1.27, d, 7.4 Hz) were observed, while six characteristic doublets (*δ*_H_ 0.93, 0.86 (×2), 0.84, 0.83, and 0.81; each *J* = 6.6 Hz) were unambiguously assigned to CH_3_ groups of Leu^5^, Leu^12^ and Leuol^15^.

**Figure 4:**
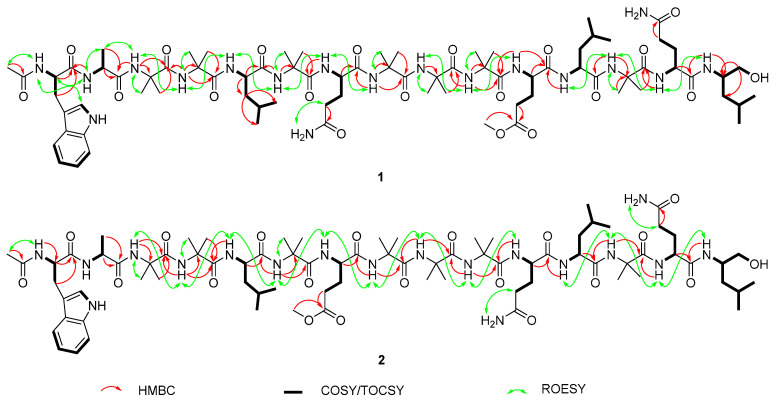
Key HMBC (H to C), COSY, and ROE correlations of ampullosporin F (**1**) and G (**2**).

As depicted in Figure 4, ^1^H-^1^H correlations from TOCSY and COSY spectra allowed defining eight N-H doublet peaks as part of eight spin systems, of which five correspond to one Ala, two Leu, and two Gln residues. Another leucine-related coupling system showing additional hydroxymethylene signals at *δ*_H_ 3.30/3.18, (*δ*_C_ 63.5) and a hydroxyl resonance at *δ*_H_ 4.50 bound to its methine at *δ*_H_ 3.78 (*δ*_C_ 48.2), demonstrated the presence of the reduced *C*-terminal Leuol residue. An additional moiety was pointed out by ^1^H-^1^H TOCSY correlations between the N-H amide group at *δ*_H_ 8.38, the methine at *δ*_H_ 4.40 (*δ*_C_ 54.4), and the methylene at *δ*_H_ 3.12/2.98, (*δ*_C_ 26.8). ^1^H-^13^C HMBC correlations demonstrated that this methylene carbon is linked to protons of the indole ring. Additionally, the ROESY correlation between the above amide at *δ*_H_ 8.38 and the acetyl CH_3_ group at *δ*_H_ 1.86 (*δ*_C_ 22.2), which showed HMBC correlation to a carbonyl carbon at *δ*_C_ 170.2, clearly resulted in the characterization of an acetylated *N*-terminal Trp residue. Finally, the *δ*-methylene of a glutamine-related spin system exhibited HMBC correlation to a carbonyl carbon at *δ*_C_ 173.0, which itself showed a strong HMBC correlation with a methoxy group at *δ*_H_ 3.55 (*δ*_C_ 50.8), supporting the presence of a glutamic acid *δ*-methyl ester residue.

Seven N-H singlets (*δ*_H_ 8.46, 8.06, 7.99, 7.96, 7.87, 7.57, and 7.45) were assigned for seven Aib residues due to their HMBC correlations to seven characteristic quaternary C-α carbons (*δ*_C_ 55.1–56.0) and seven carbonyl carbons (*δ*_C_ 173.4–176.3), as well as their ROESY interactions with methyl broad singlet peaks (*δ*_H_ 1.30–1.50/*δ*_C_ 22.0–26.2).

Detailed analysis of HMBC interactions of N-H resonances with carbonyl and C-α signals coupled with ROESY correlations between neighboring proton signals afforded the sequence establishment as in accordance with mass spectrometry fragmentations. Therefore, the structure of **1** was established as Ac-Trp^1^-Ala^2^- Aib^3^-Aib^4^-Leu^5^-Aib^6^-Gln^7^-Aib^8^-Aib^9^-Aib^10^-GluOMe^11^-Leu^12^-Aib^13^-Gln^14^-Leuol^15^, and named as ampullosporin F (**1**) consistent with the ampullosporin series reported by Ritzau et al. in 1997 [21] and Kronen et al. in 2001 [22].

Compound **2** was obtained as a white, amorphous solid. ESI-HRMS studies showed that **2** exhibited the same molecular formula (C_78_H_128_N_18_O_20_) as ampullosporin F (**1**). Positive and negative ion ESI-HRMS^n^ investigations (Table 1; Table S1, Supplementary Materials) revealed that **2** is an isomer of **1**. The only difference between **1** and **2** is the positional exchange of Gln^7^/GluOMe^11^ in **1** by GluOMe^7^/Gln^11^ in **2**. Likewise, NMR data of **2** (Table 3, Figure 4) are closely resemble that of **1**, including the strong correlation between the methoxy singlet at *δ*_H_ 3.54 (*δ*_C_ 51.12) and a carbonyl carbon at *δ*_C_ 172.20 confirming the glutamic acid methyl ester moiety. Thus, the structure of **2** was determined as Ac-Trp^1^-Ala^2^-Aib^3^-Aib^4^-Leu^5^-Aib^6^-GluOMe^7^-Aib^8^-Aib^9^-Aib^10^-Gln^11^-Leu^12^-Aib^13^-Gln^14^-Leuol^15^, and trivially named ampullosporin G (**2**).

The *δ-*methyl ester of glutamic acid is rarely recognized in natural peptaibols, with only five examples out of the over 1450 peptaibiotics reported in the literature so far [5,8,9,10,11,14,38,39,40,41,42,43,44,45,46,47,48,49,50]. The first occurrence was reported for four peptaibols named Trichorzianines (TA) 1896, TA1924, TA1910, and TA1924a isolated from *Trichoderma atroviride* by Panizel et al. in 2013 [13]. The second example was described for Glu(OMe)^18^-alamethicin F50 isolated from *Trichoderma arundinaceum* by Rivera-Chávez et al. in 2017 [8]. Consequently, the isolation and characterization of ampullosporin F (**1**) and G (**2**) represents the third report of glutamic acid methyl ester containing peptaibols isolated from natural sources, and the first occurrence in a *Sepedonium* species.

Compound **3**, isolated as a white, amorphous solid, possesses the molecular formula C_77_H_127_N_19_O_19_ as deduced from ESI-HRMS studies of the [M + H]^+^ ion at *m*/*z* 1622.9628. The structure of **3** was identified as ampullosporin A (**3**, Ac-Trp^1^-Ala^2^-Aib^3^-Aib^4^-Leu^5^-Aib^6^-Gln^7^-Aib^8^-Aib^9^-Aib^10^-Gln^11^-Leu^12^-Aib^13^-Gln^14^-Leuol^15^) based on MS studies in positive mode as well as 1D NMR experiments (Figures S29–S30, Table S2, Supplementary Materials).

Compound **4** was obtained as a white, amorphous solid. On the basis of ESI-HRMS studies, the molecular formula C_31_H_51_N_5_O_6_ was determined from the [M + H]^+^ ion’s signal at *m*/*z* 590.3892. Mass fragmentation analyses coupled with 1D and 2D NMR investigations (Figures S31–S33, Table S2, Supplementary Materials) established **4** as peptaibolin (**4**, Ac-Leu^1^-Aib^2^-Leu^3^-Aib^4^-pheol^5^), the shortest peptaibol with only five amino acid residues. Both compounds ampullosporin A (**3**) and peptaibolin (**4**) were previously isolated from cultures of *S. ampullosporum* HKI-0053 [21,35].

Compound **5** was obtained as a white, amorphous solid. ESI-HRMS analysis of the signal at *m*/*z* 510.3649 ([M + H]^+^, calcd for C_26_H_48_N_5_O_5_^+^ 510.3650) resulted in the molecular formula C_26_H_47_N_5_O_5_. Deduced from mass fragmentation analyses as well as 1D NMR investigations (Figure S34, Table S2, Supplementary Materials) compound **5** was identified as the cyclic pentapeptide chrysosporide (**5**).

Compound **6** was obtained as an amorphous solid and exhibited the molecular formula C_14_H_15_N_3_O_3_ based on ESI-HRMS analyses of the [M − H]^−^ ion at *m*/*z* 272.1042 (Figure S35, Supplementary Materials). 1D NMR spectroscopic data (Figures S36–S37, Supplementary Materials) of **6** were in accordance with those of c(Trp-Ser) (**6**), which was first reported as a synthetic product in 2006 [51] and later isolated from several fungus like *Oidiodendron truncatum* GW3-13 [52], *Acrostalagmus luteoalbus* SCSIO F457 [53], and *Rheinheimera aquimaris* QSI02 [54].

Compound **7** was obtained as a white, amorphous solid. On the basis of ESI-HRMS studies, the molecular formula C_14_H_15_N_3_O_2_ was deduced from the [M − H]^−^ ion at *m*/*z* 256.1089 (Figure S38, Supplementary Materials), indicating the absence of one oxygen atom in **7** compared to **6**. 1D NMR spectroscopic data (Figures S39 and S40, Supplementary Materials) of **7** were in agreement with those of c(Trp-Ala) (**7**), which was first synthesized in 1998 [55] and later recognized in different fungal sources such as *Eurotium* sp. [56] and *Eurotium chevalieri* MUT 2316 [57]. To the best of our knowledge, this is the first detection of c(Trp-Ser) (**6**) and c(Trp-Ala) (**7**) in a *Sepedonium* species.

### 2.2. In Situ Chemical Analysis

Because ampullosporin F (**1**) and G (**2**) were isolated as minor compounds with similar structures as ampullosporin A (**3**), the dominant component of *S. ampullosporum*, a question may arise with regard to the authenticity of these new compounds **1** and **2**. Are the isolated ampullosporins **1** and **2** biosynthesized by fungus itself or are they artefacts formed during extraction, fractionation and/or the purification processes using methanol?

In order to answer this question, a crude extract from cultivated *S. ampullosporum* was prepared using ethanol, instead of methanol, and investigated using LC-HRMS screening approach. From an enriched fraction of the ethanol crude extract of *S. ampullosporum*, the ampullosporin F (**1**) and G (**2**) as well as ampullosporin A (**3**) could be unambiguously detected by their characteristic mass (Figure S43, Supplementary Materials). Therefore, ampullosporin F (**1**) and G (**2**) are native constituents of *S. ampullosporum* Damon strain KSH 534.

### 2.3. Solid Phase Synthesis and Absolute Configuration of Ampullosporin F *(**1**)* and G *(**2**)*

**Scheme 1:**
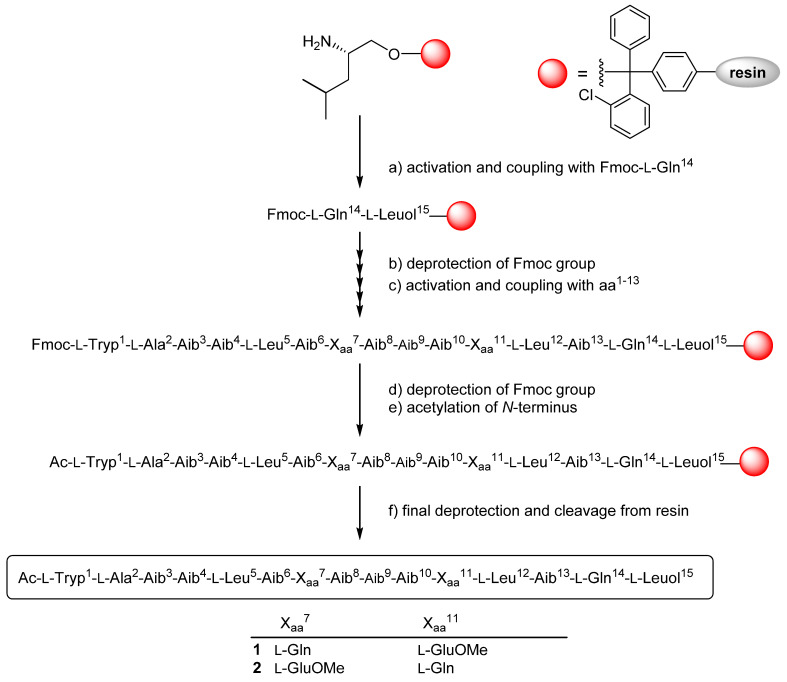
Solid-phase peptide synthesis of ampullosporin F (**1**) and G **(2**).

The absolute configuration of **1** and **2** was established based on solid-phase peptide synthesis using Fmoc protected, L-configured amino acids (except the achiral Aib) and tetramethylfluoroform-amidinium hexafluorophosphate (TFFH) as a coupling reagent (Scheme 1).

The combined manual and automated synthesis was carried out on L-Leucinol 2-chlorotrityl polystyrene resin. The first four amino acids residues were incorporated by automated synthesis using the standard PyBOP (benzotriazol-1-yloxy-tripyrrolidinophosphonium hexafluorophosphate) strategy, while the rest of the synthesis was performed manually using TFFH or HATU (1-[bis(dimethylamino)methylene]-1H-1,2,3-triazolo[4,5-b]pyridinium 3-oxide hexafluorophosphate) activations. *N*-terminal acetylation was achieved after complete peptide assembly by treating the resin with Ac_2_O, followed by solid-phase cleavage and global deprotection using TFA. The synthesized peptaibols **1** and **2** were purified by size exclusion column chromatography using Sephadex LH20 in combination with preparative HPLC. The ESI-HRMS^n^, 1D NMR, and CD spectra of the synthetic peptaibols **1** and **2** were consistent with those of the natural ampullosporin F (**1**) and G (**2**) (Figures S17–S28, Supplementary Materials). Consequently, all chiral amino acids naturally present in **1** and **2** possess the L-configuration, which is typical for the particular class of ampullosporins [21,35].

### 2.4. Evaluation of Antifungal Activities

Isolated peptaibols **1**–**3** were examined for their antifungal activities against plant pathogenic ascomycetous fungi *Botrytis cinerea* (grey mold pathogen on many crops, e.g., strawberries and wine grapes) and *Septoria tritici* (causes septoria leaf blotch of wheat) as well as the oomycete *Phytophthora infestans* (causal agent of the late blight disease on potato and tomato) using a 96-well microtiter plate assay. The commercially available fungicides epoxiconazole and terbinafine were used as positive controls.

All three compounds **1**–**3** showed strong activity against *B. cinerea* and only slightly lower inhibitory effects against *P. infestans*, but were inactive against *S. tritici*. Interestingly, the growth-inhibitory effects against both *B. cinerea* and *P. infestans* increased with the presence of GluOMe in ampullosporin F (**1**) and G (**2**), compared to the ampullosporin A (**3**) (Table 4, Figure S41, Supplementary Materials).

### 2.5. Evaluation of Anticancer Activities

**Figure 5:**
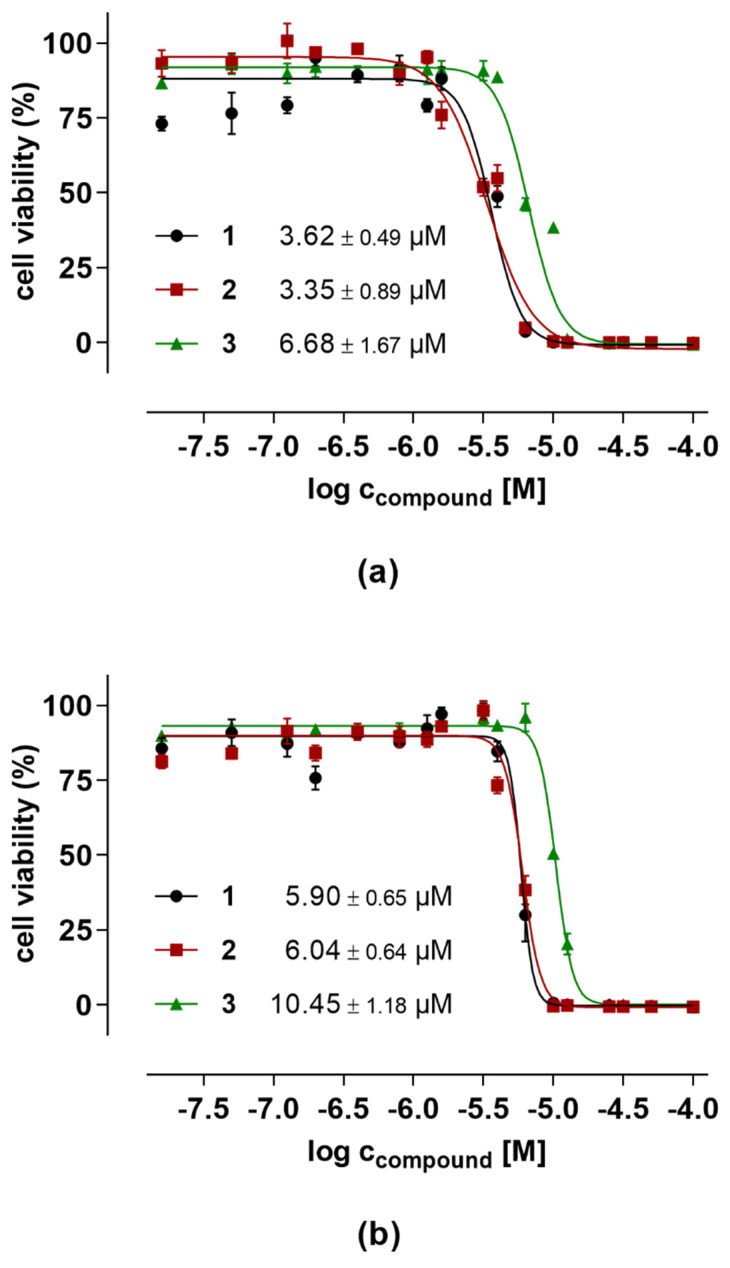
Cell viability of (**a**): prostate cancer PC-3 cells, and (**b**): colon cancer HT-29 cells treated for 48 h with the peptaibols **1** (●), **2** (■), and **3** (▲), respectively, as determined by resazurin-based fluorimetric assay. Data represent biological quadruplicates (*n* = 4), each comprising technical triplicates.

The peptaibols **1**–**3** were tested for their effects on the viability of two different human cancer cell lines, namely prostate PC-3 adenocarcinoma cells and colorectal HT-29 adenocarcinoma cells. The cell viability and cytotoxicity assay was conducted by using resazurin and fluorometric read-out after 48 h cell treatment. The saponin digitonin (100 µM), a very potent permeabilizer of cell membranes, was used as positive control compromising the cells to yield 0% cell viability after 48 h. As negative control, representing 100% cell viability for data normalization, medium with 0.5% (*v*/*v*) DMSO supplementation (highest final DMSO concentration in test item samples) was measured. The peptaibols **1**–**3** were tested with concentrations in the range of 0.195–100 µM (factor 2 dilutions) in order to determine IC_50_ values that have been calculated to be ~3–6 µM for both novel ampullosporins F (**1**) and G (**2**), with twofold higher activity in prostate PC-3 cancer cells compared to the colorectal cancer cells HT-29. Furthermore, in both cancer cell lines, **1** and **2** were found with twofold lower IC_50_s compared to the already known ampullosporin A (**3**) (see Figure 5), indicating that the GluOMe modifications at the amino acid positions 7 and 11, respectively, enhance not only the antifungal but also the anticancer activity of the ampullosporins. Summarized results are shown in Table 4.

### 2.6. In Silico Molecular Docking

In 2009, Berek et al. investigated the neuroleptic-like activity of ampullosporin A (**3**) in mice. Thereby, they described a complete suppression of the effects of the *N*-methyl-D-aspartate (NMDA) receptor antagonist MK-801, and alteration of the activity of those glutamate receptors [23], indicating NMDA receptors as potential biological target molecules of ampullosporin A (**3**). Recently, Lu et al. (2017) investigated the interplay of MK-801 and NMDA receptor in more detail using cryo-EM structural analyses, explaining the allosteric antagonistic action of MK-801 (pdb-code 5UOW) [58]. Based on those results, we decided to proof the hypothesis of NMDA receptor binding of our ampullosporins (**1**–**3**) by using a chemoinformatic molecular docking approach based on two protein databank entries (5UOW and 6IRA). While 5UOW includes the MK-801 inhibitor but reflects the situation at nonhuman NMDA receptor proteins, 6IRA comprises the human GluN1/GluN2A ligand binding domain as the relevant part of the human NMDA receptor [59].

**Figure 6:**
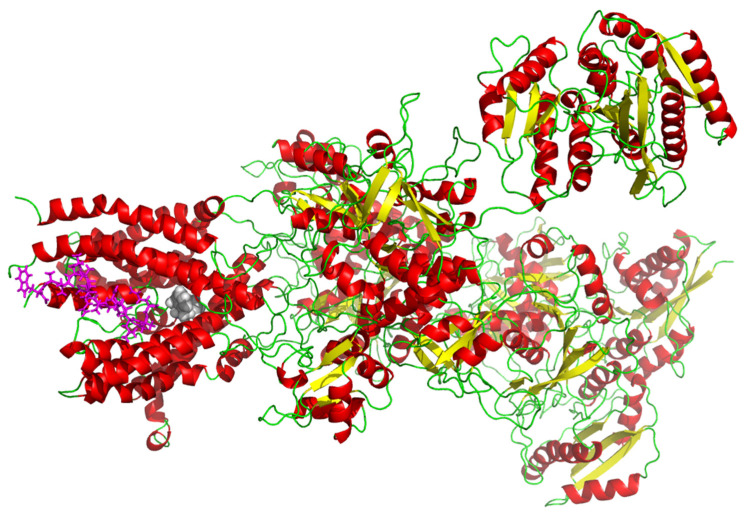
The complete structure of the nonhuman triheteromeric NMDA receptor GluN1/GluN2A/GluN2B (based on protein databank entry 5UOW) with docked ampullosporin A (**3**) (magenta atoms in pink, left site). The position of a proposed allosteric binding site is given by the cryo-EM structure-based position of the inhibitor MK-801 (drawn in grey) in structure of 5UOW.

Based on the protein databank entry 5UOW, a putative binding site on the triheteromeric NMDA receptor GluN1/GluN2A/GluN2B was indicated by the cryo-EM-based localization of the inhibitor MK-801 in structure 5UOW (Figure 6). Moreover, our in silico docking approach based on 5UOW highlighted a very good docking of **3** in very close proximity to MK-801, as also shown in Figure 6. The indicated binding site is located in a hydrophobic cleft that is formed by several helices of both NMDA receptor subunits GluN1 and GluN2A. This theoretical co-localization of ampullosporin A (**3**) and the allosteric NMDA receptor antagonist MK-801 could explain the complete suppression of MK-801 effects caused by the peptaibol (**3**).

**Figure 7:**
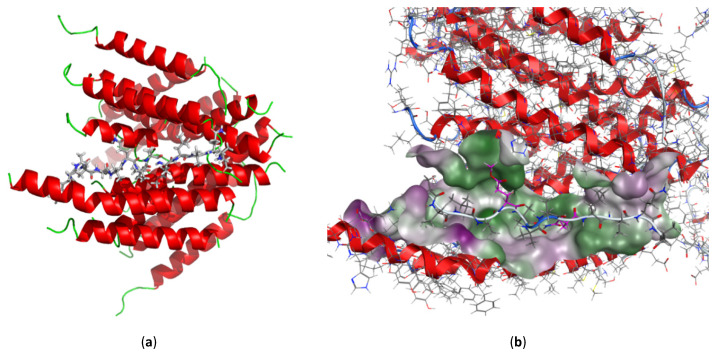
(**a**) Ampullosporins (**1**–**3**) dock with best docking scores into cleft formed by several helices of human NMDA receptor subunits GluN1 and GluN2A. (**b**) The hydrophobic potential blot—hydrophobic (in green)/hydrophilic (in lilac)—indicates the primarily hydrophobic character of the binding site.

In addition, compounds **1**–**3** were tested for their antiproliferative effects in PC-3 prostate and HT-29 colorectal human cancer cells, which, to some extent, express the NMDA receptor proteins GluN1 and GluN2A (according to mRNA expression data; analyzed by using the Genevestigator software, data base HS_mRNASeq_HUMAN_GL-1; data not shown). Therefore, we also investigated the ampullosporins’ binding in silico based on protein databank entry 6IRA representing the human GluN1/GluN2A NMDA receptor complex that, however, do not include MK-801. Indeed, also in our 6IRA-based in silico model the peptaibols **1**–**3** were docked with best scores at the same position in the hydrophobic cleft between GluN1 and GluN2A (Figure 7).

Our in silico results, coupled with the observations by Berek et al. [23], led to the hypothesis that this hydrophobic cleft at the interphase between GluN1 and GluN2A could be the binding site for the very hydrophobic peptaibols **1**–**3**, causing their biological effects. To shed more light on the molecular ampullosporins binding mode and especially to investigate consequences of the sequence modifications in the novel ampullosporin F (**1**) and G (**2**), further docking studies were performed to validate this hydrophobic cleft as a potential binding site of compounds **1**–**3**.

**Figure 8:**
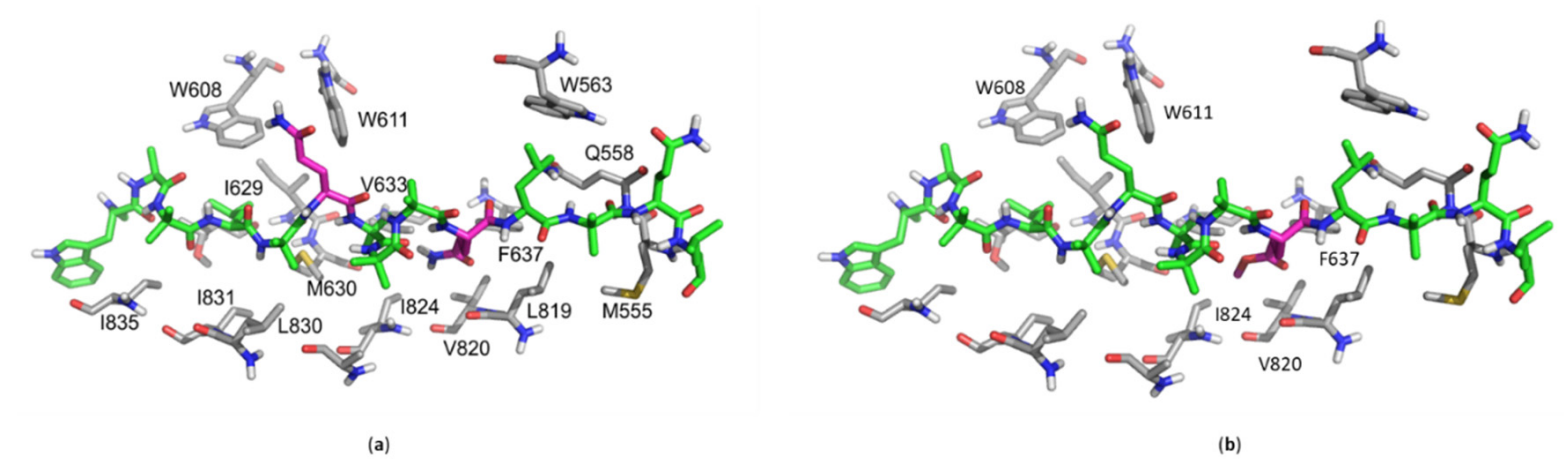
Docking arrangements of (**a**) ampullosporin A (**3**), and (**b**) ampullosporin F (**1**) in the proposed binding site in the hydrophobic cleft between NMDA receptor subunits GluN1 and GluN2A, as modeled based on protein databank entry 6IRA.

As illustrated in Figure 8 and Figure S42 (Supplementary Materials), all compounds **1**–**3** fit nicely into the described hydrophobic cleft and seem to be stabilized by numerous hydrophobic interactions to hydrophobic receptor side chains. Accordingly, for ampullosporin A (**3**) an interaction energy of −92.0 kcal/mol was calculated. However, replacement of each of Gln residues (Gln^7^ and Gln^11^) in ampullosporin A (**3**) by GluOMe resulted in additional hydrophobic interactions of ampullosporin F (**1**; GluOMe^11^) and ampullosporin G (**2**; GluOMe^7^) with hydrophobic receptor residues. In case of **1**, enhanced hydrophobic interactions to the receptor residues V820, I824 and F637 elevate the calculated interaction energy to be −94.5 kcal/mol. In case of **2**, the molecular docking indicates additional hydrophobic interactions of its GluOMe^7^ modification with the hydrophobic receptor residues W608 and W611. The calculated interaction energy (−92.9 kcal/mol) for **2** is slightly enhanced compared to **3**. Based on these calculated interaction energies, the binding of both ampullosporin F (**1**) and G (**2**) should outperform the binding of ampullosporin A (**3**).

Interestingly, the determined antiproliferative activities of compounds **1**–**3** (Figure 5) correlate very well with the proposed enhanced binding of both novel ampullosporins (**1** and **2**). The antiproliferative IC_50_ values in the anticancer assay were detected to be by twofold better for ampullosporin F (**1**) and G (**2**) than for ampullosporin A (**3**).

Whether or not the NMDA receptor is really the relevant molecular target explaining the observed antiproliferative effects of the ampullosporins on human cancer cells, further investigations are necessary. However, considering the well-published neuroleptic-like activities of ampullosporin A (**3**) [21,23,60] that can be easily attributed to NMDA receptor pathways, it would be of high interest to test our novel ampullosporins F (**1**) and G (**2**), with presumed improved NMDA receptor binding for their neuroleptic activity, e.g., in vivo in mice.

## 3. Materials and Methods

### 3.1. General Experimental Procedures

Column chromatography was carried out on Sephadex LH 20 (Fluka, Steinheim, Germany), while analytical TLC was performed on precoated silica gel F254 aluminum sheets (Merck, Darmstadt, Germany). Peptaibols were detected on TLC plates using ninhydrin reagent as described in the literature [17]. Diaion HP 20 was purchased from Supelco (Bellefonte, PA, USA). UV spectra were recorded on a Jasco V-770 UV-Vis/NIR spectrophotometer (Jasco, Pfungstadt, Germany), meanwhile CD spectra were obtained from a Jasco J-815 CD spectropolarimeter (Jasco, Pfungstadt, Germany). The specific rotation was measured with a Jasco P-2000 digital polarimeter (Jasco, Pfungstadt, Germany).

NMR spectra were obtained from an Agilent DD2-400 and an Agilent VNMRS 600 system (Varian, Palo Alto, CA, USA) using a 5-mm inverse detection cryoprobe. Compounds were dissolved in DMSO-*d_6_* (99.96% D for **1** and **2**, 99.80% D for **3**–**7**). The spectra were recorded at 399.82/599.83 MHz (^1^H) and 100.54/150.84 (^13^C), respectively. 2D NMR spectra were recorded using standard CHEMPACK 8.1 pulse sequences (s2pul, ^1^H,^1^H gDQCOSY, ^1^H,^1^H zTOCSY, ^1^H,^1^H ROESYAD, ^1^H,^13^C and ^1^H,^15^N gHSQCAD, ^1^H,^13^C gHMBCAD) implemented in Varian VNMRJ 4.2 spectrometer software (Varian, Palo Alto, CA, USA). The mixing time for the TOCSY experiments was set to 80 ms, for the ROESY experiments was set to 300 ms. The HMBC experiment was optimized for a long-range coupling of 8 Hz. ^1^H and ^13^C chemical shifts were referenced to internal DMSO-*d_6_* (*δ*_H_ 2.51 ppm and *δ*_C_ 39.5 ppm), whereas ^15^N chemical shifts were given relative to liquid NH_3_ (*δ*_N_ 0 ppm).

The solid phase peptide synthesis was partly carried out on a ResPep SL peptide synthesizer (Intavis Bioanalytical Instruments, Köln, Germany). The L-configured Fmoc-amino acids Fmoc-Trp(Boc)-OH, Fmoc-Ala-OH, Fmoc-Leu-OH, Fmoc-Gln(Trt)-OH and Fmoc-Glu(OMe)-OH were purchased from Carbolution Chemicals (Ingbert, Germany), while L-Leucinol 2-chlorotrityl polystyrene resin was obtained from Iris Biotech GmbH (Marktredwitz, Germany). TFFH was supplied Carbolution Chemicals (Ingbert, Germany). Piperidine, Ac_2_O, DIPEA, and DMF were purchased from Sigma-Aldrich (Steinheim, Germany).

The high-resolution mass spectra in positive and negative modes were obtained from an Orbitrap Elite mass spectrometer (Thermofisher Scientific, Bremen, Germany) equipped with an ESI electrospray ion source (spray voltage 4.0 kV; capillary temperature 275 °C, source heater temperature 40 °C; FTMS resolution 60.000). Nitrogen was used as sheath gas. The sample solutions were introduced continuously via a 500 μL Hamilton syringe pump with a flow rate of 5 μL/min. The instrument was externally calibrated by the Pierce^®^ LTQ Velos ESI positive ion calibration solution (product number 88323) and Pierce^®^ ESI negative ion calibration solution (product number 88324) from Thermofisher Scientific (Rockford, IL, USA). The data were evaluated by the Xcalibur software 2.7 SP1 Thermofisher Scientific, Waltham, MA, USA). The collision induced dissociation (CID) MS^n^ measurements were performed using the relative collision energies given in Table S1 (Supplementary Materials).

The preparative HPLC was performed on a Shimadzu prominence system (Kyoto, Japan) which consists of a CBM-20A communications bus module, a SPD-M20A diode array detector, a FRC-10A fraction collector, a DGU-20A5R degassing unit, a LC-20AT liquid chromatograph, and a SIL-20A HT auto sampler, using either column 1 (ODS-A, 5 µm, 120 Å, 150 × 20 mm I.D; YMC, Devens, MA, USA) or column 2 (ODS-A, 5 µm, 120 Å, 150 × 10 mm I.D; YMC, Devens, MA, USA). The mobile phases were H_2_O (A) and CH_3_CN (B), with 0.1% formic acid contained in both solvents, using a gradient system.

### 3.2. Fungal Strain and Cultivation

The fungal strain *Sepedonium ampullosporum* Damon KSH 534 was isolated in August 1999, from *Boletus calopus* in Crista Acri near Cosenza, Italy (leg./det. C. Lavorato). A voucher specimen is deposited at the herbarium of the University Regensburg. The fungal culture of *S. ampullosporum* strain KSH 534 was stored on malt peptone agar (MPA) plates and transferred periodically. The upscaled semi-solid cultures, used for isolation, were grown in 31 Erlenmeyer flasks (size 1 L) each containing 1.5 g of cotton wool and 250 mL of malt peptone medium (2.5 g malt and 0.625 g peptone in 250 mL deionized water), resulting in a total volume of 7.75 L. Each culture flask was inoculated with a 10 × 10 mm agar plug of colonized fungus and incubated for 14 days at room temperature without agitation.

### 3.3. Extraction and Isolation

The mycelia were separated from the culture broth by vacuum filtration, frozen with liquid nitrogen and subsequently extracted with EtOAc (2 × 2 L) and MeOH (2 × 2 L) to yield two crude extracts (EtOAc, 2.20 g and MeOH, 8.98 g, respectively). Meanwhile, activated Diaion HP 20 (50 g) was added to the culture broth and agitated for 12 h at room temperature. Diaion HP 20 was then removed by vacuum filtration, washed with H_2_O, eluted with MeOH to give a yellow solution, which was then evaporated *in vacuo* to dryness. This dryness (3.44 g) from culture broth was combined with the EtOAc extract from mycelia according to their LC-MS profiles. The resulting residue (5.64 g) was chromatographed on a Sephadex LH 20 column, using aq. MeOH 60% as eluent to afford 140 fractions (8 mL each). Based on ESI-MS spectra, fractions 26–38 were combined (1.91 g), which were first separated by size exclusion column chromatography (using Sephadex LH 20, eluent: MeOH), then subjected to preparative HPLC using column 1 at a flow rate of 4.5 mL/min (0–30 min, 30–80% B; 31–50 min, 80–100% B) to afford **3** (ampullosporin A, t*_R_* = 34.0 min, 100.9 mg), **1** (ampullosporin F, t*_R_* = 37.5 min, 4.7 mg), and **2** (ampullosporin G, t*_R_* = 40.5 min, 3.8 mg). In addition, the combination of fractions 39–48 (311.7 mg) was purified by semipreparative HPLC using column 2 at a flow rate of 2.2 mL/min (0–12 min, 30–100% B; 12–17 min, 100% B) to afford **4** (peptaibolin, t*_R_* = 12.7 min, 1.4 mg) and **5** (chrysosporide, t*_R_* = 14.5 min, 0.3 mg). Similarly, fractions 61–74 (85.4 mg) were combined and finally purified by semipreparative HPLC using column 2 at a flow rate of 2.2 mL/min (0–20 min, 10–100% B) to afford **6** (c(Trp-Ser), t*_R_* = 10.6 min, 8.1 mg) and **7** (c(Trp-Ala), t*_R_* = 11.2 min, 7.3 mg).

### 3.4. Sample Preparation for LC-MS Screening

For the preparation of the enriched fraction for the LC-HRMS screening, one stored deep frozen agar plate cultures of *S. ampullosporum* Damon strain KSH534 was crushed in small pieces and extracted with EtOH 96% (2 × 250 mL) in an ultrasonic bath at room temperature. The resulted yellow solution was evaporated *in vacuo* to dryness. The dried crude extract was redissolved in EtOH 96%/H_2_O (1:2, *v*/*v*) to a concentration of 50 mg/mL. The resulting solution was separated on SPE cartridges Chromabond^®^ C18 (loading 200 mg/3 mL, particle size 45 µm; Macherey-Nagel, Düren, Germany), targeted peptaibols **1**–**3** were eluted with EtOH 96%. After evaporation to dryness *in vacuo*, the enriched fraction was redissolved in CH_3_CN and submitted to LC-HRMS.

### 3.5. Solid-Phase Peptide Synthesis

Compounds **1** and **2** were synthesized by combining manual and automated synthesis. The protocol was based on a Fmoc/*t*-butyl strategy in a 0.1 mmol scale starting from L-Leucinol 2-chlorotrityl resin (200–400 mesh, loading 0.67 mmol/g resin). The first four amino acid residues were incorporated by automated synthesis using the standard method based in PyBOP/NMM activation. The rest of the synthesis was performed manually using 4 equiv. of the amino acids and DMF as solvent. For all the Aib residues, the coupling cycle protocol was based on activation with TFFH (4 equiv.) and NMM (*N*-methylmorpholine, 4 equiv.) for 12 min and coupling time of 120 min. The rest of the amino acids were coupled using activation with HATU (4 equiv.) and NMM (4 equiv.) for 5 min and a coupling time of 120 min. Fmoc removals were carried out using a solution of 20% piperidine in DMF for two cycles of 10 min. *N*-terminal acetylation was performed after complete peptide assembly by treating the resin with Ac_2_O (10 equiv) and DIPEA (10 equiv) in DMF for 30 min. Solid-phase cleavage and global deprotection was achieved by treating the resin with 5 mL of TFA/H_2_O/TIPS (95:2.5:2.5, *v*/*v*/*v*) for 120 min. The cleavage mixture was concentrated under reduced pressure, suspended in a mixture of CH_3_CN 50% in water and lyophilized.

For the peptide **1**, the crude peptide mixture (183.8 mg) obtained after lyophilizing was redissolved in MeOH, and subjected to column chromatography (360 × 30 mm) using Sephadex LH20, eluting with MeOH to afford 30 fractions (8mL each). Fractions 6 and 7 were combined (78.6 mg) and purified by preparative HPLC using column 1 (0–2 min, 30–70% B; 3–15 min, 70–100% B; 10 mL/min) to give **1** in 7.0% total yield (t*_R_* = 15.6 min, 11.5 mg).

For the peptide **2**, the crude peptide mixture (201.7 mg) obtained after lyophilizing was redissolved in MeOH, and separated by column chromatography (360 × 30 mm) on Sephadex LH20, using MeOH as eluent to give 30 fractions (8mL each). Fractions 1–5 were combined (95.6 mg) and purified by preparative HPLC using column 1 (0–2 min, 30–80% B; 3–15 min, 80–100% B; 10 mL/min) to give **2** in 7.7% total yield (t*_R_* = 16.2 min, 12.6 mg).

Natural ampullosporin F (**1**): white, amorphous solid; TLC R_f_ 0.34 (*n*-BuOH/AcOH/H_2_O 4:1:1); ${\left[\alpha \right]}_{D}^{23}$-15.6 (*c* 0.100, MeOH); CD (MeOH) [*θ*]_208_-120119, [*θ*]_224_-103881 deg cm^2^ × dmol^−1^; UV (MeOH) λmax (log *ε*) 290 (23.56) nm; ^1^H NMR and ^13^C NMR see Table 2; ESI-HRMS *m*/*z* 819.4849 ([M + 2H]^2+^, calcd for C_78_H_130_N_18_O_20_^2+^ 819.4849); ESI-HRMS^n^ see Table 1, and Table S1 (Supplementary Materials).

Synthetic ampullosporin F (**1**): white, amorphous solid; CD (MeOH) [*θ*]_208_-232819, [*θ*]_224_-208346 deg cm^2^ × dmol^−1^; ^1^H NMR and ^13^C NMR in accordance with data of natural **1**; ESI-HRMS *m*/*z* 819.4852 ([M + 2H]^2+^, calcd for C_78_H_130_N_18_O_20_^2+^ 819.4849); ESI-HRMS^n^ in agreement with natural **1**.

Natural ampullosporin G (**2**): white, amorphous solid; TLC R_f_ 0.44 (*n*-BuOH/AcOH/H_2_O 4:1:1); ${\left[\alpha \right]}_{D}^{23}$-17.1 (*c* 0.080, MeOH); CD (MeOH) [*θ*]_208_-150953, [*θ*]_223_-125977 deg cm^2^ × dmol^−1^; UV (MeOH) λmax (log *ε*) 281 (1.88), 290 (1.81) nm; ^1^H NMR and ^13^C NMR see Table 3; ESI-HRMS *m*/*z* 819.4860 ([M + 2H]^2+^, calcd for C_78_H_130_N_18_O_20_^2+^ 819.4849); ESI-HRMS^n^ see Table 1, and Table S1 (Supplementary Materials).

Synthetic ampullosporin G (**2**): white, amorphous solid; CD (MeOH) [*θ*]_207_-246393, [*θ*]_222_-205061 deg cm^2^ × dmol^−1^; ^1^H NMR and ^13^C NMR in accordance with data of natural **2**; ESI-HRMS *m*/*z* 819.4852 ([M + 2H]^2+^, calcd for C_78_H_130_N_18_O_20_^2+^ 819.4849); ESI-HRMS^n^ in agreement with natural **2**.

Natural ampullosporin A (**3**): white, amorphous solid; TLC R_f_ 0.26 (*n*-BuOH/AcOH/H_2_O 4:1:1); ^1^H NMR and ^13^C in agreement with data of Ritzau et al. (1997) [21]; ESI-HRMS *m*/*z* 811.9851 ([M + 2H]^2+^, calcd for C_77_H_129_N_19_O_19_^2+^ 811.9851); ESI-HRMS^n^ see Table S2, Supplementary Materials.

Natural peptaibolin (**4**): white, amorphous solid; TLC R_f_ 0.88 (*n*-BuOH/AcOH/H_2_O 4:1:1); ^1^H NMR and ^13^C in agreement with data of Hulsmann et al. (1998) [35]; ESI-HRMS *m*/*z* 590.3892 ([M + H]^+^, calcd for C_31_H_52_N_5_O_6_^+^ 590.3912); ESI-HRMS^n^ see Table S2, Supplementary Materials.

Natural chrysosporide (**5**): white, amorphous solid; TLC R_f_ 0.68 (*n*-BuOH/AcOH/H_2_O 4:1:1); ^1^H NMR in agreement with data of Mitova et al. (2006) [32]; ESI-HRMS *m*/*z* 510.3649 ([M + H]^+^, calcd for C_26_H_48_N_5_O_5_^+^ 510.3650); ESI-HRMS^n^ see Table S2, Supplementary Materials.

Natural c(Trp-Ser) (**6**): white, amorphous solid; TLC R_f_ 0.66 (*n*-BuOH/AcOH/H_2_O 4:1:1); ^1^H NMR and ^13^C in agreement with data of Tullberg et al. (2006) [51]; ESI-HRMS *m*/*z* 272.1042 ([M − H]^−^, calcd for C_14_H_14_N_3_O_3_^−^ 272.1041).

Natural c(Trp-Ala) (**7**): white, amorphous solid; TLC R_f_ 0.76 (*n*-BuOH/AcOH/H_2_O 4:1:1); ^1^H NMR and ^13^C in agreement with data of Zhao et al. (2018) [56]; ESI-HRMS *m*/*z* 256.1089 ([M − H]^−^, calcd for C_14_H_14_N_3_O_2_^−^ 256.1092).

### 3.6. Antifungal Assay

Compounds **1**–**3** were tested in a 96-well microtiter plate assay against *Botrytis cinerea* Pers., *Septoria tritici* Desm., and *Phytophthora infestans* (Mont.) de Bary as described earlier [19]. The experiment was carried out in triplicates (*n* = 3), each comprising technical triplicates. For data analyses, GraphPad Prism version 8.0.2 (GraphPad Software, San Diego, CA, USA), SigmaPlot 14.0 (Systat Software, San Jose, CA, USA) and Microsoft Excel 2013 (Microsoft, Redmond, WA, USA) were used.

### 3.7. In Vitro Cell Proliferation Assay—Anticancer Activity

The prostate cancer cell line PC-3 (ATCC, Manassas, VA, USA) and the colon cancer cell line HT-29 (ATCC, Manassas, VA, USA) were cultured in RPMI 1640 medium supplemented with 2 mM L-glutamine and 10% heat-inactivated FCS. The cells were routinely grown in a humidified atmosphere with 5% CO_2_ at 37 °C to reach subconfluency (~70–80%) prior to subsequent usage or subculturing. The adherent cells were rinsed with PBS and detached by using trypsin/EDTA (0.05% in PBS) prior to cell passaging and seeding. RPMI 1640 basal medium, FCS, L-glutamine, PBS and trypsin/EDTA for cell culturing were purchased from Capricorn Scientific GmbH (Ebsdorfergrund, Germany). The culture flasks, multi-well plates and further cell culture plastics were purchased from TPP (Trasadingen, Switzerland) and Greiner Bio-One GmbH (Frickenhausen, Germany), respectively.

Antiproliferative and cytotoxic effects, respectively, of the compounds **1**–**3** were investigated by performing a fluorimetric resazurin-based cell viability assay (Sigma-Aldrich, Taufkirchen, Germany). For that purpose, prostate PC-3 and colorectal HT-29 cancer cells were seeded in low densities into 96-well plates (3000–6000 cells per well; seeding confluency ~10%) and were allowed to adhere for 24 h. Subsequently, the cells were treated for 48 h with compound concentrations up to 100 µM. For control measurements, cells were treated in parallel with 0.5% DMSO (negative control, representing the final DMSO content of the highest concentrated test compound concentration) and 100 µM digitonin (positive control, for data normalization set to 0% cell viability), both in standard growth medium. As soon as the 48-h incubation was finished, the incubation medium was discarded, and cell were rinsed once with PBS. Resazurin solution in RPMI 1640 without phenol red and other supplements was prepared freshly prior to use, and added to the cells in a final resazurin concentration of 50 µM. Subsequently, the cells were incubated under standard growth conditions for further 2 h. Finally, the conversion of resazurin to resorufin by viable, metabolically active cells was measured with 540 nm excitation and 590 nm emission settings by using a SpectraMax M5 multiwell plate reader (Molecular Devices, San Jose, CA, USA). Data were determined in biological quatruplicates, each with technical triplicates. For data analyses GraphPad Prism version 8.0.2 (GraphPad Software, San Diego, CA, USA) and Microsoft Excel 2013 (Microsoft, Redmond, WA, USA) were used.

### 3.8. Computational Details

The X-ray structures of the human GluN1/GluN2A NMDA receptor in the glutamate/glycine-bound state at pH 7.8 (pdb-code 6IRA) [59] and nonhuman (frog and mouse) triheteromeric NMDA receptor GluN1/GluN2A/GluN2B in complex with glycine, glutamate, the uncompetitive NMDA receptor antagonist MK-801 and a GluN2B-specific Fab, at pH 6.5 (pdb-code 5UOW) [58] were downloaded from the protein databank [61] and used for in silico docking analyses of compounds **1**–**3**.

All theoretical investigations were performed using the molecular modeling software package MOE (molecular operating environment) [62]. The X-ray structure was prepared for docking studies by adding the missing hydrogen atoms using the 3D-protonate module implemented in MOE.

The putative active site of the enzyme is indicated by the co-crystallized inhibitor MK-801 (see Figure 6, left site). By closer inspection of this site, an almost complete hydrophobic cleft formed by several helices could be detected, which led to the hypothesis that this could be a perfect binding site for the very hydrophobic peptaibols due to multiple Aib amino acid containing residues. Therefore, docking studies were performed defining this as a binding site. For each of the three peptaibols, ampullosporin A (**3**), F (**1**), and G (**2**), 30 poses were generated using the triangle matcher for fast placement, the London dG as fitness function with subsequent induced fit relaxation of the binding site. The best scored docking poses are displayed and discussed.

## 4. Conclusions

In conclusion, the present study represents the chemical investigation of semi-solid culture of *Sepedonium ampullosporum* Damon strain KSH 534. There are seven constituents including two new 15-residue linear peptaibols, named ampullosporin F (**1**) and G (**2**), as well as two known linear peptaibols, ampullosporin A (**3**) and peptaibolin (**4**), together with three previously described cyclic peptides, chrysosporide (**5**), c(Trp-Ser) (**6**), and c(Trp-Ala) (**7**). The authenticity of peptaibols **1** and **2** was approved by using LC-HRMS approach. Additionally, the total synthesis of **1** and **2** was performed on solid-phase synthesis establishing the L-configuration of all chiral amino acids. Furthermore, peptaibols **1**–**3** showed significant anti-phytopathogenic activity against *B. cinerea* and *P. infestans*, but no activity against *S. tritici*. Moreover, compounds **1**–**3** exhibited strong anticancer activities against human prostate (PC-3) and colorectal (HT-29) cancer cells. Interestingly, for both antifungal and anticancer assays, the activities of ampullosporin F (**1**) and G (**2**) were found to be twofold higher than the structurally similar ampullosporin A (**3**), demonstrating the effect of a GluOMe moiety on biological activity. Our molecular docking data on NMDA receptors suggests the better hydrophobic interaction of **1** and **2** than **3** with the hydrophobic cleft of the receptor, which might lead to the higher inhibitory effects of the new compounds **1** and **2** compared to **3**.

## Figures and Tables

**Table 1:** Diagnostic fragment ions [*m/z*] of ampullosporin F (**1**) and ampullosporin G (**2**) from ESI-HRMS^n^ experiments in positive and negative ion modes.

	1	2		1	2
t_R_ (min)	11.62	11.54	y_11_^+^	1168.7280	1168.7294
[M + 2H]^2+^	819.4849	819.4864	y_12_^+^	*n.d.*	*n.d.*
[M + H]^+^	1637.9608	1637.9646	y_13_^+^	*n.d.*	*n.d.*
b_1_^+^	229.0977	229.0978	y_14_^+^	*n.d.*	*n.d.*
b_2_^+^	300.1361	300.1345	y_9_^+^	970.5923	970.5915
b_3_^+^	385.1870	385.1874	y_9_^+^-aa^15^	853.4770	853.4781
b_4_^+^	470.2393	470.2400	y_9_^+^-aa^14–15^	725.4192	725.4180
b_5_^+^	583.3235	583.3235	y_9_^+^-aa^13–15^	640.3662	640.3654
b_6_^+^	668.3765	668.3763	y_9_^+^-aa^12–15^	527.2811	527.2847
b_7_^+^	796.4326	811.4340	y_9_^+^-aa^11–15^	*n.d.*	399.2281
b_8_^+^	881.4879	896.4871	y_9_^+^-aa^10–15^	*n.d.*	*n.d.*
b_9_^+^	966.5402	981.5396	y_9_^+^-aa^9–15^	*n.d.*	*n.d.*
b_10_^+^	1051.5927	1066.5923	y_9_^+^-aa^8–15^	*n.d.*	*n.d.*
b_11_^+^	1194.6510	1194.6440	[M − H]^−^	1635.9458	1635.9453
b_12_^+^	1307.7346	1307.7357	y_2_^−^	*n.d.*	*n.d.*
b_13_^+^	1392.7877	1392.7875	y_3_^−^	*n.d.*	*n.d.*
b_14_^+^	*n.d.*	*n.d.*	y_4_^−^	*n.d.*	*n.d.*
y_1_^+^	*n.d.*	*n.d.*	y_5_^−^	*n.d.*	570.3615
y_2_^+^	*n.d.*	246.1820	y_6_^−^	*n.d.*	655.4136
y_3_^+^	*n.d.*	*n.d.*	y_7_^−^	*n.d.*	740.4655
y_4_^+^	444.3214	444.3169	y_8_^−^	*n.d.*	825.5215
y_5_^+^	587.3766	572.3767	y_9_^−^	968.5731	968.5756
y_6_^+^	672.4281	657.4289	y_10_^−^	1053.6372	1053.6267
y_7_^+^	757.4816	742.4813	y_11_^−^	1166.7102	1166.7143
y_8_^+^	842.5358	827.5331	y_12_^−^	1251.7668	1251.7572
y_9_^+^	970.5923	970.5915	y_13_^−^	1336.8179	1336.8088
y_10_^+^	1055.6453	1055.6426	y_14_^−^	1407.8525	1407.8543

**Table 2:** NMR data of ampullosporin F (**1**) (600/150 MHz, DMSO-d6, *δ* in ppm).

Pos.	*δ*_H_, Mult. *J* (Hz)	*δ*_C_/*δ*_N_	Pos.	*δ*_H_, Mult. *J* (Hz)	*δ*_C_/*δ*_N_	Pos.	*δ*_H_, Mult. *J* (Hz)	*δ*_C_/*δ*_N_
**Ac**			β	1.77 ^a^; 1.58 ^a^	38.4	C=O		173.0
CH_3_	1.86 s	22.2	γ	1.72 ^a^	23.8	α	3.95 m	54.4
C=O		170.2	δ_1_	0.84 d 6.6	20.8	β	2.07 ^a^; 2.00 ^a^	25.3
**Trp^1^**			δ_2_	0.93 d 6.6	22.0	γ	2.60 m; 2.45 m	29.6
NH	8.38 d 6.5	125.3	**Aib^6^**			C=O		172.5
C=O		173.0	NH	7.87 s	127.9	O-CH_3_	3.55 s	50.8
α	4.40	54.4	C=O		175.5	**Leu^12^**		
β	3.12 dd 14.6/3.6	26.8	α	1.48	55.4	NH	7.42 d 7.3	115.4
	2.98 dd 14.6/9.4		β	1.36 s	22.6	C=O		172.8
1-NH	10.86 br s	131.2	γ	1.48 s	26.1	α	4.08 m	52.5
2	7.22 d 2.4	123.4	**Gln^7^**			β	1.67 ^a^; 1.59 ^a^	38.4
3		109.8	NH	7.41 d 6.2	112.9	γ	1.73 ^a^	23.7
3a		127.1	C=O		173.1	δ_1_	0.86 d 6.6	22.2
4	7.56 d 8.1	117.9	α	3.82 m	55.7	δ_2_	0.83 d 6.6	20.6
5	6.96 t 7.5	117.7	β	2.00 ^a^; 1.91 ^a^	25.8	**Aib^13^**		
6	7.05 ^a^	120.5	γ	2.22 ^a^; 2.07 ^a^	31.0	NH	7.45 s	126.5
7	7.33 d 8.1	111.0	C=O		172.9	C=O		173.4
7a		136.0	N-H_2_	7.12 br s	107.7	α		56.0
**Ala^2^**				6.75 br s		β	1.38 s	23.7
NH	8.52 ^a^	121.9	**Aib^8^**			γ	1.41 s	25.4
C=O		174.0	NH	7.99 s	128.5	**Gln^14^**		
α	4.04 ^a^	50.1	C=O		175.0	NH	7.19 d 7.7	109.2
β	1.27 d 7.4	15.5	α		55.1	C=O		173.1
**Aib^3^**		132.1	β	1.31 s	22.0	α	3.98 m	52.9
NH	8.46 s	174.6	γ	1.43 s	25.3	β	2.05 ^a^; 1.81 ^a^	26.7
C=O		55.4	**Aib^9^**			γ	2.18 ^a^; 2.08 ^a^	31.2
α		25.3	NH	7.96 s	125.2	C=O		173.5
β	1.38 s	22.6	C=O		175.0	N-H_2_	7.13 br s	107.9
γ	1.35 s	22.6	α		55.3		6.73 br s	
**Aib^4^**			β	1.39 s	26.0	**Leuol^15^**		
NH	8.06 s	124.3	γ	1.32 s	22.0	NH	7.06 ^a^	118.8
C=O		176.3	**Aib^10^**			α	3.78 m	48.2
α		55.3	NH	7.57 s	125.1	β	1.35 ^a^	39.4
β	1.38 s	26.2	C=O		175.8	γ	1.66 ^a^	23.5
γ	1.36 s	22.0	α		55.4	δ_1_	0.86 d 6.6	23.2
**Leu^5^**			β	1.39 s	22.3	δ_2_	0.81 d 6.6	21.2
NH	7.71 d 4.1	114.8	γ	1.49 s	26.1	β’	3.30 ^a^; 3.18 m	63.5
C=O		173.7	**GluOMe^11^**			O-H	4.50 t 6.0	
α	3.90 m	54.1	NH	7.76 d 6.6	111.2			

Chemical shifts of quaternary carbons were determined from ^1^H, ^13^C HMBC correlation peaks; ^a^ overlapping signals, chemical shifts were determined from ^1^H, ^15^N or ^1^H, ^13^C HSQC correlation peaks.

**Table 3:** NMR data of ampullosporin G (**2**) (600/150 MHz, DMSO-d6, *δ* in ppm).

Pos.	*δ*_H_, Mult. *J* (Hz)	*δ*_C_/*δ*_N_	Pos.	*δ*_H_, Mult. *J* (Hz)	*δ*_C_/*δ*_N_	Pos.	*δ*_H_, Mult. *J* (Hz)	*δ*_C_/*δ*_N_
**Ac**			β	1.55 ^a^; 1.76 ^a^	38.7	α	3.93 m	55.0
CH_3_	1.85 br s	22.3	γ	1.70 ^a^	24.3	β	1.94 ^a^; 2.00 ^a^	26.3
C=O		170.4	δ_1_	0.833 d 6.6	21.1	γ	2.13 m; 2.34 m	31.5
**Trp^1^**			δ_2_	0.914 d 6.6	22.1	C=O		173.2
NH	8.30 ^a^	125.2	**Aib^6^**			N-H_2_	7.12 br s	107.6
C=O		173.0	NH	7.85 s	127.8		6.70 br s	
α	4.39 m	54.4	C=O		176.0	**Leu^12^**		
β	3.11 dd 14.4/4.2		α		55.6	NH	7.77 d 6.6	115.2
	2.98 dd 14.4/9.6	26.8	β	1.35 s	22.2	C=O		173.2
1-NH	10.84 br s	131.1	γ	1.45 s	26.2	α	4.07 m	52.6
2	7.21 d 2.4	123.5	**GluOMe^7^**			β	1.58 ^a^ & 1.67 ^a^	38.5
3		109.6	NH	7.47 d 6.0	111.8	γ	1.70 ^a^	23.9
3a		126.9	C=O		172.8	δ_1_	0.85 d 6.6	22.3
4	7.55 d 7.8	118.0	α	3.87 m	55.2	δ_2_	0.82 d 6.6	20.7
5	6.95 t-like 7.2	117.9	β	1.96 ^a^; 2.03 ^a^	25.4	**Aib^13^**		
6	7.06 t-like 7.8	120.7	γ	2.49 ^a^; 2.39 m	29.7	NH	7.48 s	126.7
7	7.33 d 8.1	111.1	C=O		172.2	C=O		173.7
7a		135.9	O-CH_3_	3.54 s		α		56.0
**Ala^2^**			**Aib^8^**			β	1.37 s	23.8
NH	8.30 ^a^	121.6	NH	7.97 s	128.7	γ	1.41 s	25.6
C=O		174.0	C=O		175.0	**Gln^14^**		
α	4.01 m	50.3	α		55.4	NH	7.22 d 8.4	109.3
β	1.25 d 7.2	15.7	β	1.34 s	22.4	C=O		170.6
**Aib^3^**			γ	1.42 s	25.4	α	3.98 m	53.0
NH	8.33 s	131.9	**Aib^9^**			β	1.80 ^a^ & 2.05 ^a^	26.6
C=O		174.7	NH	7.87 s	125.0	γ	2.09 ^a^ & 2.18 ^a^	31.3
α		55.4	C=O		175.0	C=O		173.8
β	1.33 s	23.0	α		55.4	N-H_2_	7.17 br s	108.2
γ	1.37 s	25.4	β	1.31 s	22.0		6.73 br s	
**Aib^4^**			γ	1.37 s	26.1	**Leuol^15^**		
NH	8.06 s	124.4	**Aib^10^**			NH	7.07 d 8.4	119.1
C=O		176.4	NH	7.56 s	125.1	α	3.76 m	48.4
α		55.4	C=O		175.9	β	1.34 ^a^	39.5
β	1.32 s	22.0	α		55.5	γ	1.64 ^a^	23.7
γ	1.37 s	26.5	β	1.38 s	22.6	δ_1_	0.85 d 6.6	23.2
**Leu^5^**			γ	1.48 s	26.2	δ_2_	0.81 d 6.6	21.3
NH	7.65 d 5.4	114.8	**Gln^11^**			β’	3.18 m, 3.30 m	63.6
C=O		173.8	NH	7.74 d 5.4	111.9	O-H	4.54 t-like	
α	3.88 ^a^	54.2	C=O		173.5			

Chemical shifts of quaternary carbons were determined from ^1^H, ^13^C HMBC correlation peaks; ^a^ overlapping signals, chemical shifts were determined from ^1^H, ^15^N or ^1^H, ^13^C HSQC correlation peaks.

**Table 4:** Biological activity of ampullosorin F (**1**), G (**2**) and A (**3**) (IC_50_, μM).

Compound	Toxicity ^a^		Antifungal Activity ^b^		
	HT29	PC3	*B. cinerea*	*S. tritici*	*P. infestans*
**1**	5.90 ± 0.65	3.62 ± 0.49	7.27 ± 0.95	>125	14.75 ± 1.89
**2**	6.04 ± 0.64	3.35 ± 0.89	4.59 ± 0.34	>125	14.79 ± 2.84
**3**	10.45 ± 1.18	6.68 ± 1.67	11.41 ± 1.48	>125	19.49 ± 4.29
Epoxiconazole ^c^			<1.5	<1.5	
Terbinafine ^c^					48.30 ± 0.2

^a^ Data represent biological quadruplicates (*n* = 4), each comprising technical triplicates. ^b^ The experiment was carried out in triplicates (*n* = 3), each comprising technical triplicates. ^c^ Used as positive control.

## Data Availability

The data presented in this study are available on request from the corresponding author.

## Supplementary Materials

Supplementary materials are available online at https://www.mdpi.com/article/10.3390/ijms222312718/s1.
