# Supplementary material for: Rare Glutamic Acid Methyl Ester Peptaibols from Sepedonium ampullosporum Damon KSH 534 Exhibit Promising Antifungal and Anticancer Activity
Source: Int J Mol Sci. 2021 Nov 24;22(23):12718. doi: 10.3390/ijms222312718 (PMC8657771; doi:10.3390/ijms222312718)

Rare glutamic acid methyl ester peptaibols from *Sepedonium ampullosporum* Damon KSH 534 exhibit promising anticancer activity

Yen T. H. Lam [1,2], Manuel G. Ricardo [1,3], Robert Rennert [1], Andrej Frolov [1,4], Andrea Porzel [1], Wolfgang Brandt [1], Pauline Stark [1], Bernhard Westermann [1] and Norbert Arnold [1,*]

[1] Department of Bioorganic Chemistry, Leibniz Institute of Plant Biochemistry, Weinberg 3, D-06120 Halle (Saale), Germany; ThiHaiYen.Lam@ipb-halle.de (Y.T.H.L.); Robert.Rennert@ipb-halle.de (R.R.); Andrej.Frolov@ipb-halle.de (A.F.); Andrea.Porzel@ipb-halle.de (A.P.); Wolfgang.Brandt@ipb-halle.de (W.B.); Pauline.Stark@ipb-halle.de (P.S.); Bernhard.Westermann@ipb-halle.de (B.W.); Norbert.Arnold@ipb-halle.de (N.A.)

[2] Department of Organic Chemistry, Faculty of Chemistry, Hanoi National University of Education, 136 Xuanthuy, Caugiay, Hanoi, Vietnam;

[3] Department of Biomolecular Systems, Max Planck Institute of Colloids and Interfaces, Am Mühlenberg 1, D-14476 Potsdam, Germany; Manuel.GarciaRicardo@mpikg.mpg.de (M.G.R.)

[4] Department of Biochemistry, Faculty of Biology, St. Petersburg State University, Sredny Prospekt V.O. 41, 199004, St. Petersburg, Russian Federation;

* Correspondence: Norbert.Arnold@ipb-halle.de; Tel.: +49-345-5582-1310

Supplementary Materials

Table of contents

- Spectral data of natural ampullosporins F (**1**) and G (**2**) ……………………………………...…………………… Figure S1-S16
- Spectral data of synthetic ampullosporins F (**1**) and G (**2**) ……………………………………………………..… Figure S17-S22
- Spectroscopic comparison of natural and synthetic ampullosporins F (**1**) and G (**2**) …………………...…..… Figure S23-S28
- Spectral data of natural peptides **3**-**7**…………………………………………………………………………...….. Figure S29-S40
- Antiphytophathogenic activity of ampullosporin F (**1**), G (**2**), and A (**3**) …………………………...….….……… Figure S41
- Docking arrangement of ampullosporin G (**2**) on protein databank entry 6IRA ………..……………….………… Figure S42
- LC-HRMS spectrum of the enriched fraction from ethanol extract of *S. ampullosporum* strain KSH 534 …..……..... Figure S43
- ESI-HRMS^n data of **1** – **5** …………………………………………………………………………………….….…... Table S1-S2

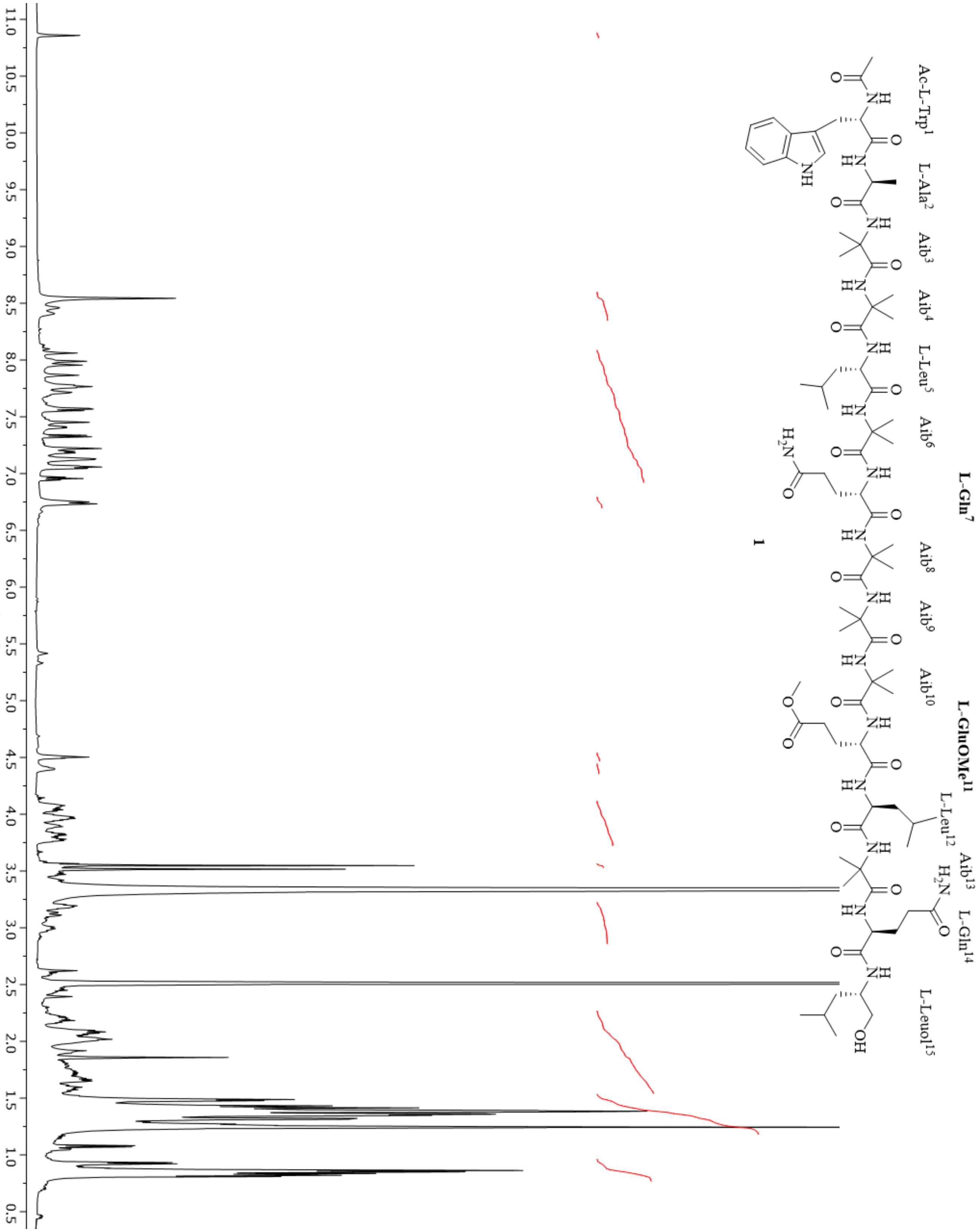

Figure S1. ^{1}H NMR spectrum of natural ampullosporin F (**1**) (600 MHz, DMSO-d_6).

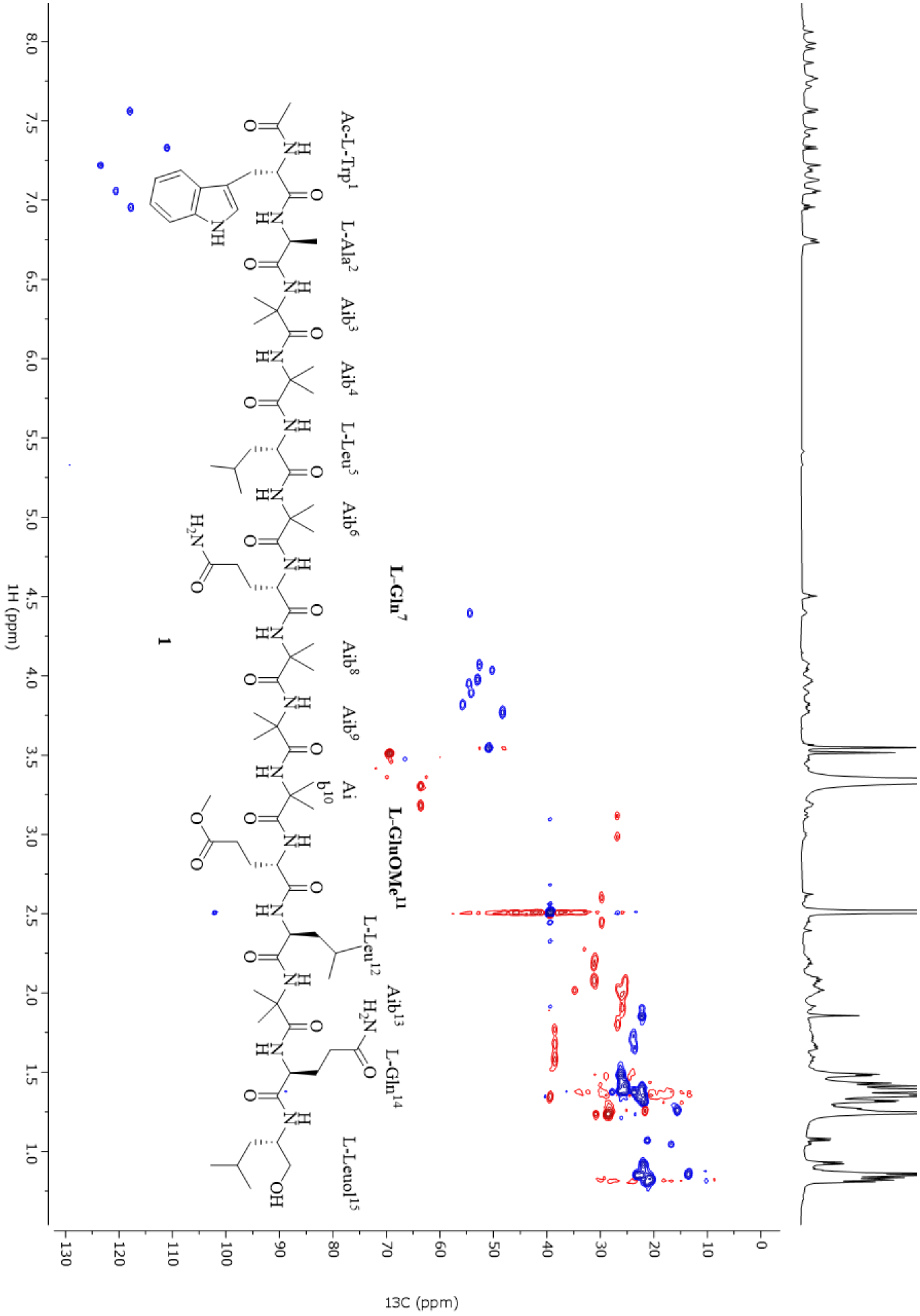

Figure S2. ^{1}H, ^{13}C HSQC spectrum of natural ampullosporin F (**1**) (600 MHz, DMSO-d_6).

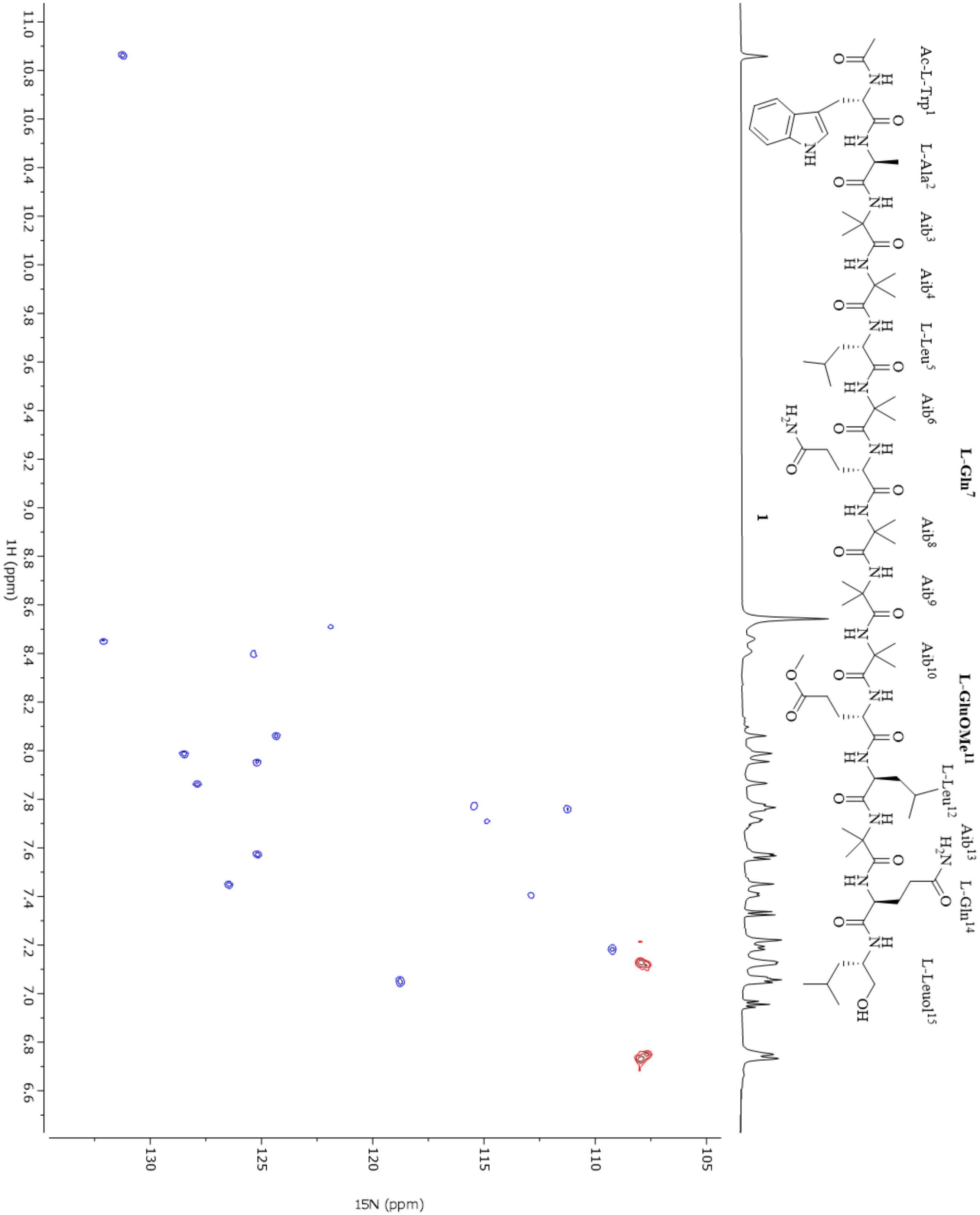

Figure S3. ^{1}H, ^{15}N HSQC spectrum of natural ampullosporin F (**1**) (600 MHz, DMSO-d_6).

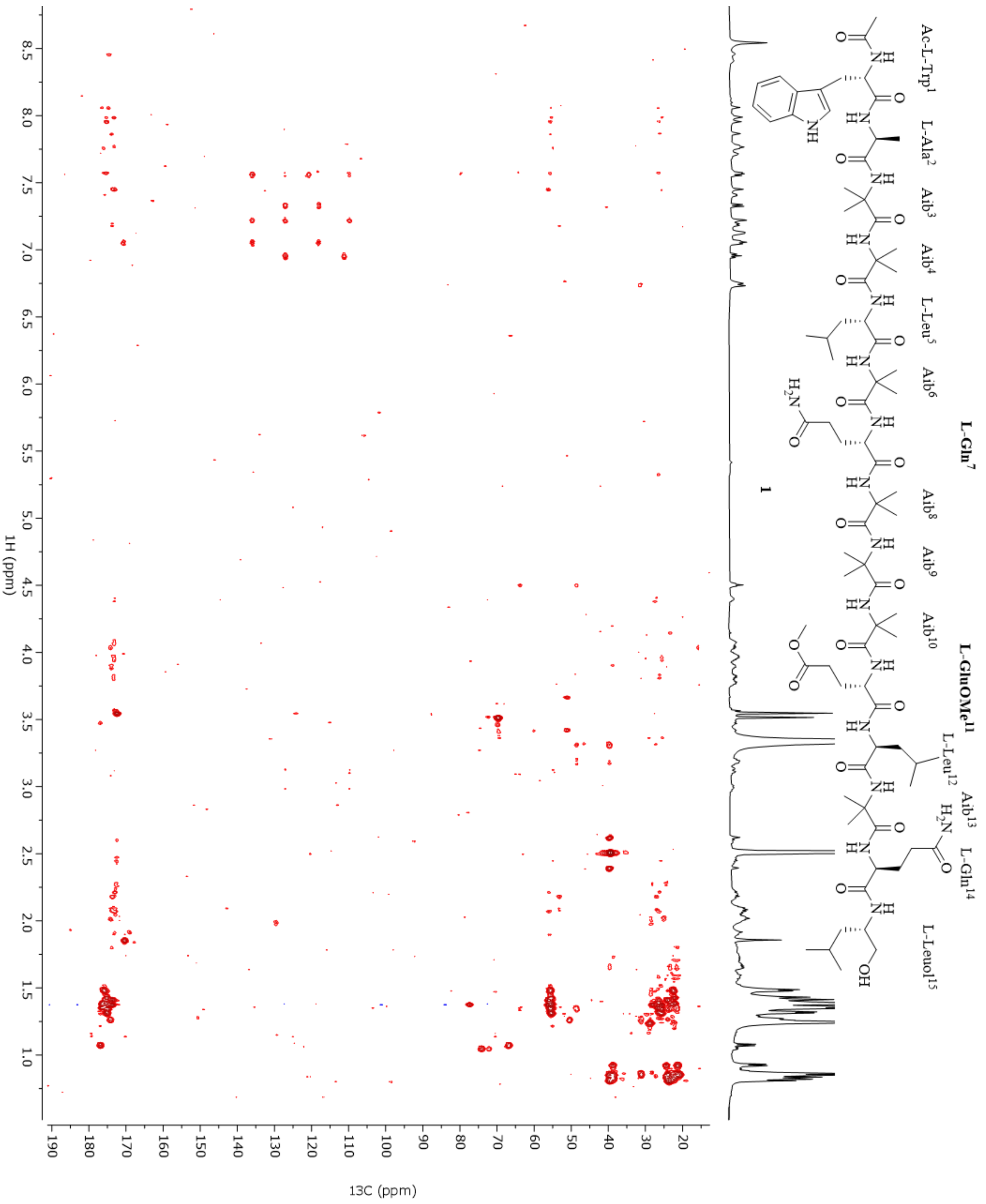

Figure S4. ^{1}H, ^{13}C HMBC spectrum of natural ampullosporin F (**1**) (600 MHz, DMSO-d_6).

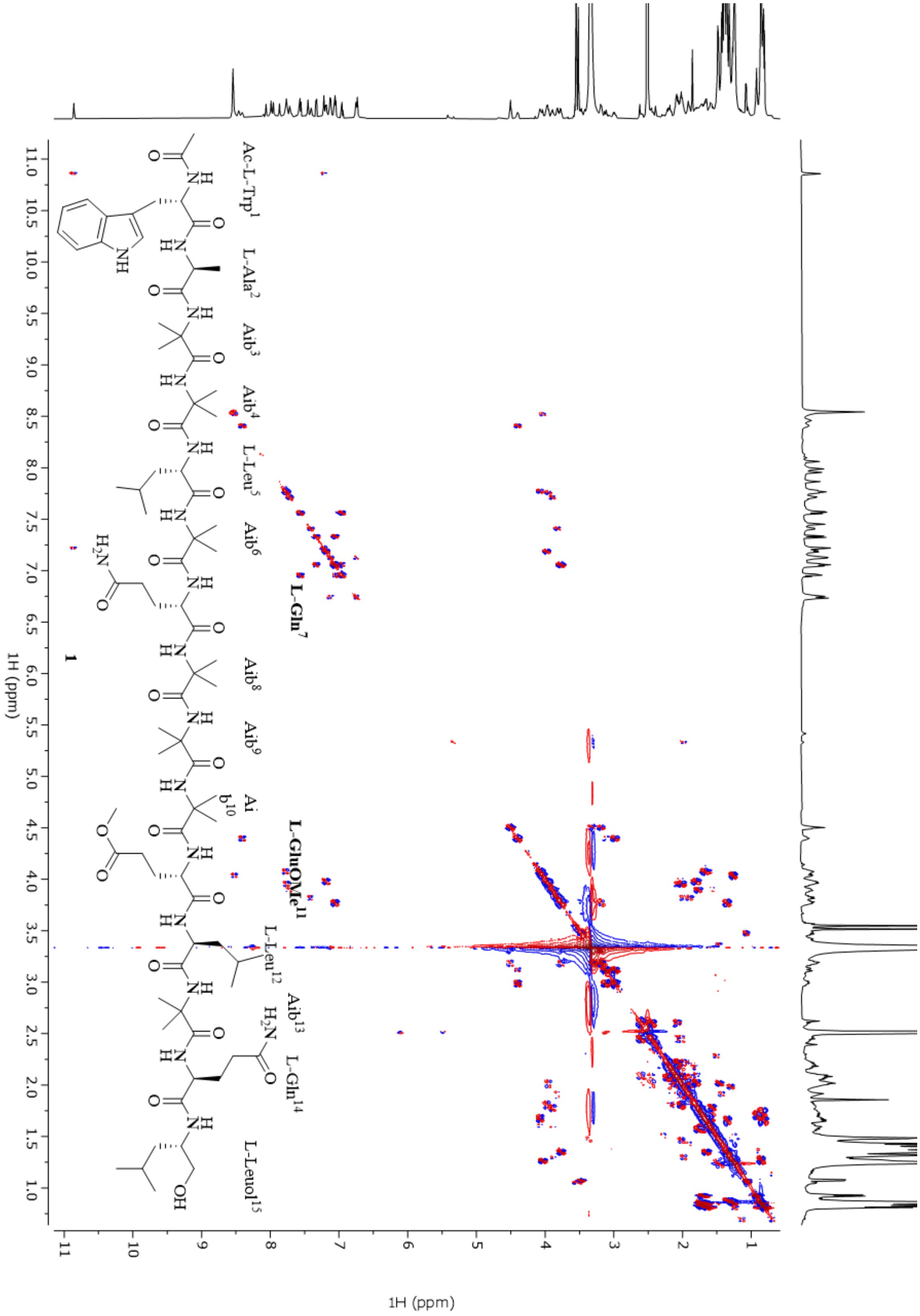

Figure S5. ^{1}H, ^{1}H COSY spectrum of natural ampullosporin F (**1**) (600 MHz, DMSO-d_6).

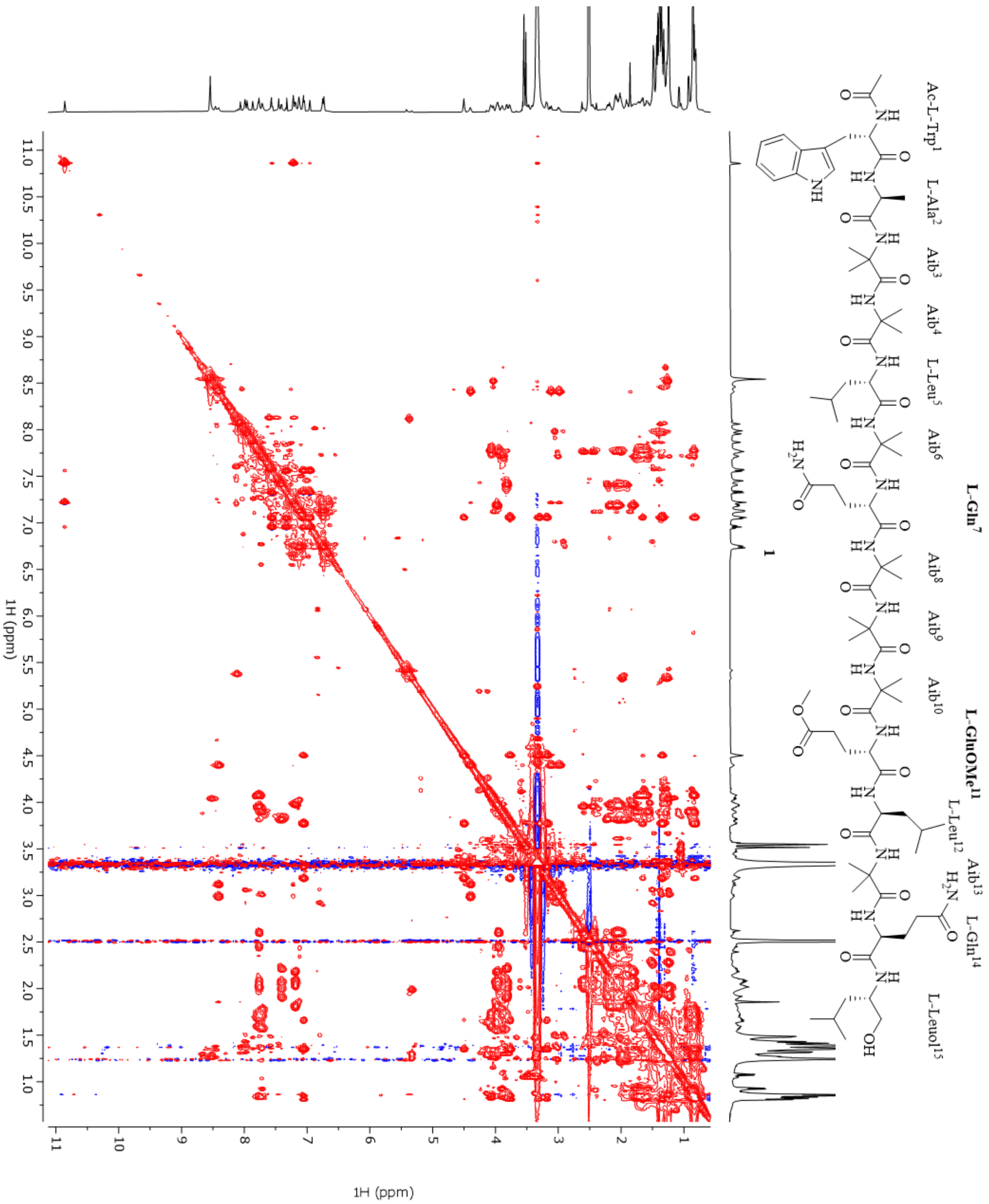

Figure S6. ^{1}H, ^{1}H TOCSY spectrum of natural ampullosporin F (**1**) (600 MHz, DMSO-d_6).

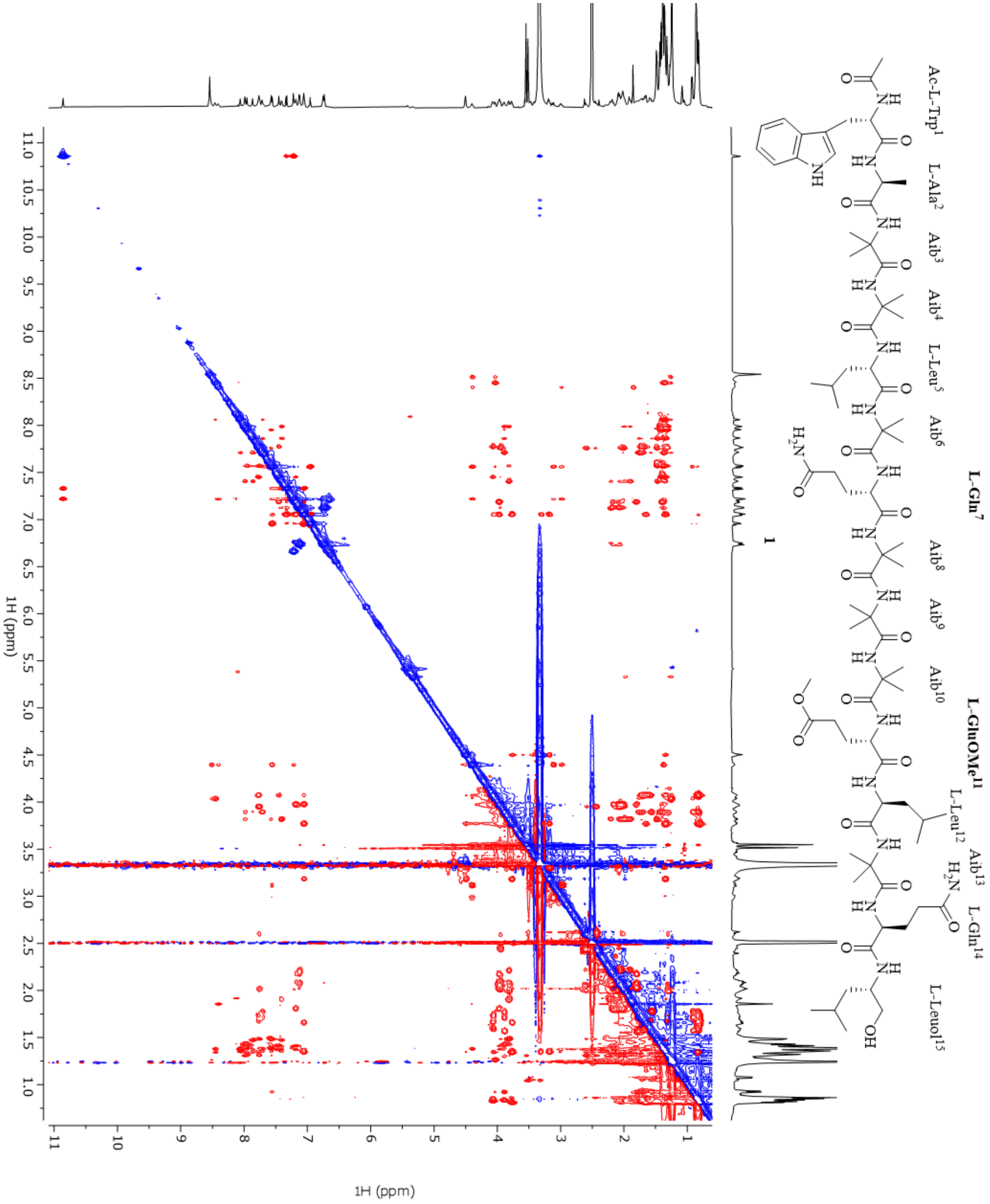

Figure S7. ^{1}H, ^{1}H ROESY spectrum of natural ampullosporin F (**1**) (600 MHz, DMSO-d_6).

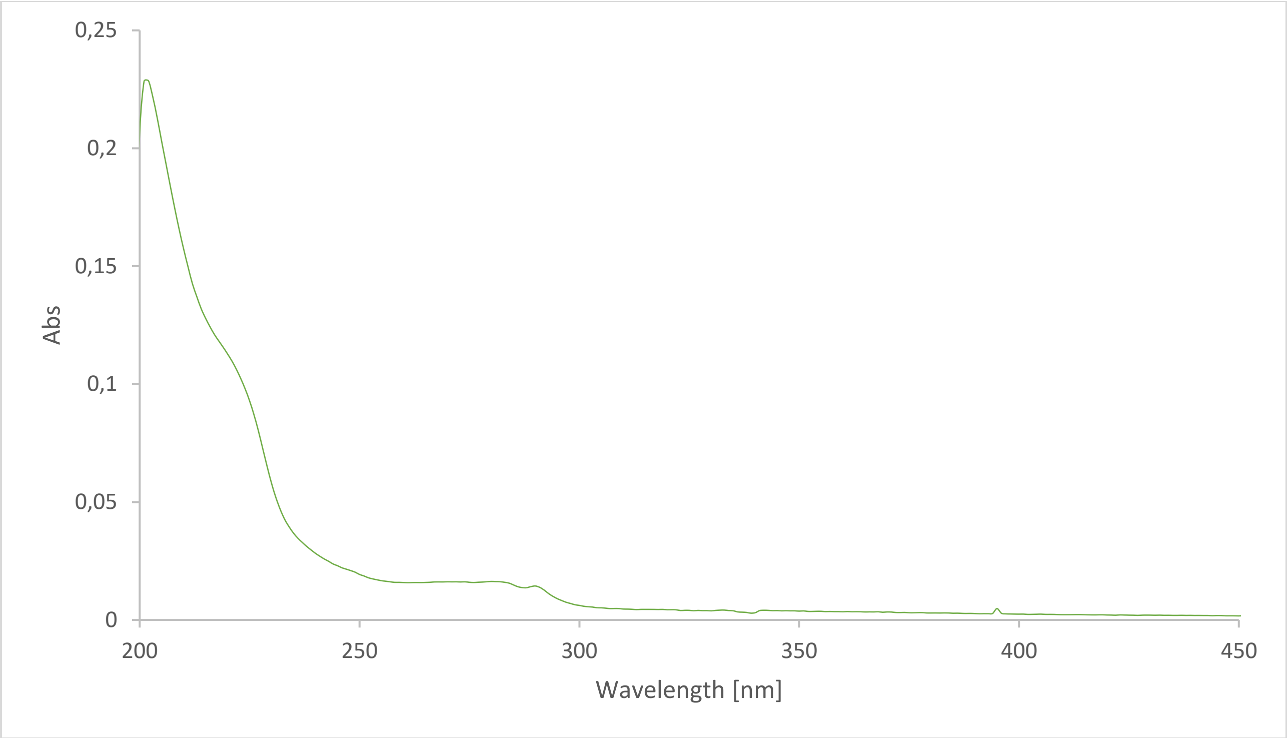

Figure S8. UV spectrum of natural ampullosporin F (**1**) in MeOH.

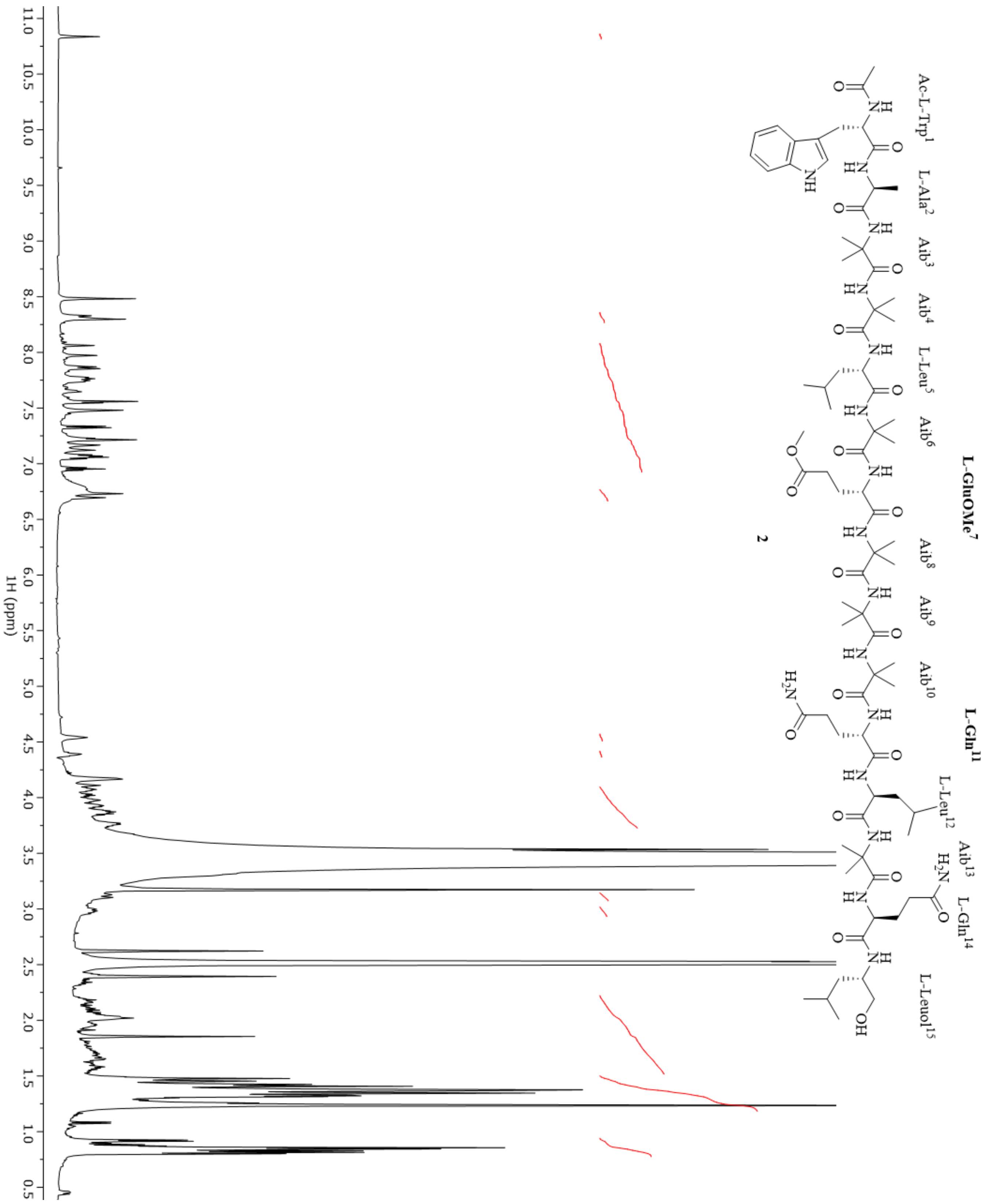

Figure S9. ^{1}H NMR spectrum of natural ampullosporin G (**2**) (600 MHz, DMSO-d_6).

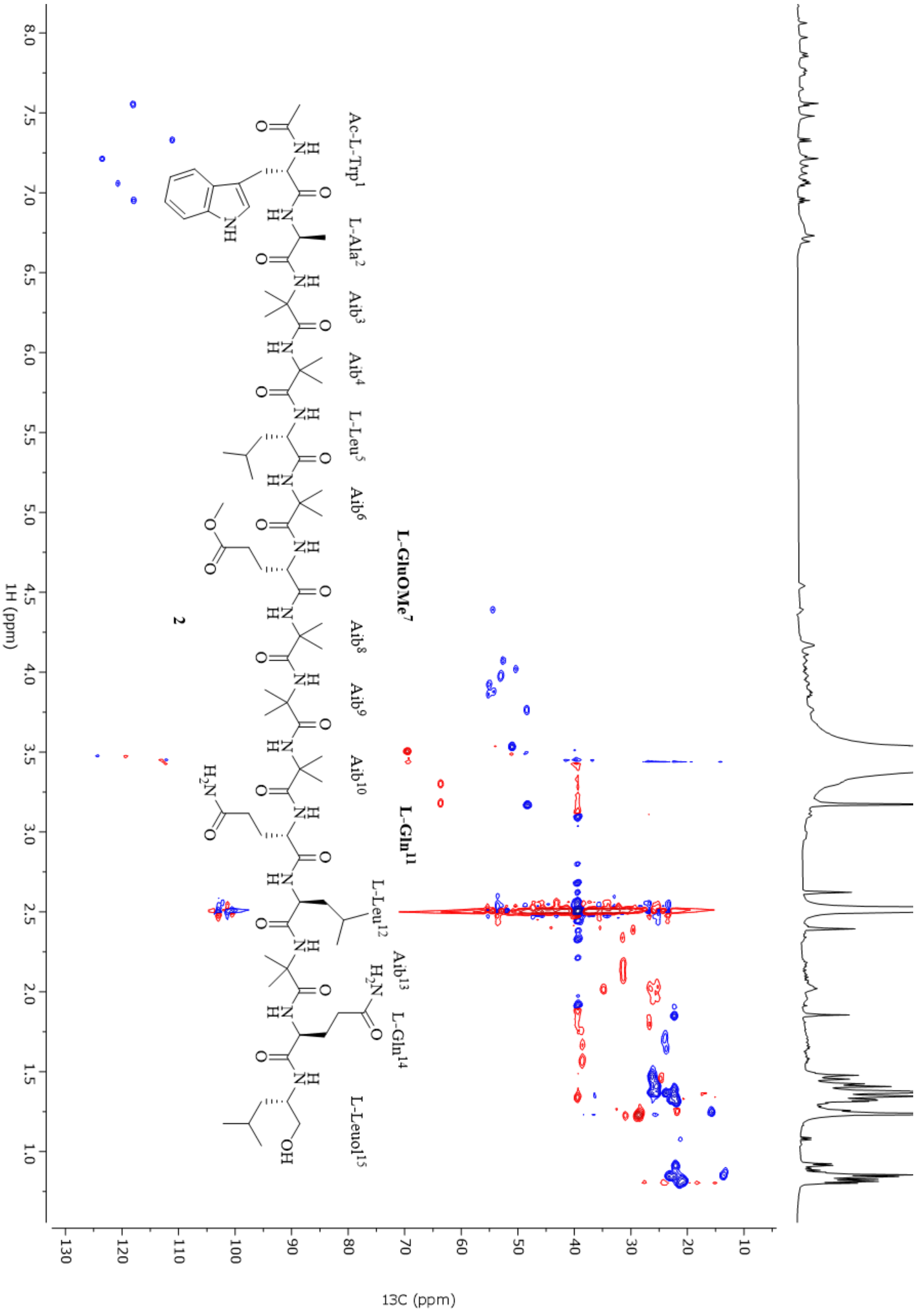

Figure S10. ^{1}H, ^{13}C HSQC spectrum of natural ampullosporin G (**2**) (600 MHz, DMSO-d_6).

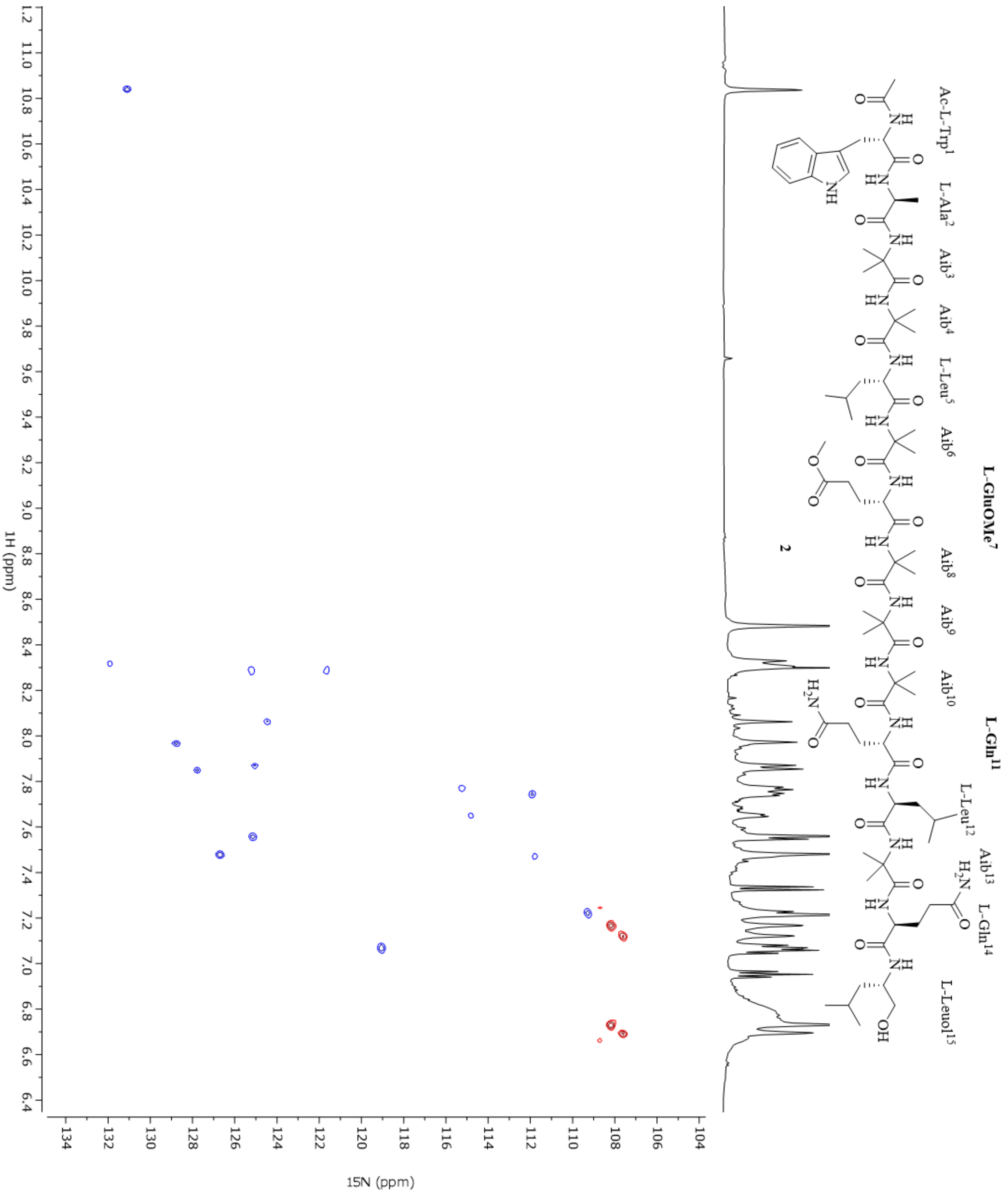

Figure S11. ^{1}H, ^{15}N HSQC spectrum of natural ampullosporin G (**2**) (600 MHz, DMSO-d_6).

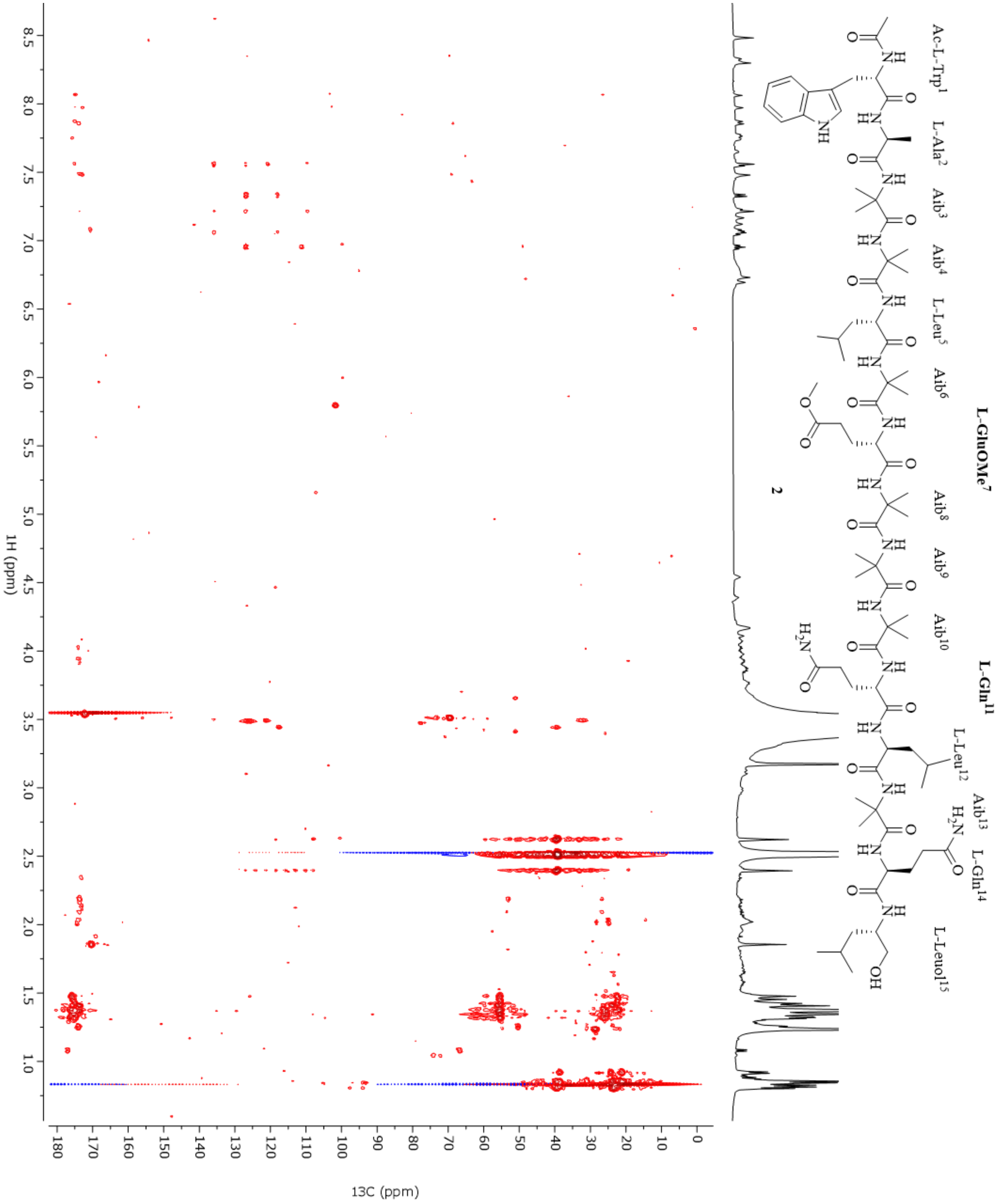

Figure S12. ^{1}H, ^{13}C HMBC spectrum of natural ampullosporin G (**2**) (600 MHz, DMSO-d_6).

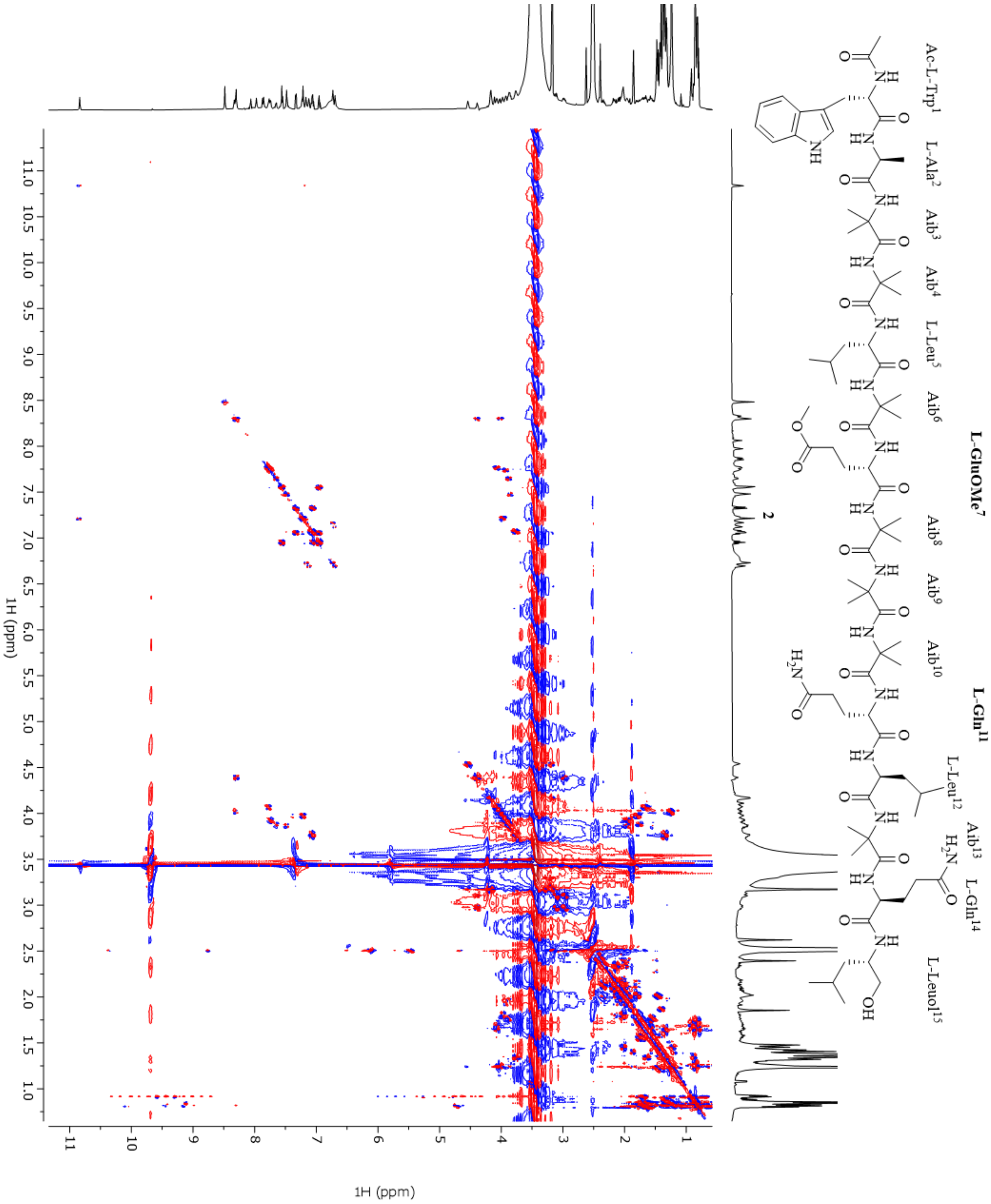

Figure S13. ^{1}H, ^{1}H COSY spectrum of natural ampullosporin G (**2**) (600 MHz, DMSO-d_6).

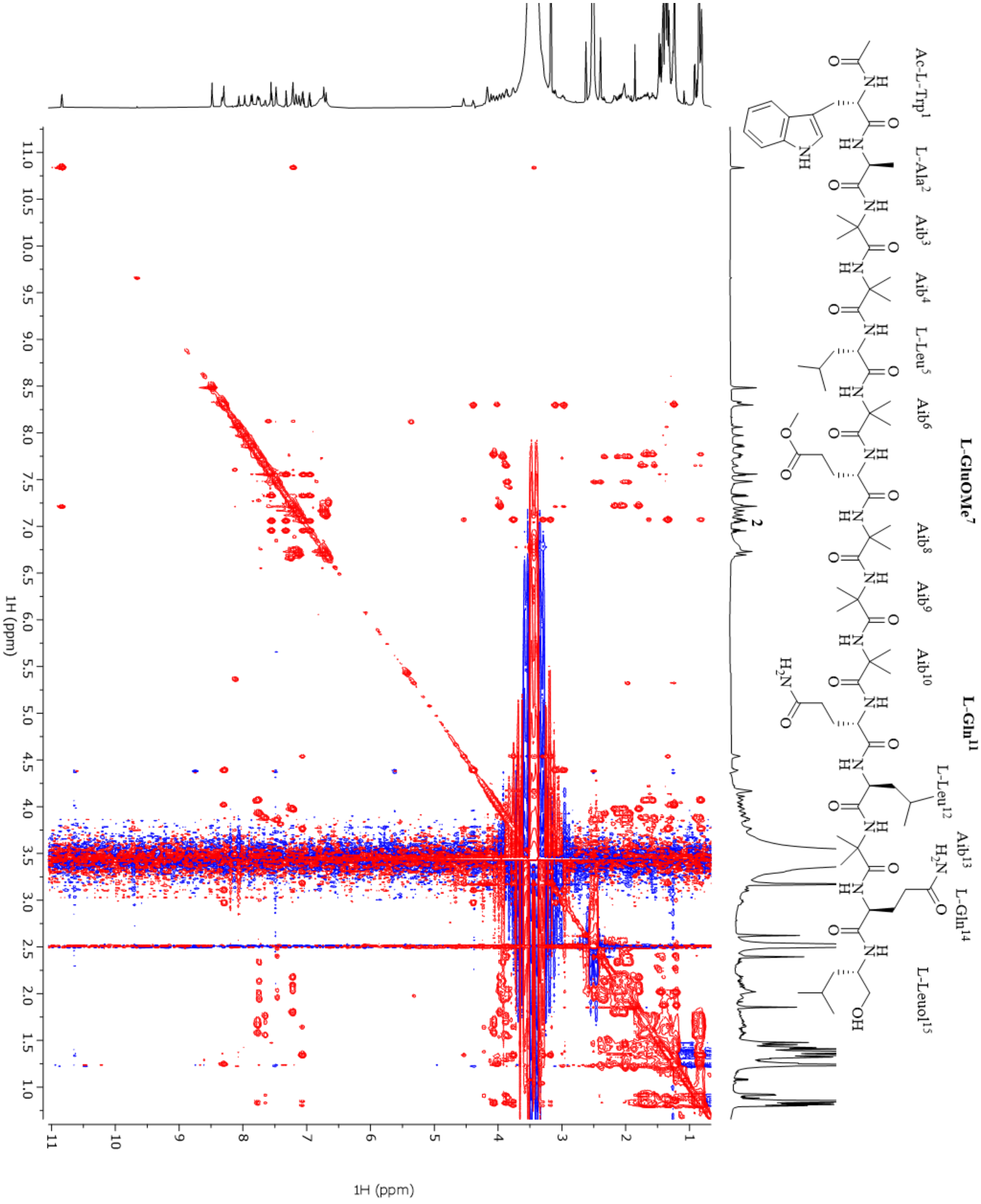

Figure S14. ^{1}H, ^{1}H TOCSY spectrum of natural ampullosporin G (**2**) (600 MHz, DMSO-d_6).

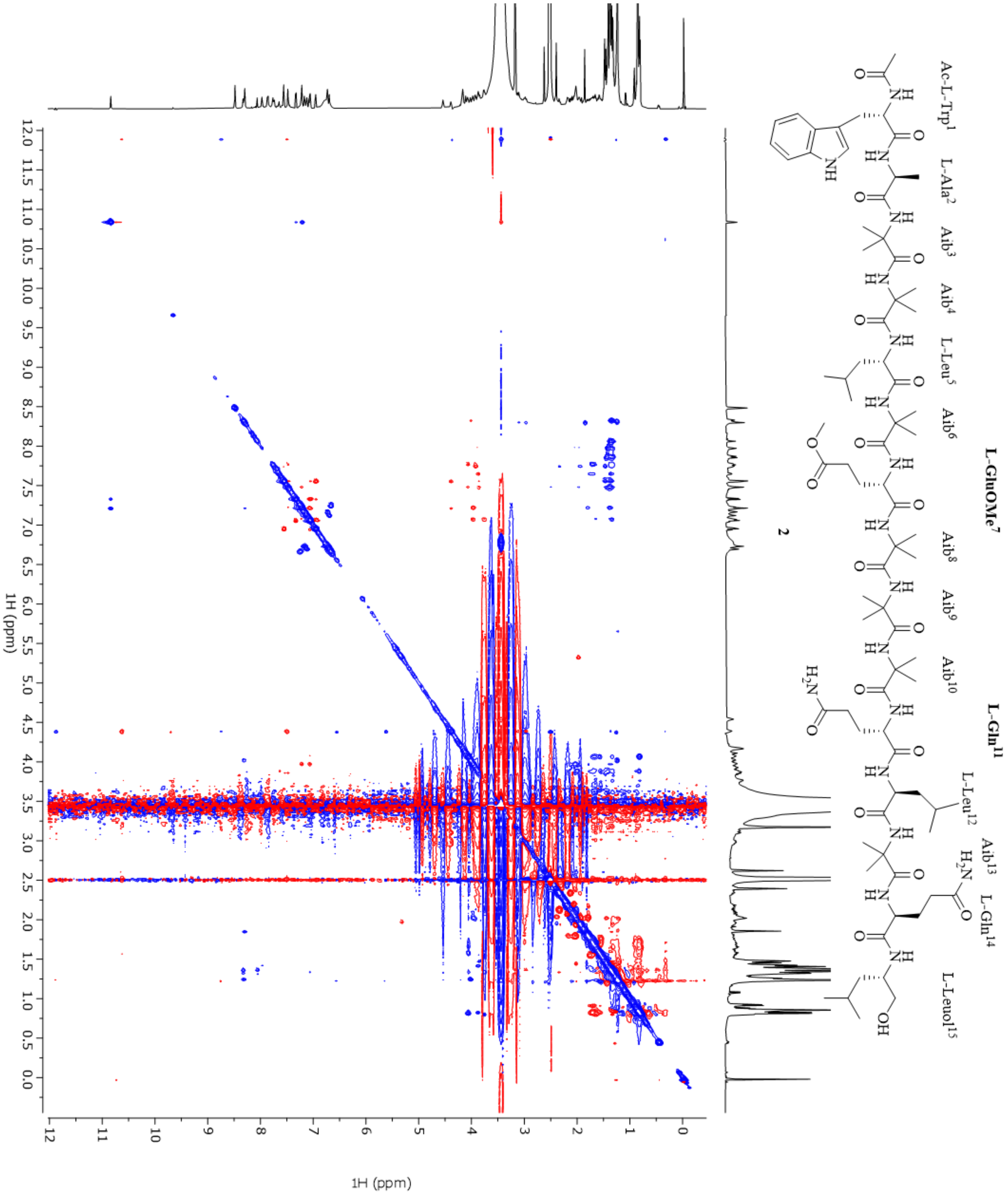

Figure S15. ^{1}H, ^{1}H ROESY spectrum of natural ampullosporin G (**2**) (600 MHz, DMSO-d_6).

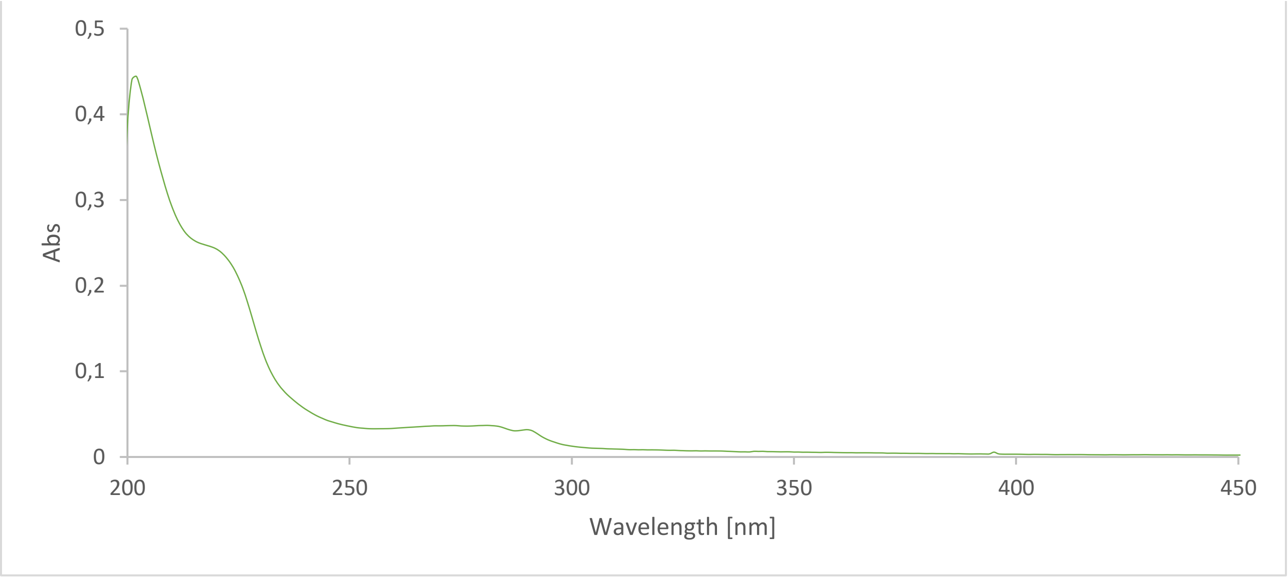

Figure S16. UV spectrum of natural ampullosporin G (**2**) in MeOH.

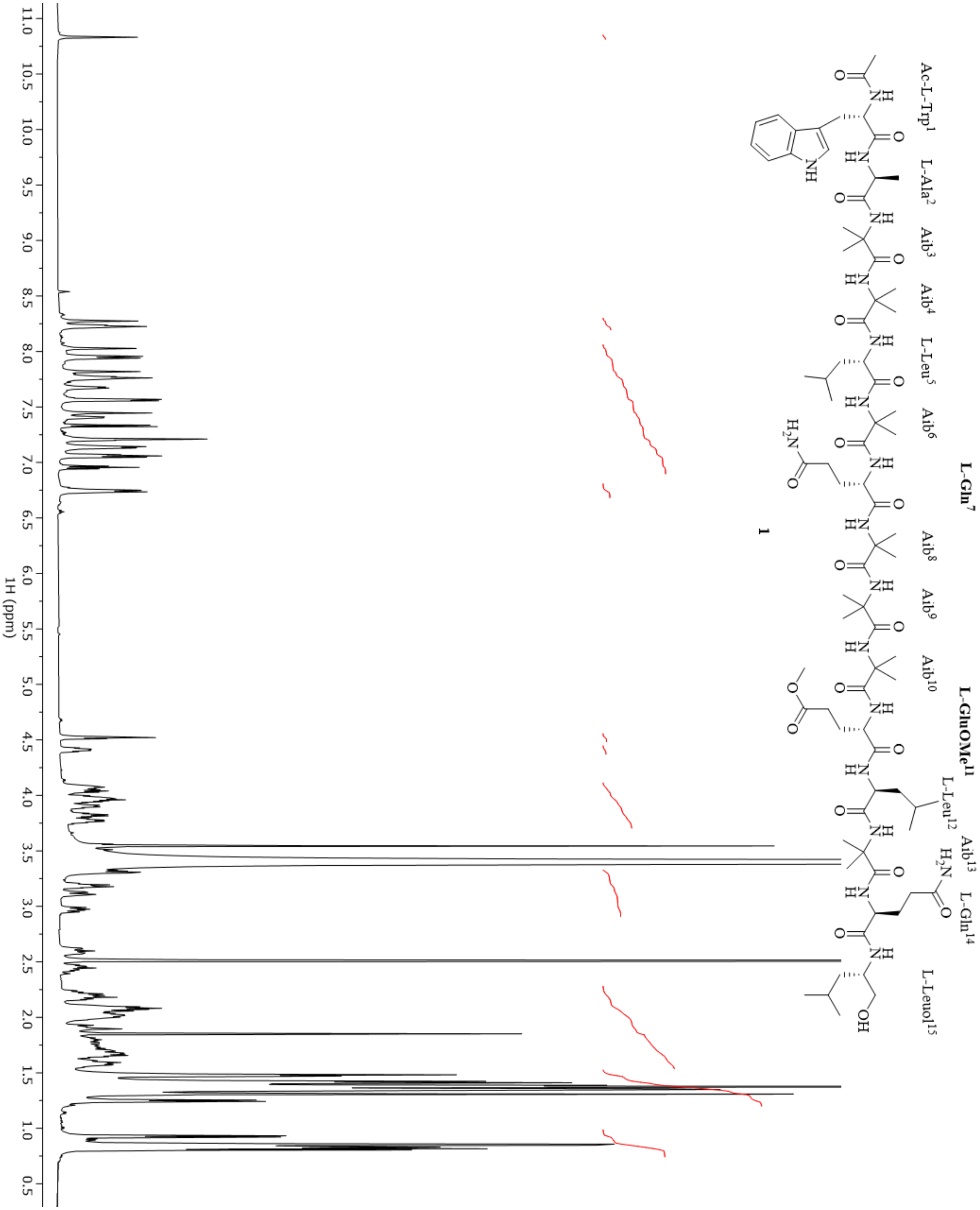

Figure S17. ^{1}H NMR spectrum of synthetic ampullosporin F (**1**) (600 MHz, DMSO-d_6).

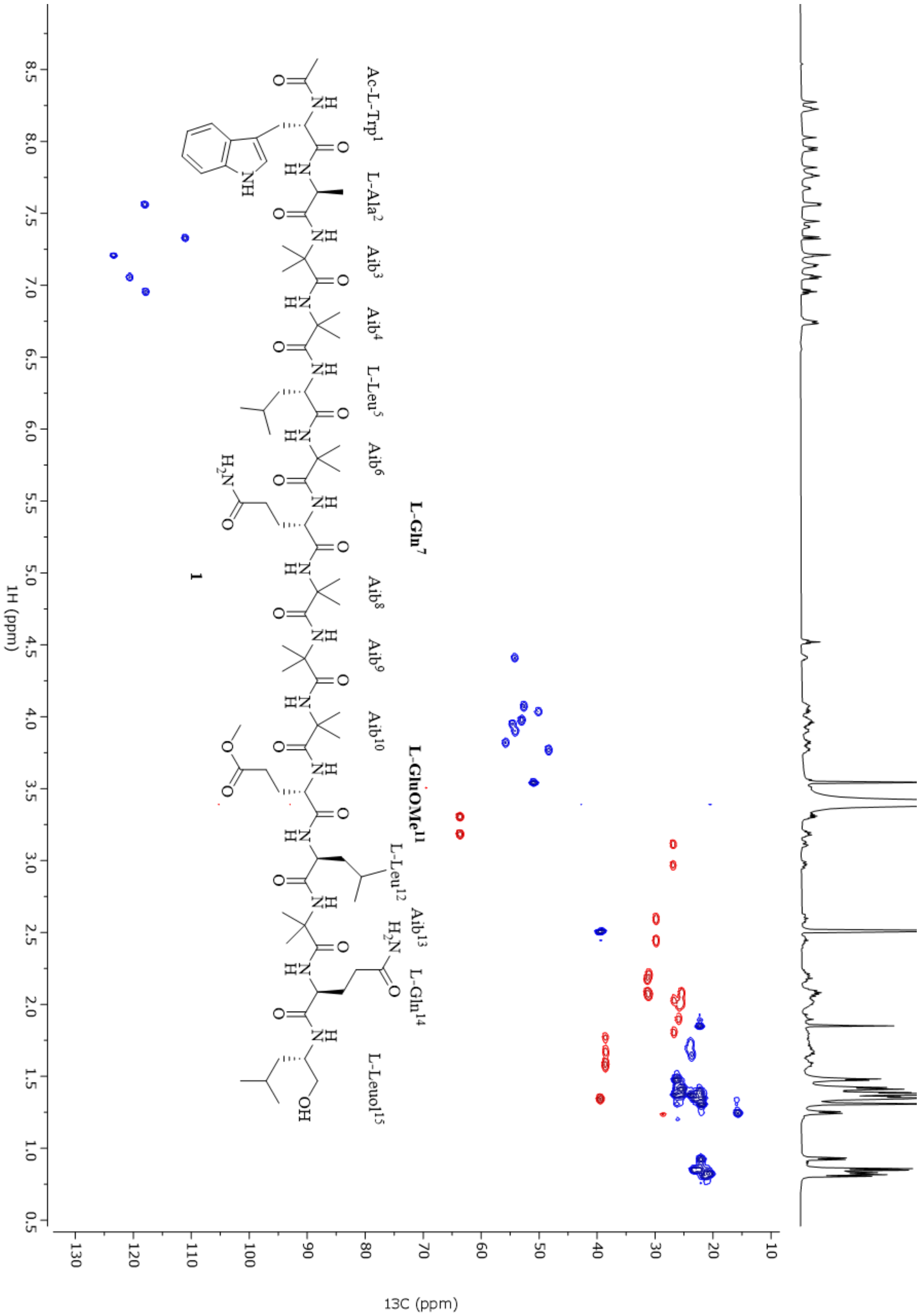

Figure S18. ^{1}H, ^{13}C HSQC spectrum of synthetic ampullosporin F (**1**) (600 MHz, DMSO-d_6).

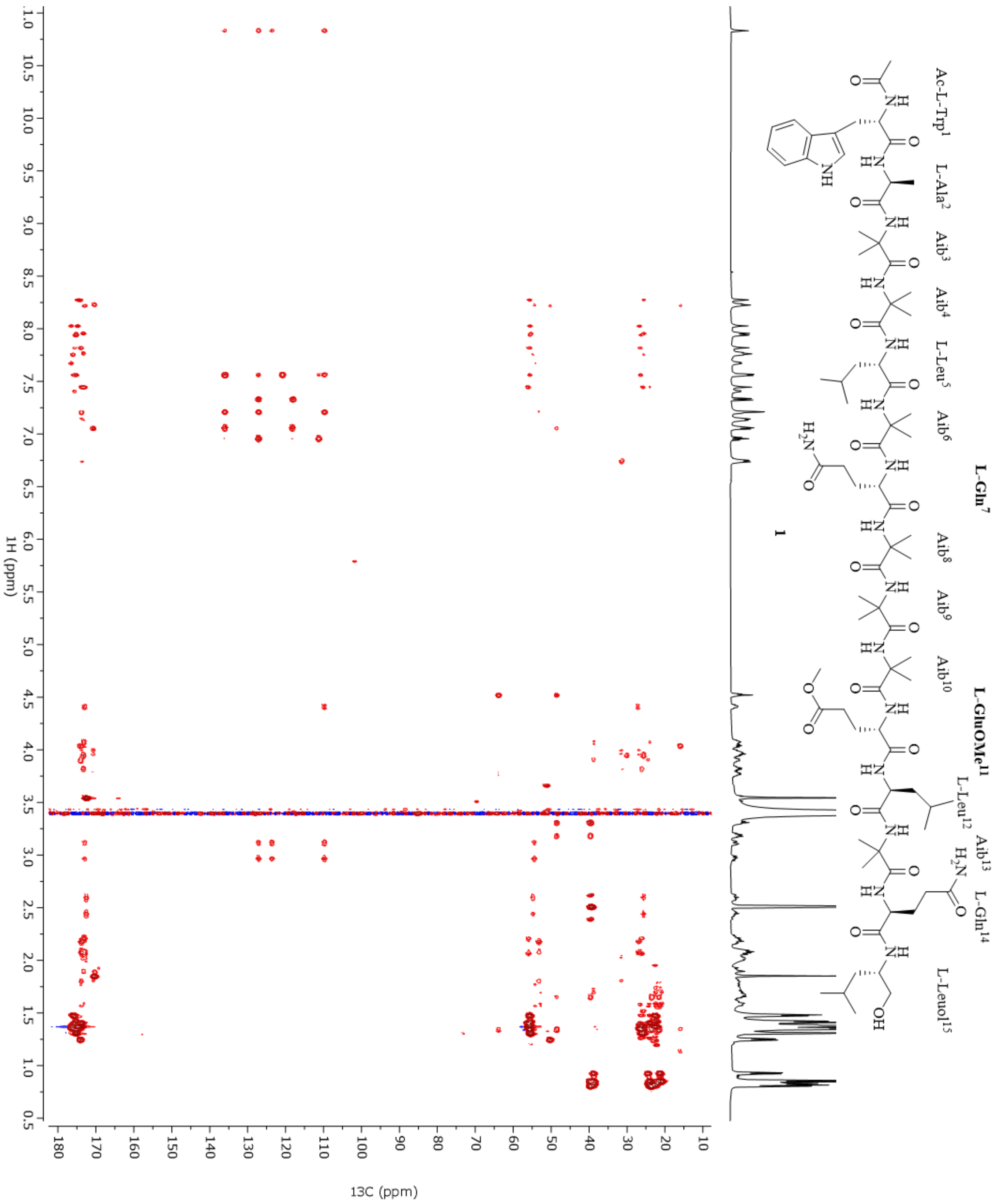

Figure S19. ^{1}H, ^{13}C HMBC spectrum of synthetic ampullosporin F (**1**) (600 MHz, DMSO-d_6).

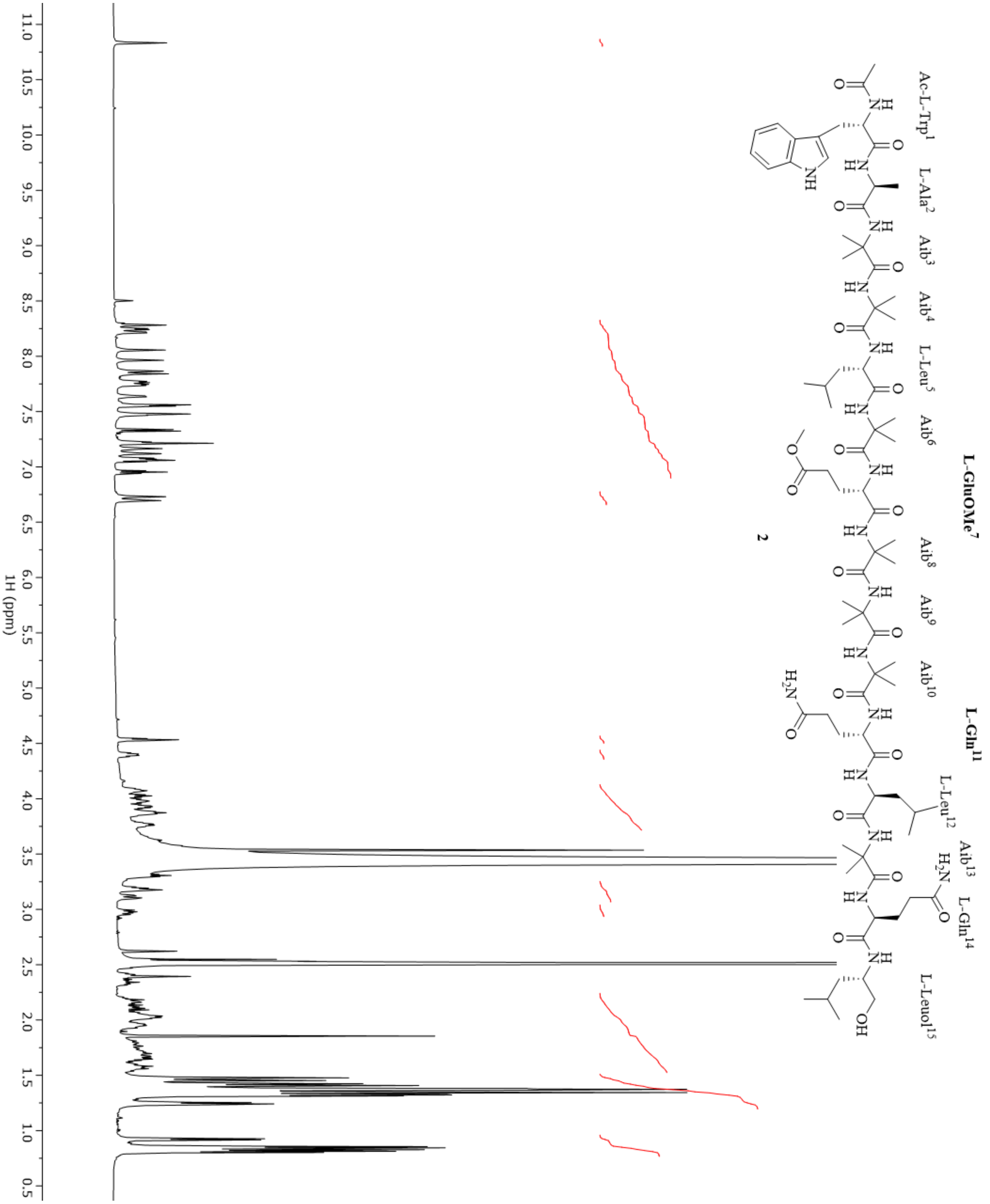

Figure S20. ^{1}H NMR spectrum of synthetic ampullosporin G (**2**) (600 MHz, DMSO-d_6).

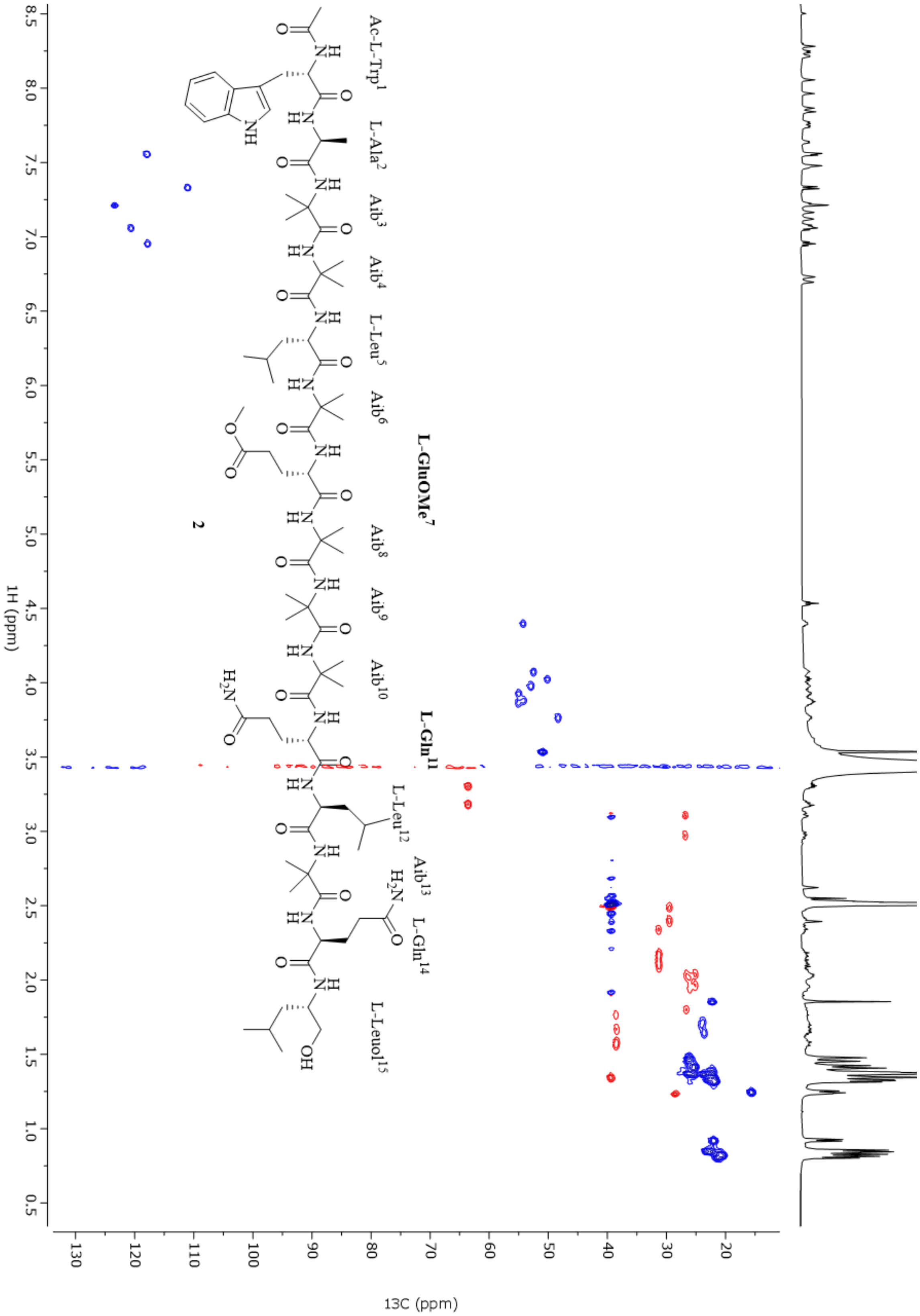

Figure S21. ^{1}H, ^{13}C HSQC spectrum of synthetic ampullosporin G (**2**) (600 MHz, DMSO-d_6).

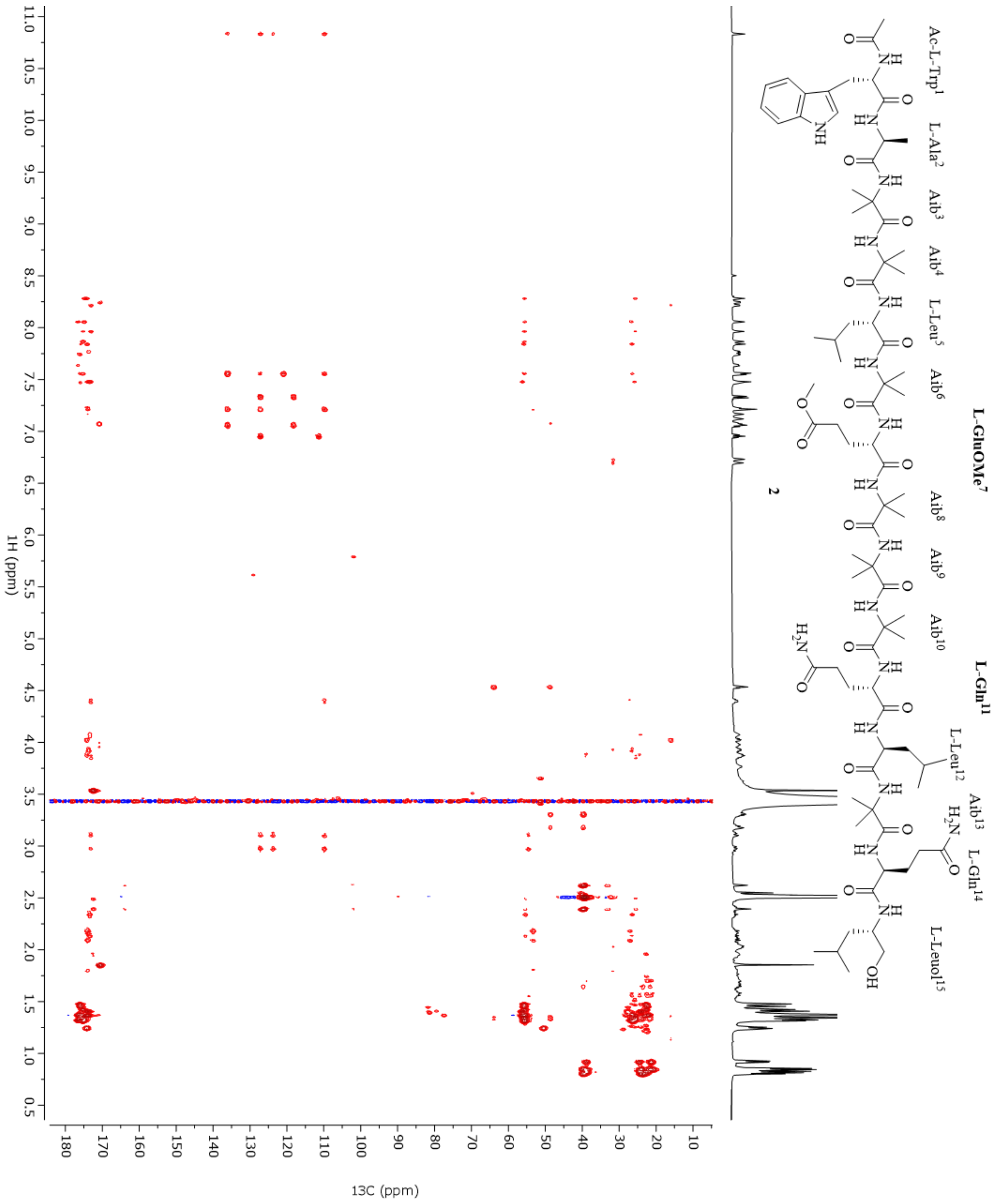

Figure S22. ^{1}H, ^{13}C HMBC spectrum of synthetic ampullosporin G (**2**) (600 MHz, DMSO-d_6).

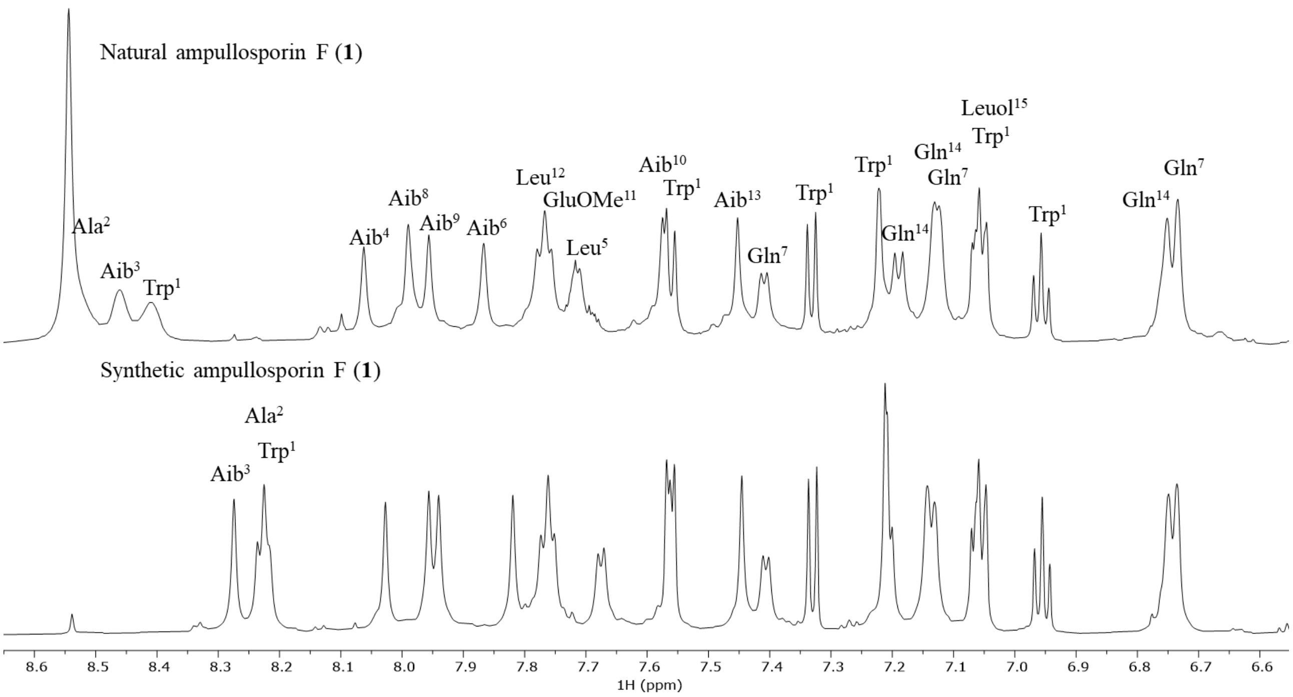

Figure S23. Comparison of the amide and aromatic region in the ^{1}H NMR of natural and synthetic ampullosporin F (**1**) (600 MHz, DMSO-d_6).

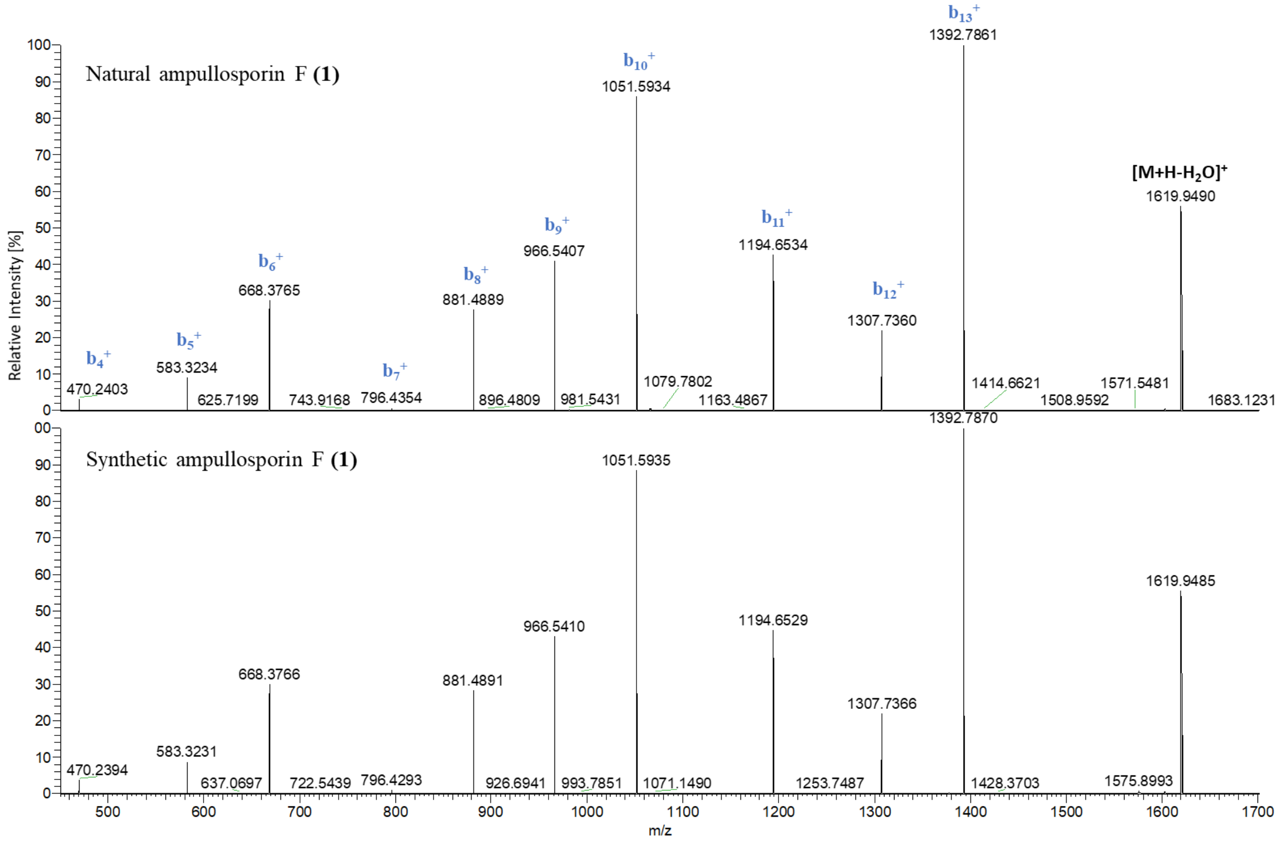

Figure S24. Positive ion MS^2 spectra of the $[M+H]^+$ of natural and synthetic ampullosporin F (**1**).

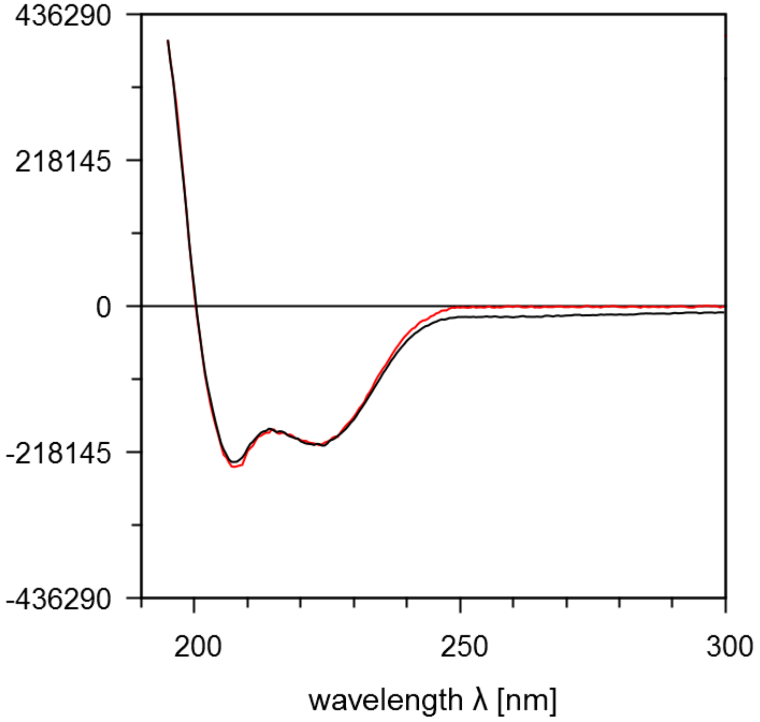

Figure S25. CD spectra of natural (in red) and synthetic (in black) ampullosporin F (**1**) in MeOH.

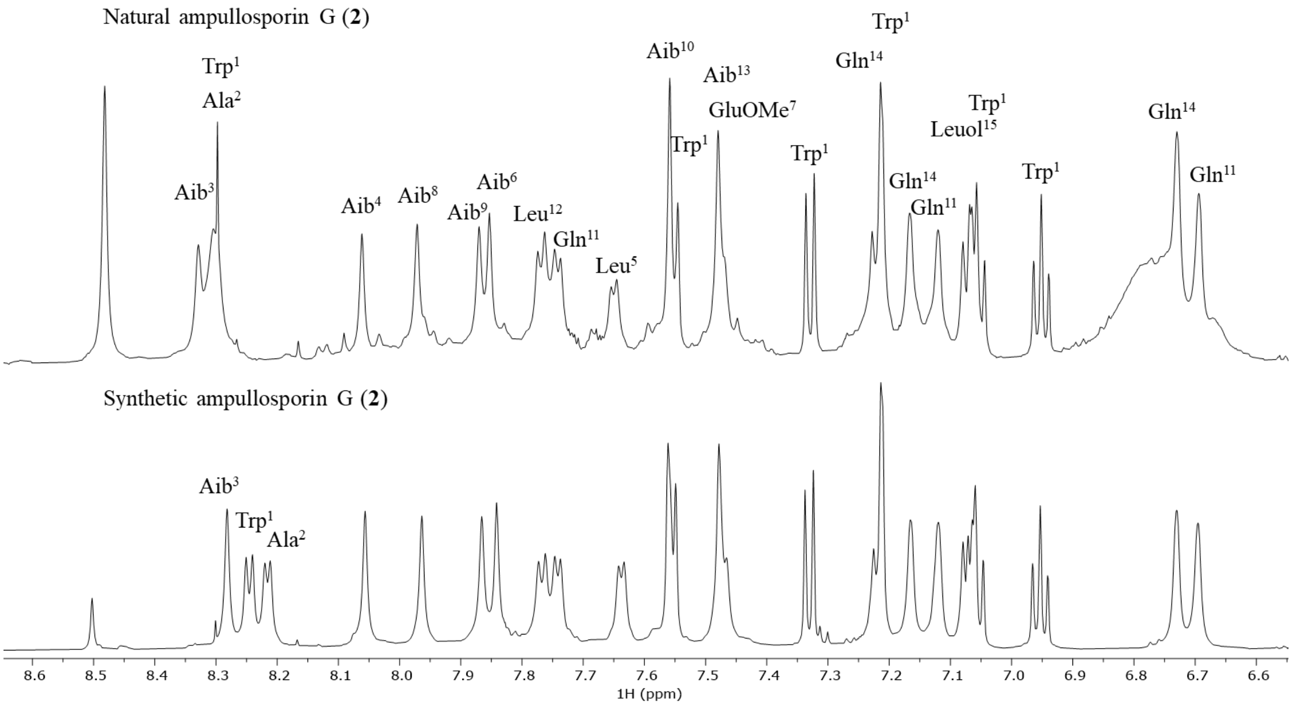

Figure S26. Comparison of the amide and aromatic region in the ^{1}H NMR of natural and synthetic ampullosporin G (**2**) (600 MHz, DMSO-d_6).

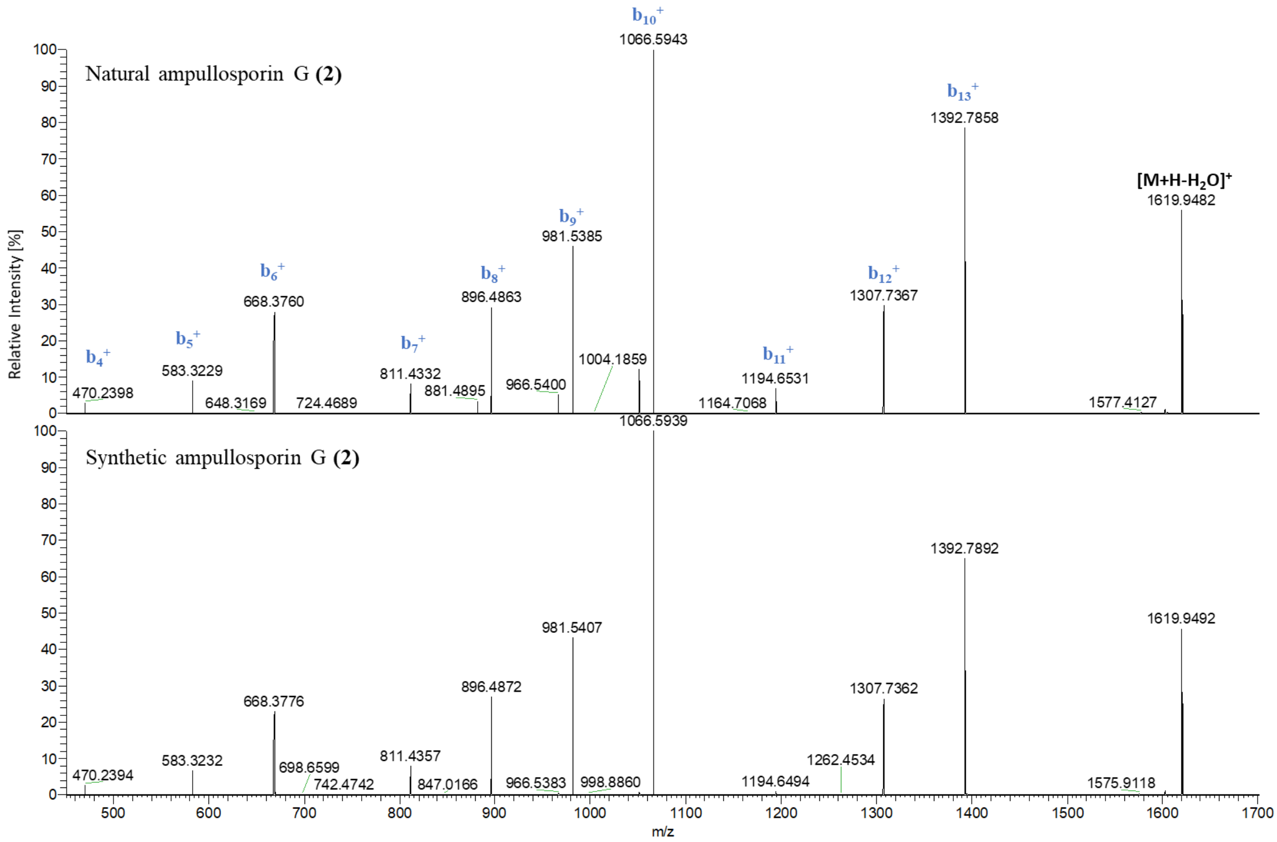

Figure S27. Positive ion MS^2 spectra of the $[M+H]^+$ of natural and synthetic ampullosporin G (**2**).

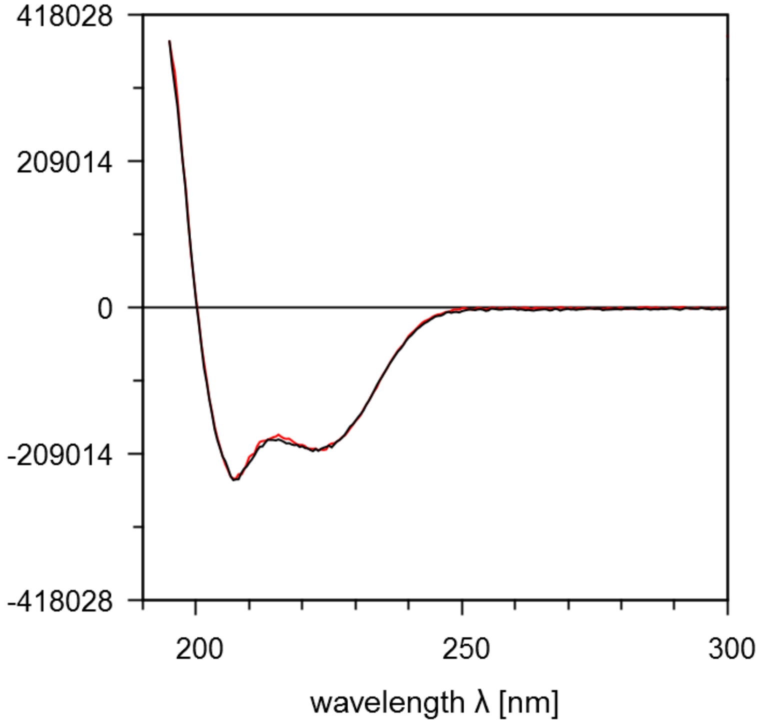

Figure S28. CD spectra of natural (in red) and synthetic (in black) ampullosporin G (**2**) in MeOH.

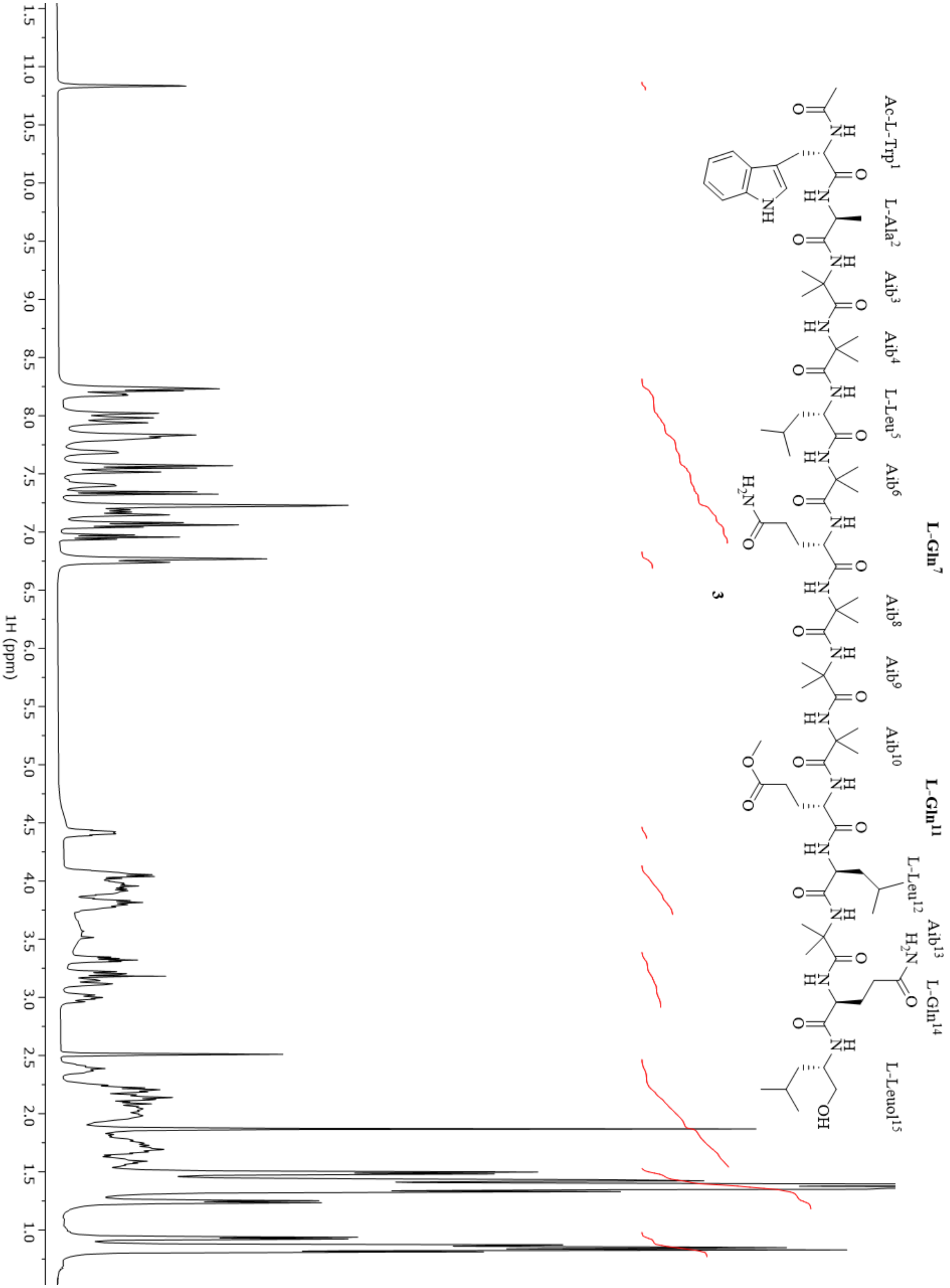

Figure S29. ^{1}H NMR spectrum of natural ampullosporin A (**3**) (400 MHz, DMSO-d_6).

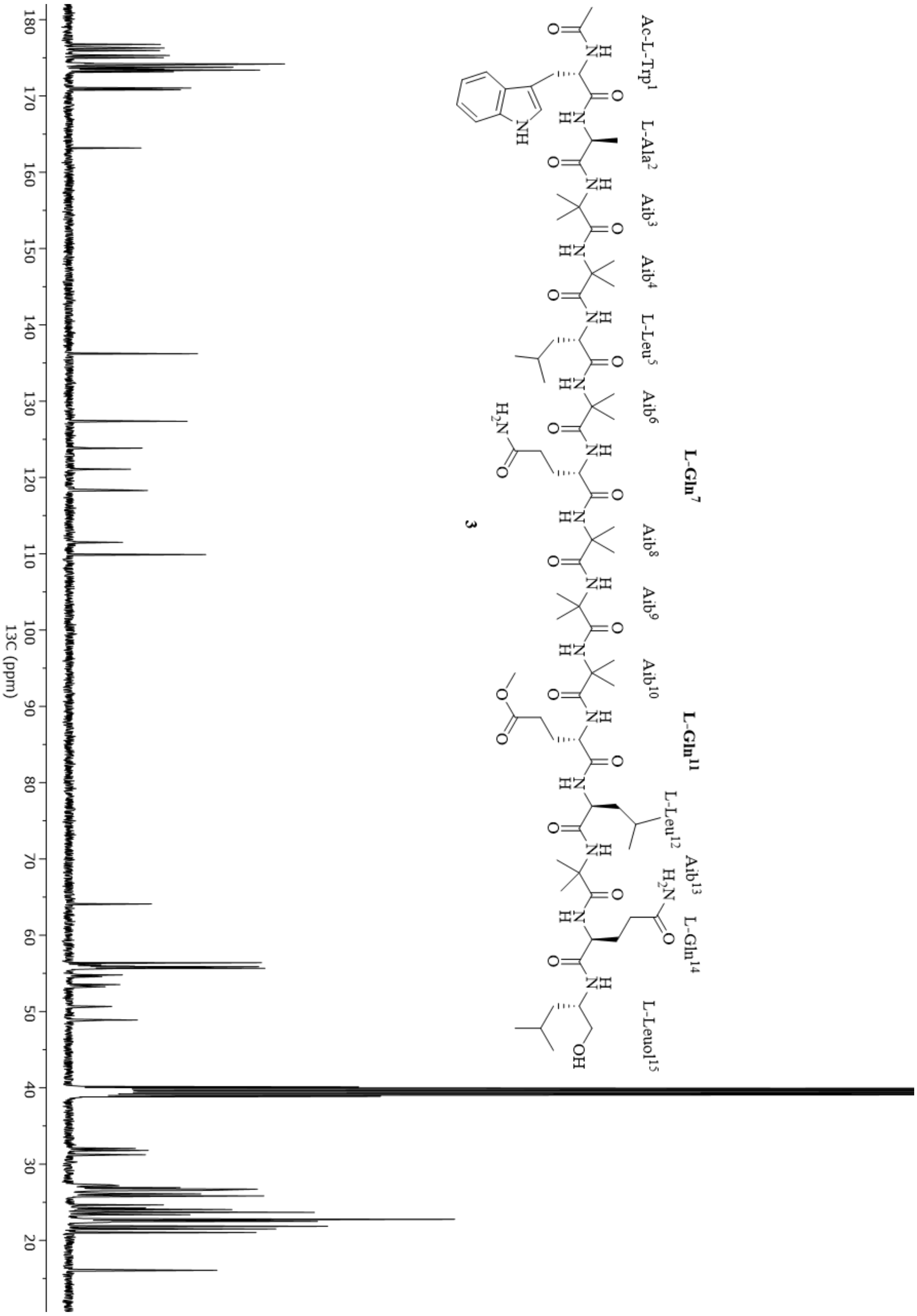

Figure S30. ^{13}C NMR spectrum of natural ampullosporin A (**3**) (100 MHz, DMSO-d_6).

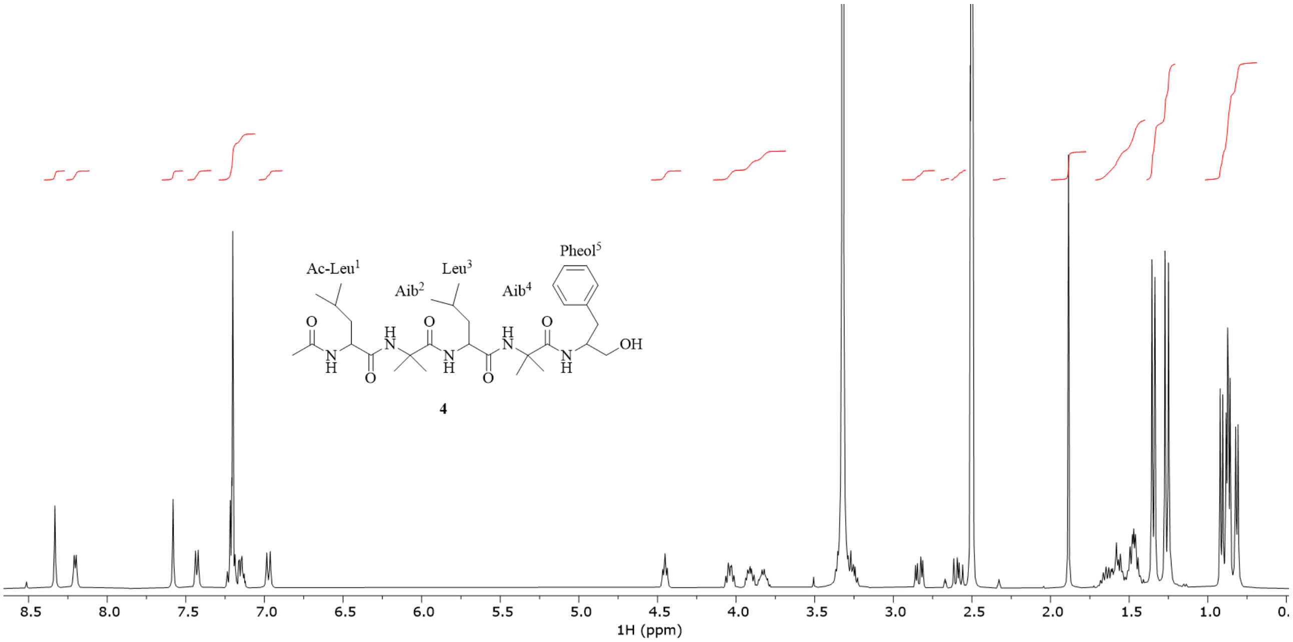

Figure S31. ^{1}H NMR spectrum of natural peptaibolin (**4**) (400 MHz, DMSO-*d*$_6$).

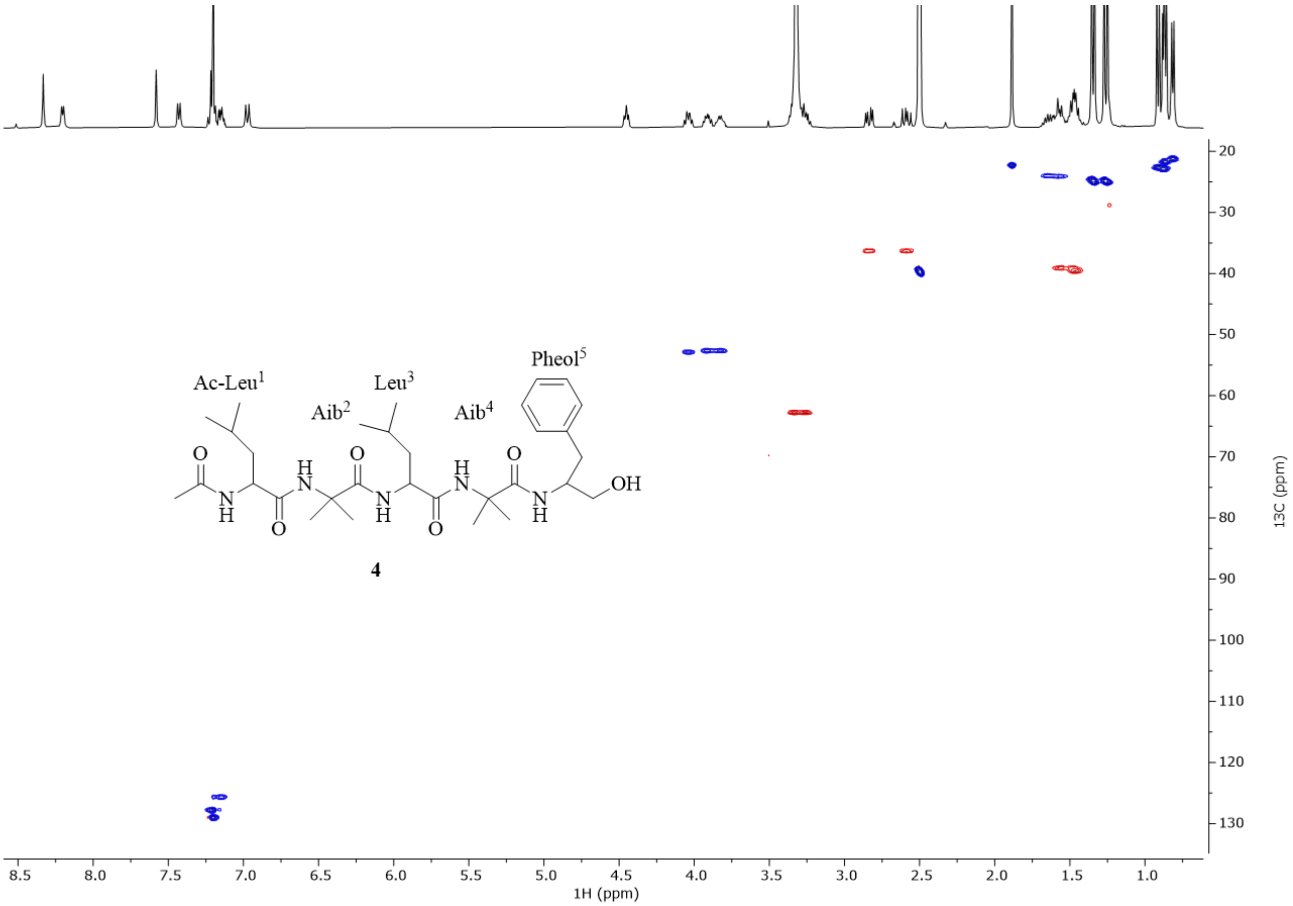

Figure S32. ^{1}H, ^{13}C HSQC spectrum of natural peptaibolin (**4**) (400 MHz, DMSO-*d*$_6$).

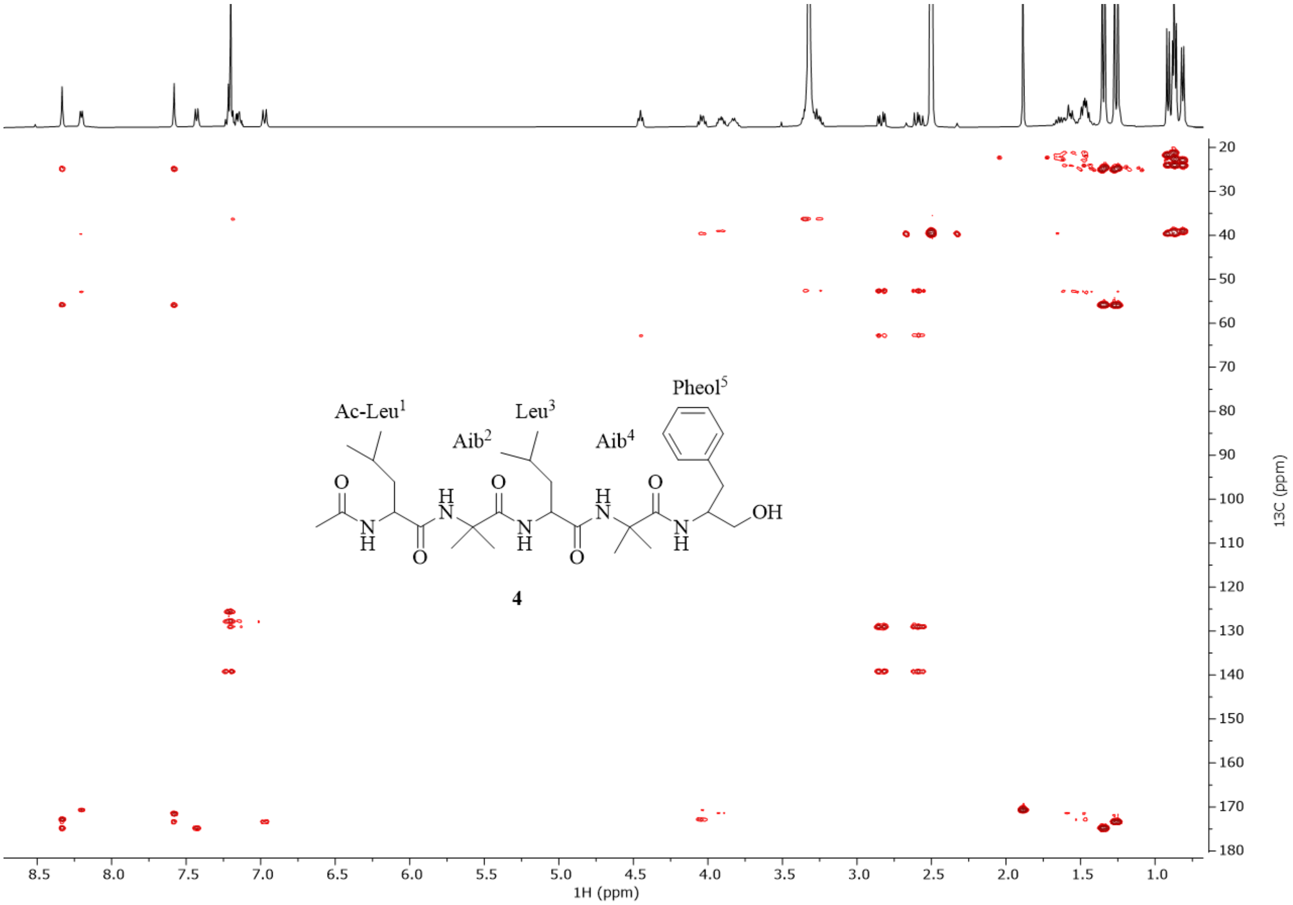

Figure S33. ^{1}H, ^{13}C HMBC spectrum of natural peptaibolin (**4**) (400 MHz, DMSO-*d*$_6$).

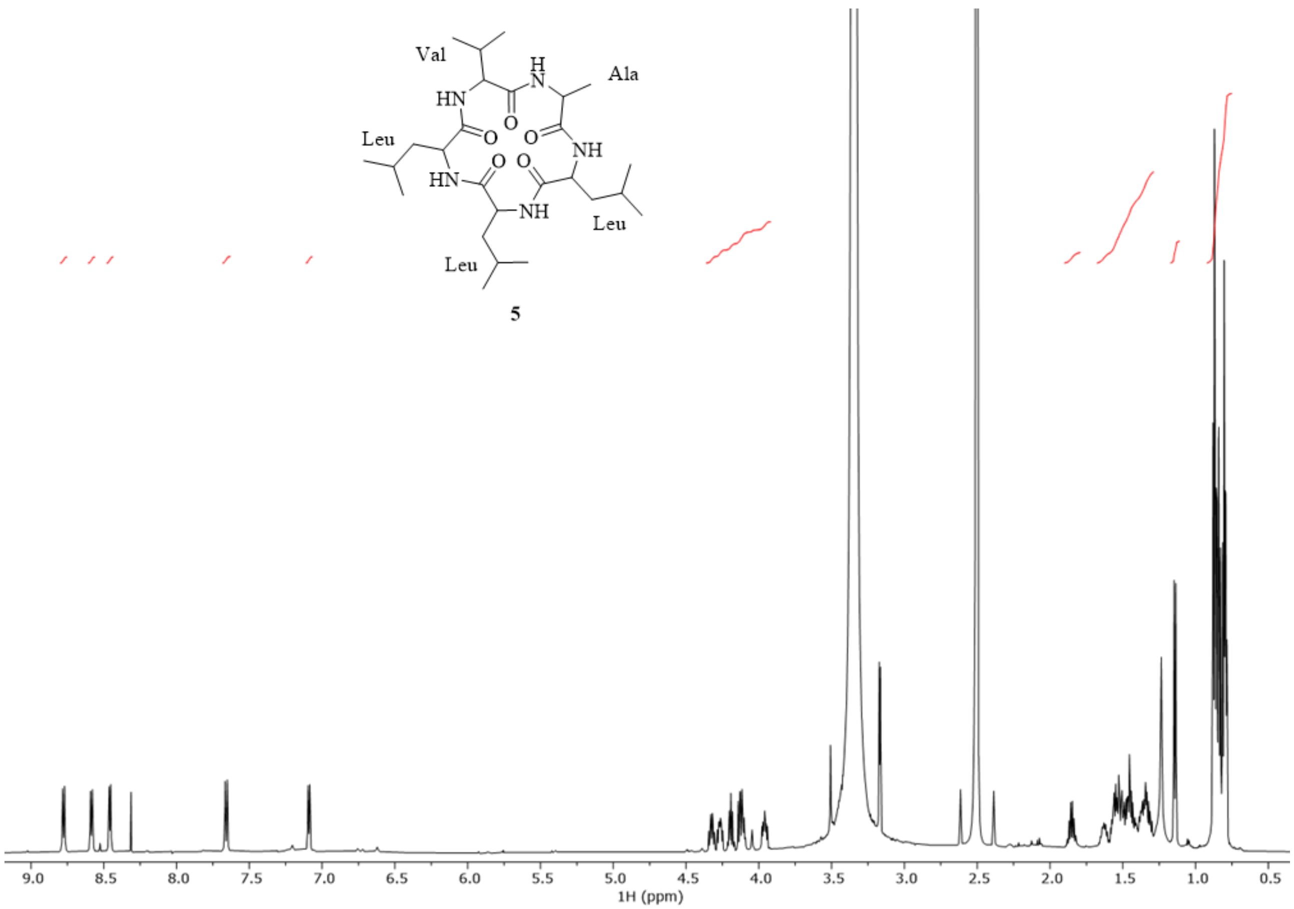

Figure S34. ^{1}H NMR spectrum of natural chrysosporide (**5**) (600 MHz, DMSO-d_6).

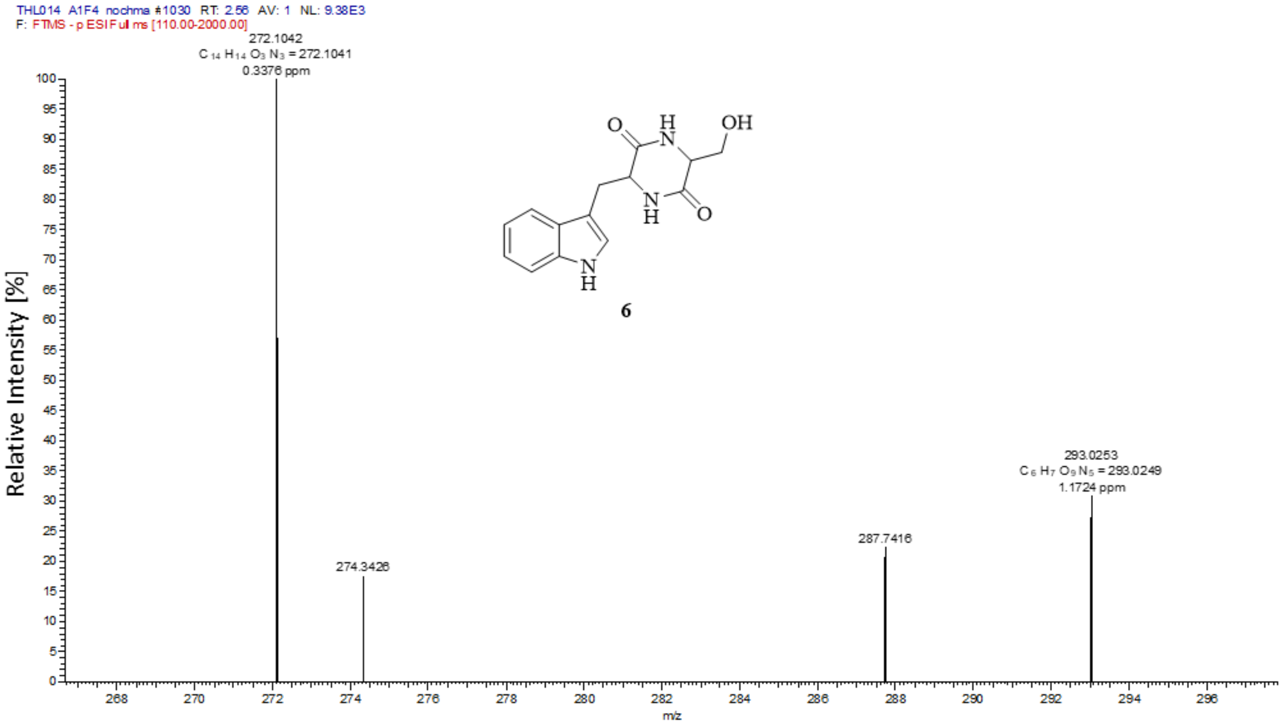

Figure S35. Negative ion mass spectrum of natural c(Trp-Ser) (**6**).

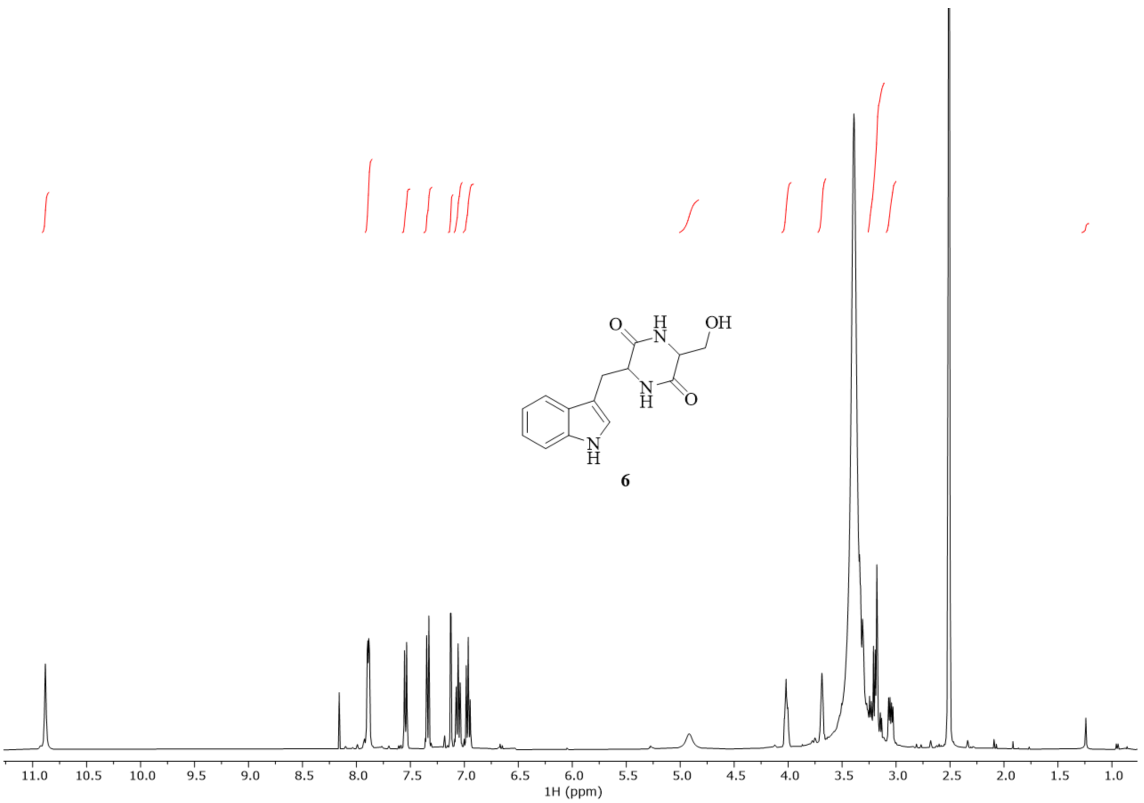

Figure S36. ^{1}H NMR spectrum of natural c(Trp-Ser) (**6**) (400 MHz, DMSO-d_6).

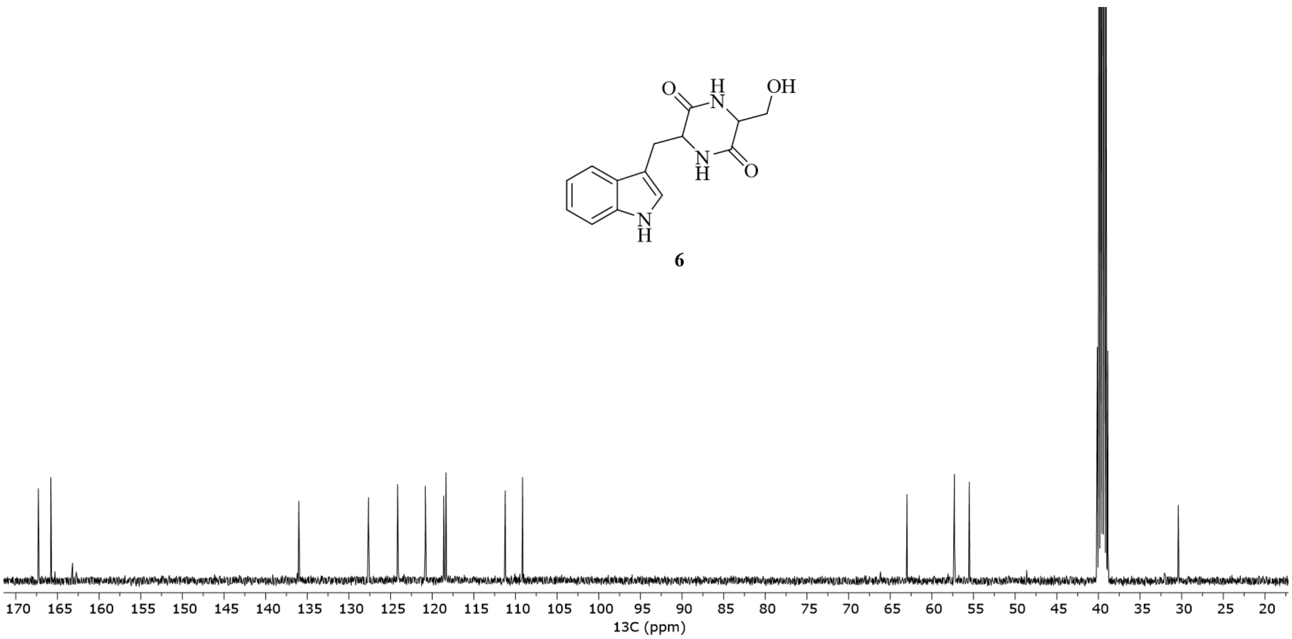

Figure S37. ^{13}C NMR spectrum of natural c(Trp-Ser) (**6**) (100 MHz, DMSO-d_6).

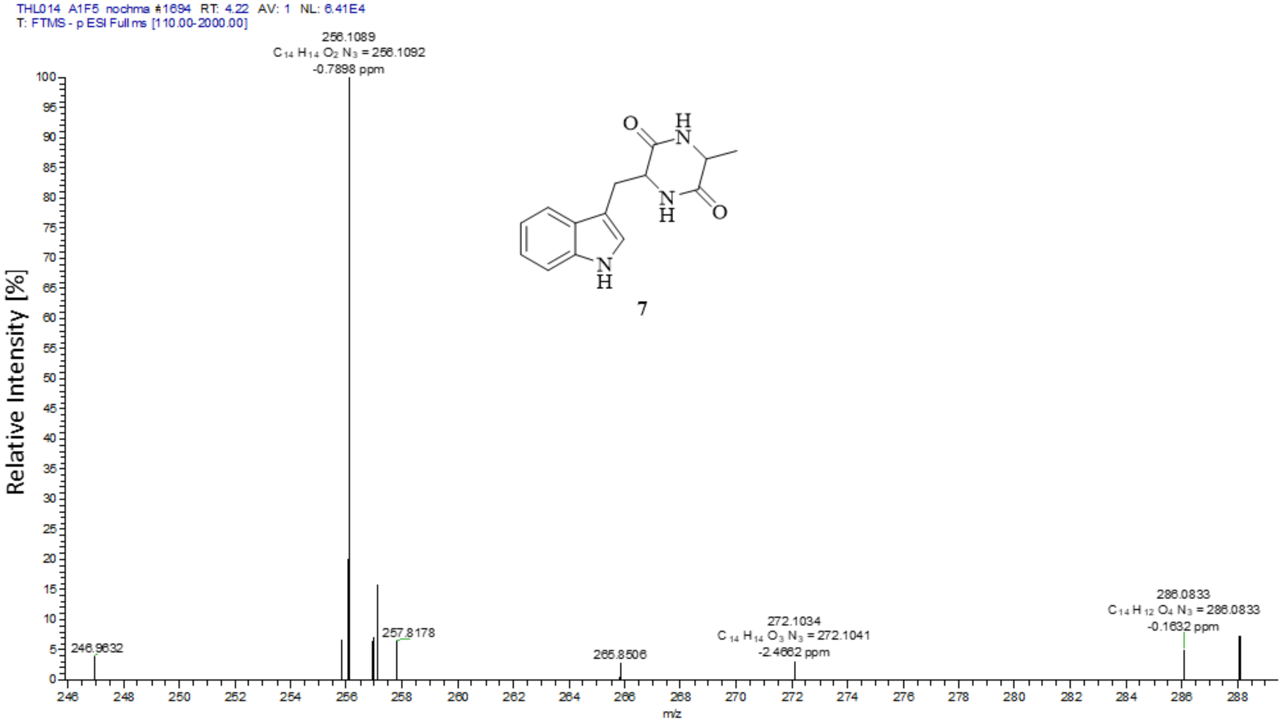

Figure S38. Negative ion mass spectrum of natural c(Trp-Ala) (**7**).

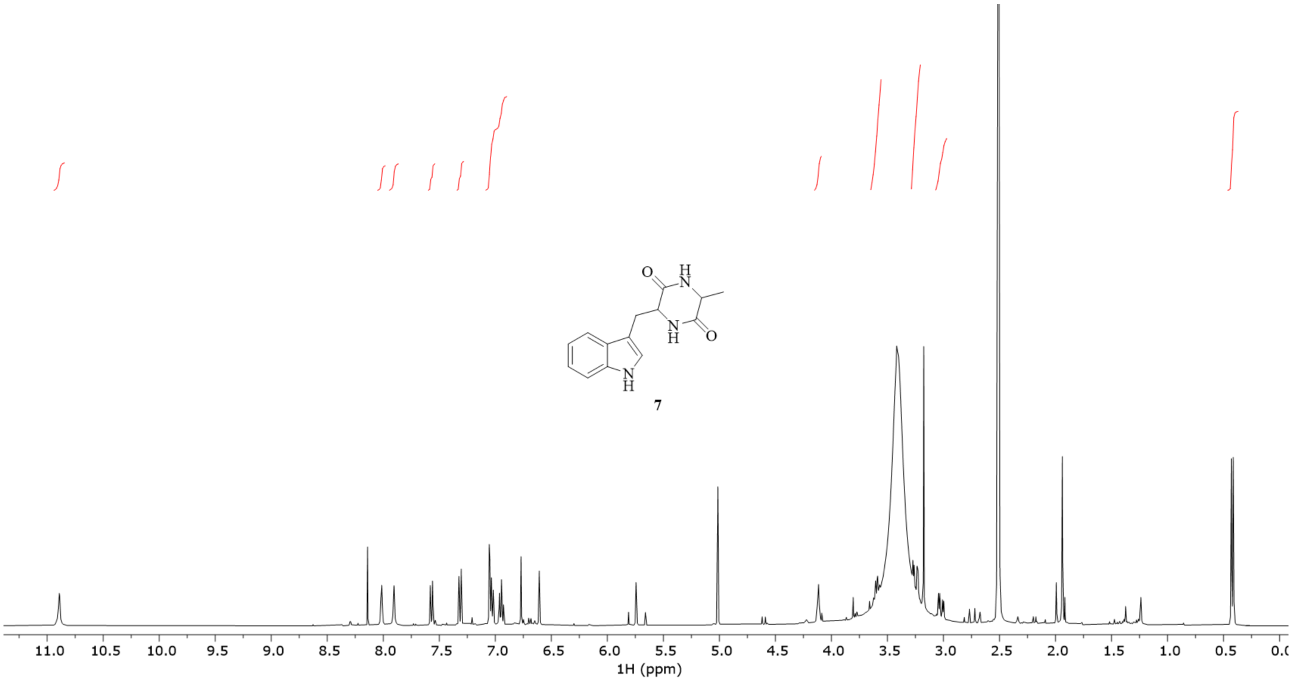

Figure S39. ^{1}H NMR spectrum of natural c(Trp-Ala) (**7**) (400 MHz, DMSO-d_6).

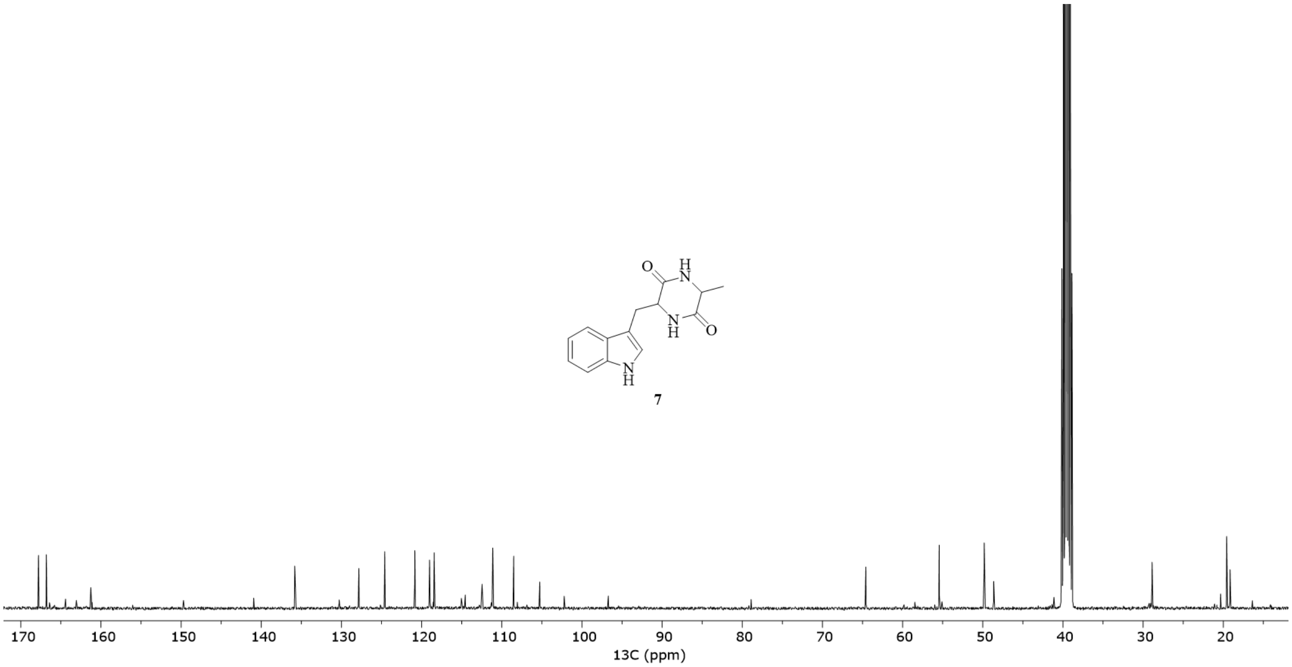

Figure S40. ^{13}C NMR spectrum of natural c(Trp-Ala) (**7**) (100 MHz, DMSO-d_6).

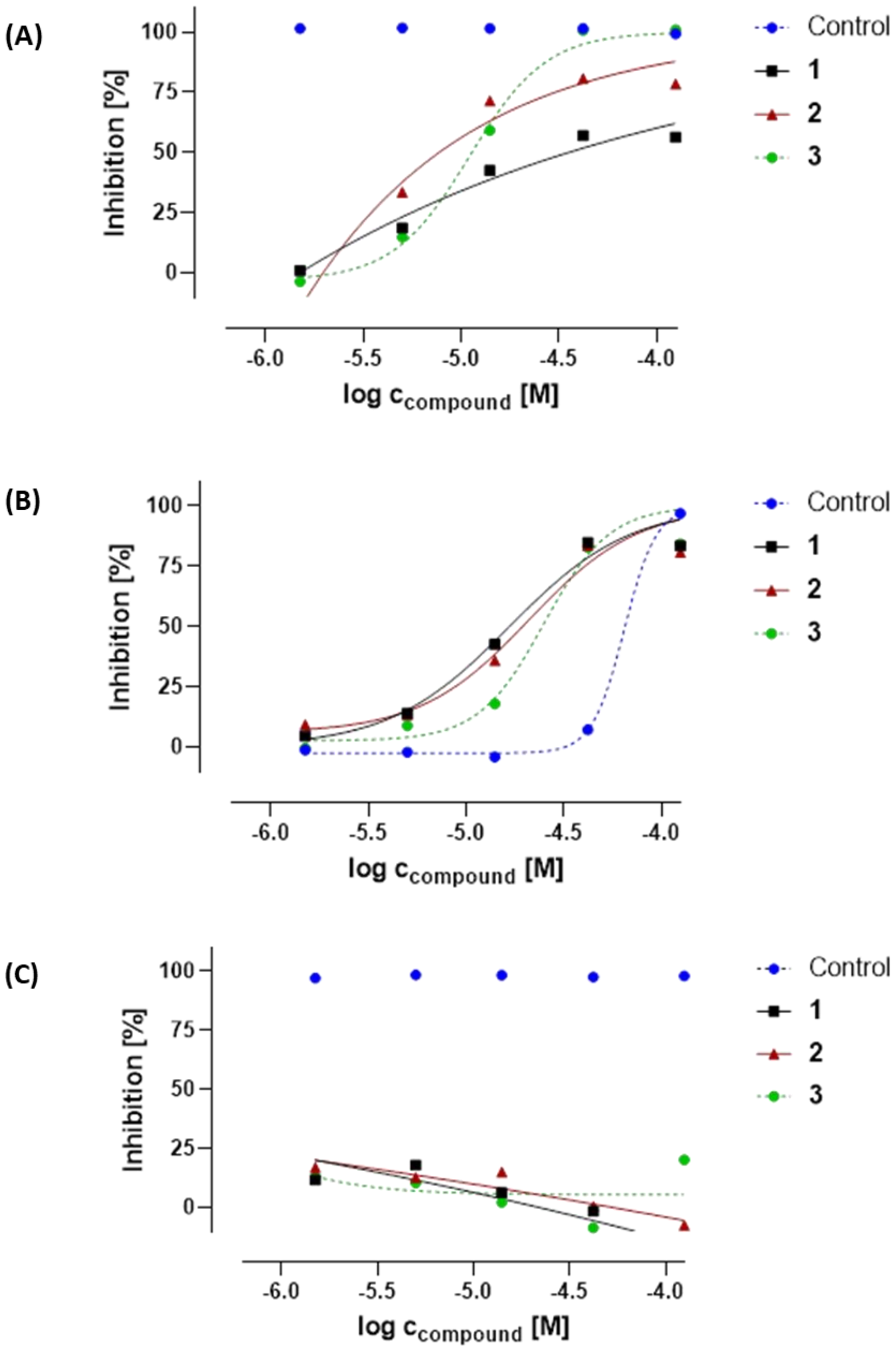

Figure S41 Antiphytopathogenic activity of ampullosporin F (**1**), G (**2**), and A (**3**) against: (**A**) *B. cinerea*, Control = Epoxiconazole; (**B**) *P. infestans*, Control = Terbinafine; and (**C**) *S. tritici*, Control = Epoxiconazole.

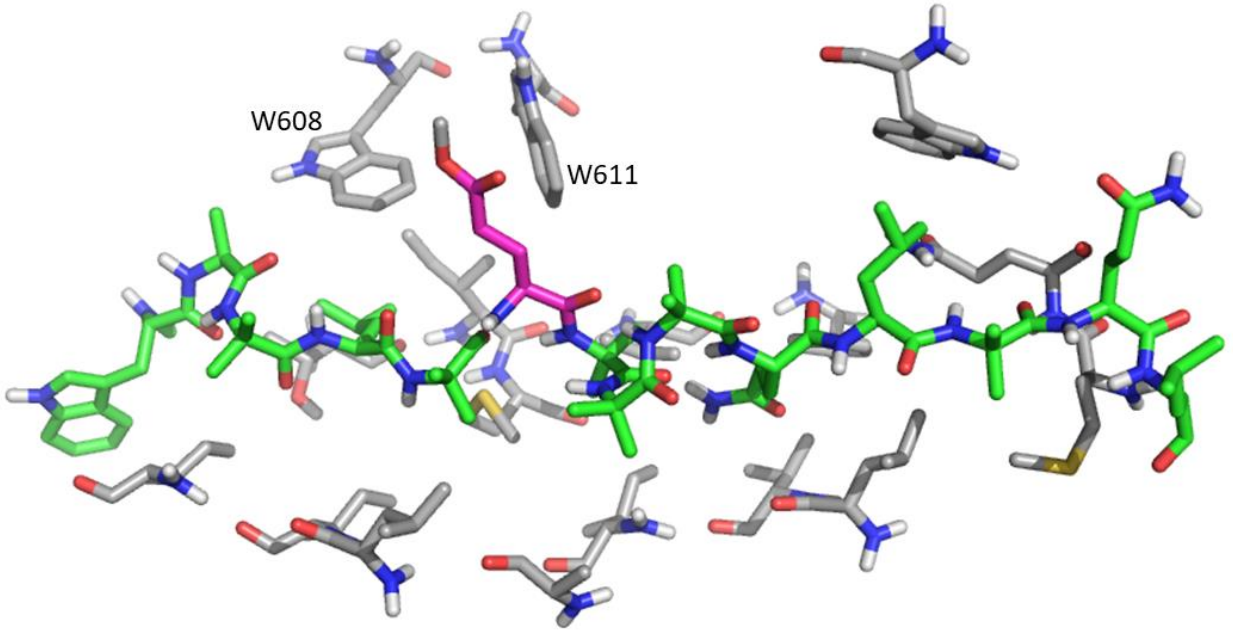

Figure S42 Docking arrangements of ampullosporin G (**2**) in the proposed binding site in the hydrophobic cleft between NMDA receptor subunits GluN1 and GluN2A, as modeled based on protein databank entry 6IRA.

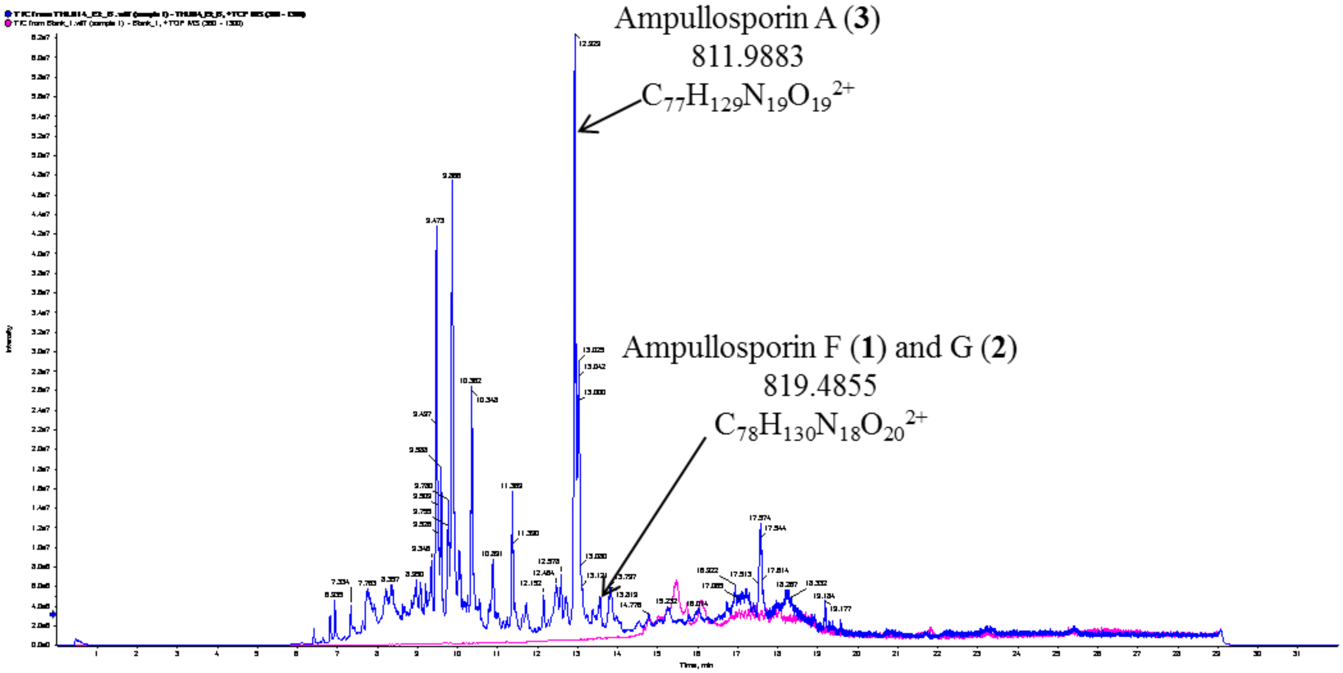

Figure S43. LC-HRMS spectrum of an enriched fraction (in blue) from the ethanol extract of *S. ampullosporum* strain KSH 534 and a blank sample (in pink)

Table S1. Positive and negative ion ESI-HRMS^n data of ampullosporin F (**1**) and G (**2**).

cpd	(+)/(-) scan mode [*m/z* (NCE in%)]	*m/z* (fragment ion, relative intensity in %, error in ppm)
**1**	(+) full MS	1637.9608 (**[M+H]**$^+$, 14, 1.0), 819.4849 (**[M+2H]**$^{2+}$, 100, 0.0)
	(+) MS^2 [**1638.0** (**35**)]	1619.9515 (**[M+H-H**$_2$**O]**$^+$, 47, 0.2), 1392.7877 ($\mathbf{b}_{13}^+$, 100, 0.6), 1307.7346 ($\mathbf{b}_{12}^+$, 21, 0.9), 1194.6510 ($\mathbf{b}_{11}^+$, 41, 0.6), 1051.5927 ($\mathbf{b}_{10}^+$, 82, 0.8), 966.5402 ($\mathbf{b}_{9}^+$, 39, 0.6), 881.4879 ($\mathbf{b}_{8}^+$, 25, 0.1), 796.4326 ($\mathbf{b}_{7}^+$, <1, 3.2), 668.3765 ($\mathbf{b}_{6}^+$, 27, 0.2), 583.3235 ($\mathbf{b}_{5}^+$, 7, 0.5), 470.2393 ($\mathbf{b}_{4}^+$, 2, 1.0)
	(+) MS^2 [**819.5** (**30**)]	1392.7917 ($\mathbf{b}_{13}^+$, 8, 3.9), 1194.6517 ($\mathbf{b}_{11}^+$, 8, 0.0), 1168.7280 ($\mathbf{y}_{11}^+$, 11, 1.7), 1055.6453 ($\mathbf{y}_{10}^+$, 17, 0.6), 1051.5925 ($\mathbf{b}_{10}^+$, 35, 0.9), 970.5923 ($\mathbf{y}_{9}^+$, 100, 0.9), 966.5395 ($\mathbf{b}_{9}^+$, 38, 1.3), 881.4879 ($\mathbf{b}_{8}^+$, 41, 0.1), 842.5358 ($\mathbf{y}_{8}^+$, 3, 1.4), 757.4816 ($\mathbf{y}_{7}^+$, 29, 0.2), 672.4281 ($\mathbf{y}_{6}^+$, 25, 1.4), 668.3765 ($\mathbf{b}_{6}^+$, 77, 0.2), 587.3766 ($\mathbf{y}_{5}^+$, 19, 0.9), 583.3242 ($\mathbf{b}_{5}^+$, 16, 0.6), 470.2393 ($\mathbf{b}_{4}^+$, 17, 1.1), 444.3214 ($\mathbf{y}_{4}^+$, <1, 7.6), 385.1870 ($\mathbf{b}_{3}^+$, 6, 0.1), 300.1361 ($\mathbf{b}_{2}^+$, <1, 6.2)
	(+) MS^3 [819.5 (30)→**970.6** (**25**)]	952.5790 ($\mathbf{y}_{9}^+$**-H**$_2$**O**, 60, 3.8), 853.4770 ($\mathbf{y}_{9}^+$**-aa(15)**, 72, 1.0), 725.4192 ($\mathbf{y}_{9}^+$**-aa(14-15)**, 100, 0.0), 640.3662 ($\mathbf{y}_{9}^+$**-aa(13-15)**, 35, 0.5), 527.2811 ($\mathbf{y}_{9}^+$**-aa(12-15)**, 7, 2.4)
	(+) MS^3 [819.5 (45)→**470.2** (**17**)]	385.1874 ($\mathbf{b}_{3}^+$, 100, 1.2)
	(+) MS^4 [819.5 (45)→470.2 (17) →**385.2** (**17**)]	357.1922 ($\mathbf{b}_{3}^+$**-CO**, 100, 1.1), 300.1345 ($\mathbf{b}_{2}^+$, 86, 0.6)
	(+) MS^5 [819.5 (45)→470.2 (17) →385.2 (17)→**300.1** (**23**)]	229.0977 ($\mathbf{b}_{1}^+$, 100, 1.3)
**1**	(-) full MS	1681.9465 (**[M-H+HCOOH]**$^-$, 21, 3.5), 1635.9458 (**[M-H]**$^-$, 100, 1.2), 817.4680 (**[M-2H]**$^{2-}$, 11, 2.5)
	(-) MS^2 [**1635.9** (**35**)]	1617.9349 (**[M-H-H**$_2$**O]**$^-$, 6, 1.5), 1407.8525 ($\mathbf{y}_{14}^-$, 4, 3.9), 1390.7782 ($\mathbf{b}_{13}^-$, <1, 3.0), 1336.8179 ($\mathbf{y}_{13}^-$, 3, 3.0), 1251.7668 ($\mathbf{y}_{12}^-$, 4, 1.1), 1166.7102 ($\mathbf{y}_{11}^-$, 3, 4.6), 1053.6372 ($\mathbf{y}_{10}^-$, 1, 4.1), 1049.5755 ($\mathbf{b}_{10}^-$, 100, 3.3), 968.5731 ($\mathbf{y}_{9}^-$, <1, 5.7), 964.5287 ($\mathbf{b}_{9}^-$, <1, 2.6), 936.5289 ($\mathbf{b}_{9}^-$**-CO**, 66, 2.5), 879.4720 ($\mathbf{b}_{8}^-$, <1, 1.6), 794.4152 ($\mathbf{b}_{7}^-$, <1, 6.9), 666.3598 ($\mathbf{b}_{6}^-$, 2, 3.5), 553.3342 (y_5^-**- CH**$_3$**OH**, 42, 2.4)
**2**	(+) full MS	1637.9625 (**[M+H]**$^+$, 15, 0.0), 819.4860 (**[M+2H]**$^{2+}$, 100, 1.4)
	(+) MS^2 [**1638.0** (**35**)]	1619.9508 (**[M+H-H**$_2$**O]**$^+$, 55, 0.7), 1392.7875 ($\mathbf{b}_{13}^+$, 67, 0.8), 1307.7357 ($\mathbf{b}_{12}^+$, 27, 0.1), 1194.6440 ($\mathbf{b}_{11}^+$, <1, 6.4), 1066.5923 ($\mathbf{b}_{10}^+$, 100, 0.8), 981.5396 ($\mathbf{b}_{9}^+$, 50, 0.8), 896.4871 ($\mathbf{b}_{8}^+$, 32, 0.6), 811.4340 ($\mathbf{b}_{7}^+$, <1, 1.0), 668.3763 ($\mathbf{b}_{6}^+$, 29, 0.4), 583.3235 ($\mathbf{b}_{5}^+$, 10, 0.6), 470.2400 ($\mathbf{b}_{4}^+$, 3, 0.4)
	(+) MS^2 [**819.5** (**30**)]	1520.8486 ($\mathbf{b}_{14}^+$, 2, 3.9), 1392.7886 ($\mathbf{b}_{13}^+$, 39, 0.1), 1307.7392 ($\mathbf{b}_{12}^+$, 2, 2.6), 1194.6516 ($\mathbf{b}_{11}^+$, 5, 0.1), 1168.7295 ($\mathbf{y}_{11}^+$, 8, 0.4), 1066.5919 ($\mathbf{b}_{10}^+$, 82, 1.2),

		1055.6425 ($\mathbf{y_{10}^{+}}$, 14, 3.2), 981.5388 ($\mathbf{b_{9}^{+}}$, 88, 1.6), 970.5915 ($\mathbf{y_{9}^{+}}$, 100, 1.7), 896.4869 ($\mathbf{b_{8}^{+}}$, 79, 0.8), 827.5331 ($\mathbf{y_{8}^{+}}$, 2, 2.2), 742.4813 ($\mathbf{y_{7}^{+}}$, 52, 1.1), 668.3763 ($\mathbf{b_{6}^{+}}$, 91, 0.5), 657.4290 ($\mathbf{y_{6}^{+}}$, 62, 0.8), 583.3232 ($\mathbf{b_{5}^{+}}$, 18, 1.2), 572.3767 ($\mathbf{y_{5}^{+}}$, 44, 0.1), 470.2396 ($\mathbf{b_{4}^{+}}$, 17, 0.4), 444.3169 ($\mathbf{y_{4}^{+}}$, 4, 2.7), 385.1872 ($\mathbf{b_{3}^{+}}$, 6, 0.4), 300.1368 ($\mathbf{b_{2}^{+}}$, 1, 8.5), 246.1820 ($\mathbf{y_{2}^{+}}$, 2, 3.3)
	(+) MS^3 [819.5 (30)→**970.6** (**25**)]	952.5803 ($\mathbf{y_{9}^{+}}$**-**$\mathbf{H_2O}$, 56, 2.4), 853.4765 ($\mathbf{y_{9}^{+}}$**-aa(15)**, 77, 1.5), 725.4192 ($\mathbf{y_{9}^{+}}$**-aa(14-15)**, 100, 0.0), 640.3657 ($\mathbf{y_{9}^{+}}$**-aa(13-15)**, 34, 1.2), 527.2846 ($\mathbf{y_{9}^{+}}$**-aa(12-15)**, 9, 4.1), 399.2275 ($\mathbf{y_{9}^{+}}$**-aa(11-15)**, <1, 9.3)
	(+) MS^3 [819.5 (45)→**470.2** (**17**)]	385.1872 ($\mathbf{b_{3}^{+}}$, 100, 0.4)
	(+) MS^4 [819.5 (45)→470.2 (17) →**385.2** (**17**)]	357.1922 ($\mathbf{b_{3}^{+}}$**-CO**, 100, 0.6), 300.1342 ($\mathbf{b_{2}^{+}}$, 90, 0.3)
	(+) MS^5 [819.5 (45)→470.2 (17) →385.2 (17)→**300.1** (**23**)]	229.0977 ($\mathbf{b_{1}^{+}}$, 100, 2.6)
**2**	(-) full MS	1681.9472 (**[M-H+HCOOH]**$^-$, 20, 3.7), 1635.9453 (**[M-H]**$^-$, 100, 1.6), 817.4681 (**[M-2H]**$^{2-}$, 7, 2.7)
	(-) MS^2 [**1635.9** (**35**)]	1617.9287 (**[M-H-**$\mathbf{H_2O}$**]**$^-$, <1, 5.4), 1407.8543 ($\mathbf{y_{14}^{-}}$, <1, 2.7), 1390.7803 ($\mathbf{b_{13}^{-}}$, <1, 4.5), 1336.8093 ($\mathbf{y_{13}^{-}}$, <1, 8.7), 1251.7572 ($\mathbf{y_{12}^{-}}$, <1, 8.8), 1192.6478 ($\mathbf{b_{11}^{-}}$, <1, 8.9), 1166.7143 ($\mathbf{y_{11}^{-}}$, 3, 1.0), 1064.5835 ($\mathbf{b_{10}^{-}}$, <1, 8.9), 1053.6284 ($\mathbf{y_{10}^{-}}$, 1, 2.8), 968.5767 ($\mathbf{y_{9}^{-}}$, <1, 1.3), 936.5499 ($\mathbf{y_{9}^{-}}$**-** $\mathbf{CH_3OH}$, 100, 2.7), 825.5215 (y_{8}^{-}, <1, 1.4), 740.4655 (y_{7}^{-}, 3, 2.9), 666.3651 ($\mathbf{b_{6}^{-}}$, <1, 4.5), 655.4136 (y_{6}^{-}, 3, 1.8), 570.3615 (y_{5}^{-}, 2, 1.1), 553.3342 (y_{5}^{-}**-** $\mathbf{NH_3}$, 3, 2.4)

Table S2. Positive ion ESI-HRMS^n data of ampullosporin A (**3**), peptaibolin (**4**) and chrysosporide G (**5**).

cpd	(+)/(-) scan mode [*m*/*z* (NCE in%)]	*m*/*z* (fragment ion, relative intensity in %, error in ppm)
**3**	(+) full MS	1622.9628 (**[M+H]**$^+$, 52, 0.0), 811.9851 (**[M+2H]**$^{2+}$, 100, 0.1)
	(+) MS^2 [**1623.0** (**45**)]	1604.9509 (**[M+H-H$_2$O]**$^+$, 94, 0.8), 1377.7959 ($\mathbf{b}_{13}^{+}$, 15, 5.1), 1292.7404 ($\mathbf{b}_{12}^{+}$, 29, 3.3), 1051.5931 ($\mathbf{b}_{10}^{+}$, 51, 0.3), 966.5410 ($\mathbf{b}_{9}^{+}$, 45, 0.2), 881.4872 ($\mathbf{b}_{8}^{+}$, 56, 0.9), 668.3757 ($\mathbf{b}_{6}^{+}$, 100, 1.4), 583.3231 ($\mathbf{b}_{5}^{+}$, 34, 1.3), 470.2400 ($\mathbf{b}_{4}^{+}$, 16, 0.5)
	(+) MS^2 [**812.0** (**45**)]	1377.7928 ($\mathbf{b}_{13}^{+}$, 16, 2.9), 1292.7458 ($\mathbf{b}_{12}^{+}$, 1, 7.5), 1153.7329 ($\mathbf{y}_{11}^{+}$, 8, 2.2), 1051.5951 ($\mathbf{b}_{10}^{+}$, 39, 1.5), 1040.6482 ($\mathbf{y}_{10}^{+}$, 13, 1.9), 966.5419 ($\mathbf{b}_{9}^{+}$, 36, 1.2), 955.5953 ($\mathbf{y}_{9}^{+}$, 83, 1.9), 881.4892 ($\mathbf{b}_{8}^{+}$, 36, 1.4), 742.4830 ($\mathbf{y}_{7}^{+}$, 34, 1.1), 668.3775 ($\mathbf{b}_{6}^{+}$, 100, 1.3), 657.4302 ($\mathbf{y}_{6}^{+}$, 25, 1.3), 583.3246 ($\mathbf{b}_{5}^{+}$, 42, 1.2), 572.3776 ($\mathbf{y}_{5}^{+}$, 24, 1.7), 470.2406 ($\mathbf{b}_{4}^{+}$, 46, 1.6), 444.3177 ($\mathbf{y}_{4}^{+}$, 4, 0.9), 385.1878 ($\mathbf{b}_{3}^{+}$, 21, 2.0), 331.2343 ($\mathbf{y}_{3}^{+}$, <1, 1.0), 300.1360 ($\mathbf{b}_{2}^{+}$, 3, 5.6), 246.1816 ($\mathbf{y}_{2}^{+}$, 2, 1.7), 229.0992 ($\mathbf{b}_{1}^{+}$, <1, 9.1)
**4**	(+) full MS	590.3892 (**[M+H]**$^+$, 100, 3.4)
	(+) MS^2 [**590.4** (**35**)]	572.3789 (**[M+H-H$_2$O]**$^+$, 27, 3.1), 439.2899 ($\mathbf{b}_{4}^{+}$, 100, 3.5), 354.2377 ($\mathbf{b}_{3}^{+}$, 18, 2.8), 241.1542 ($\mathbf{b}_{2}^{+}$, 4, 2.1)
**5**	(+) full MS	510.3649 (**[M+H]**$^+$, 100, 0.2), 532.3468 (**[M+Na]**$^+$, 28, 0.2)
	(+) MS^2 [**510.4** (**35**)]	492.3546 (**[M+H-H$_2$O]**$^+$, 10, 0.3), 482.3700 (**[M+H-CO]**$^+$, 90, 0.2), 465.3435 (**[M+H-CO-NH$_3$]**$^+$, 43, 0.2), 411.2961 ($\mathbf{b}_{4AV}$, 85, 1.1), 397.2806 ($\mathbf{b}_{4LL}$/$\mathbf{b}_{4LL}$*/$\mathbf{b}_{4VL}$, 100, 0.8), 383.3018 ($\mathbf{a}_{4AV}$, 12, 0.4), 369.2861 ($\mathbf{a}_{4LL}$/$\mathbf{a}_{4LL}$*/$\mathbf{a}_{4VL}$, 26, 0.3), 366.2754 (**[a$_{4AV}$-NH$_3$]**$^+$, 4, 0.8), 352.2596 (**[a$_{4LL}$/a$_{4LL}$*/a$_{4VL}$-NH$_3$]**$^+$, 9, 0.5), 340.2596 ($\mathbf{b}_{3LA}$, 2, 0.5), 326.2433 ($\mathbf{b}_{3LL}$, 4, 1.5), 298.2127 ($\mathbf{b}_{3AV}$, 23, 0.6), 284.1970 ($\mathbf{b}_{3LL}$*/$\mathbf{b}_{3VL}$, 18, 0.5), 227.1757 ($\mathbf{b}_{2LA}$/$\mathbf{b}_{2LL}$, 3, 1.5), 213.1600 ($\mathbf{b}_{2LL}$*, 1, 1.3), 199.1807 ($\mathbf{a}_{2LL}$/$\mathbf{a}_{2LA}$, 2, 1.1), 185.1647 ($\mathbf{a}_{2LL}$*, 1, 1.0), 185.1286 ($\mathbf{b}_{2AV}$, 1, 0.9), 171.1125 ($\mathbf{b}_{2VL}$, <1, 1.7), 157.1337 ($\mathbf{a}_{2AV}$, 1, 0.8)

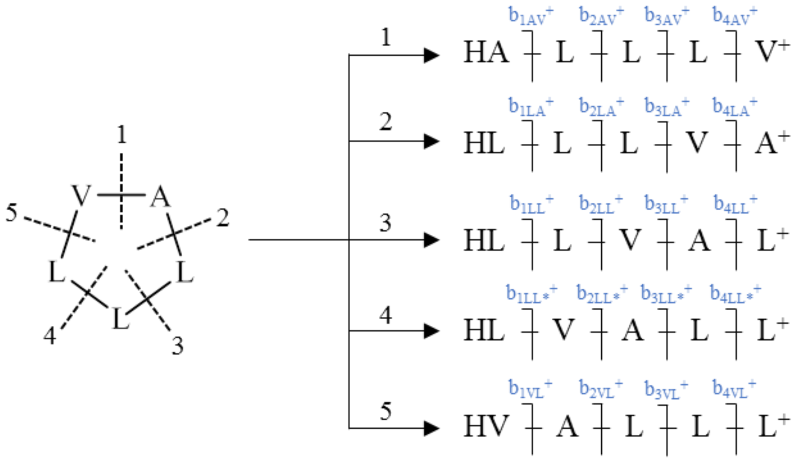

Supplement: Supplementary file 1 [file ijms-22-12718-s001.zip › ijms-1463192-supplementary.pdf]
